# Supplementary material for: BioPreDyn-bench: a suite of benchmark problems for dynamic modelling in systems biology
Source: BMC Syst Biol. 2015 Feb 20;9:8. doi: 10.1186/s12918-015-0144-4 (PMC4342829; doi:10.1186/s12918-015-0144-4)
Supplement: Additional file 1 — Describes the benchmark models in further detail and reports additional results. [file 12918_2015_144_MOESM1_ESM.pdf]

BioPreDyn-bench: a suite of benchmark problems for dynamic modelling in systems biology – Additional File 1 (supplementary information)

Alejandro F. Villaverde[1], David Henriques[1,2], Kieran Smallbone[3], Sophia Bongard[4], Joachim Schmid[4], Damjan Cicin-Sain[5,6], Anton Crombach[5,6], Julio Saez-Rodriguez[2], Klaus Mauch[4], Eva Balsa-Canto[1], Pedro Mendes[3], Johannes Jaeger[5,6*], Julio R. Banga[1*]

1 Bioprocess Engineering Group, IIM-CSIC, Eduardo Cabello 6, 36208 Vigo, Spain
2 European Molecular Biology Laboratory, European Bioinformatics Institute (EMBL-EBI), Wellcome Trust Genome Campus, Hinxton, Cambridge CB10 1SD, UK
3 School of Computer Science, Manchester Centre for Integrative Systems Biology, The University of Manchester, 131 Princess Street, Manchester, M1 7DN, United Kingdom
4 Insilico Biotechnology AG, Meitnerstraße 8, 70563 Stuttgart, Germany
5 EMBL/CRG Research Unit in Systems Biology, Centre for Genomic Regulation (CRG), Dr. Aiguader 88, 08003 Barcelona, Spain
6 Universitat Pompeu Fabra (UPF), Plaça de la Mercé, 10, 08002 Barcelona, Spain

∗ E-mail: julio@iim.csic.es, yogi.jaeger@crg.eu

February 9, 2015

Contents

1 Implementations and files **1**

2 Requirements, installation, and instructions **9**
2.1 COPASI . . . 9
2.2 Matlab . . . 10
2.2.1 Pre-compiled (fixed) vs. editable dynamics in the Matlab implementations . . . 11
2.3 AMIGO . . . 11
2.4 C . . . 12
2.4.1 Requirements and installation instructions for Windows . . . 13
2.4.2 Requirements and installation instructions for Linux and macOSx . . . 14
2.4.3 Brief overview of the template file . . . 15
2.4.4 Brief Note on Compiler Versions . . . 15

3 Testing reproducibility **16**

4 Models: parameters, outputs, and data **20**
4.1 B1: *S. cerevisiae* . . . 20
4.2 B2: dynamic CCM of *E. coli* . . . 55
4.3 B3: regulation of the CCM of *E. coli* . . . 61
4.4 B4: CHO . . . 66
4.5 B5: signal transduction logic model . . . 70
4.6 B6: *Drosophila* . . . 73

5 Typical results: parameter estimation with local methods **83**

6 Typical results: parameter estimation with eSS **85**
6.1 B1: *S. cerevisiae* . . . 87
6.2 B2: dynamic CCM of *E. coli* . . . 89
6.3 B3: regulation of the CCM of *E. coli* . . . 91
6.4 B4: CHO . . . 93
6.5 B5: signal transduction logic model . . . 95
6.6 B6: *Drosophila* . . . 97

1 Implementations and files

The benchmarks in the BioPreDyn-bench collection are implemented in the following formats:

1. ready-to-run implementations in Matlab, AMIGO (a Matlab toolbox), and COPASI.
2. core implementations in C (which only need the user to write a small program as optimization driver, therefore allowing the use of any optimization code which can be interfaced with C). Example programs are given to facilitate this step.
3. partial implementations (systems dynamics plus initial conditions) in SBML (so they can be imported into any of the many software packages that support this format).

A description of these formats is given below:

- SBML [1] is a model representation format designed for systems biology applications. Please note that the SBML files only contain information about the model dynamics, not about the parameter estimation problem (the parameters to estimate are not specified, the objective function is not defined, data is not included, etc).
- C is a general purpose programming language. The C implementations provide model dynamics and calculation of the objective function value. Some aspects of the optimization problem have to be set by the user (initial point, parameter bounds, solver, etc).
- MATLAB is a high-level language and interactive environment for numerical computation, visualization, and programming. The MATLAB implementations, like the C ones, provide model dynamics and calculation of the objective function value. Additionally, example optimization files are provided that define a parameter estimation problem (specifying the initial point, parameter bounds, solver, and solver options), thus making the Matlab implementations ready-to-run.
- AMIGO [2] is a toolbox that works under Matlab and covers all the steps of the iterative identification procedure: local and global sensitivity analysis, local and global ranking of parameters, parameter estimation, identifiability analysis and optimal experimental design. The AMIGO implementations are ready-to-run: all the aspects of the parameter estimation problem are defined.
- COPASI [3] is a software application for simulation and analysis of biochemical networks and their dynamics. COPASI is a stand-alone program that supports models in the SBML standard. The COPASI implementations are ready-to-run: all the aspects of the parameter estimation problem are defined.

We indicate in Table S1 below, for each benchmark implementation, the operating system where it can be executed and additional software requirements (indicated inside parentheses).

For optimization purposes (parameter estimation), ready-to-run versions are provided in Matlab, AMIGO and COPASI. Additionally, the user can set up a parameter estimation problem in C using the corresponding files. A list of all the model files is given in Tables S2–S7. More detailed information is provided in the README files included with every implementation.

Table S1. Implementations and operating systems

Implementation	B1	B2	B3
AMIGO	Win(ML32), Lin(ML64)	Win(ML32), Lin(ML64)	Win(ML32), Lin(ML64)
C	Win(MCR), Lin(MCR)	Win(MCR), Lin(MCR)	Win(MCR), Lin(MCR)
COPASI	Win, Lin, OSX	Win, Lin, OSX	Win, Lin, OSX
Matlab	Win(ML32), Lin(ML64)	Win(ML32), Lin(ML64)	Win(ML32), Lin(ML64)
SBML	Win, Lin, OSX	Win, Lin, OSX	Win, Lin, OSX
Implementation	B4	B5	B6
AMIGO	Win(ML32), Lin(ML64)	Win(ML32), Lin(ML64)	Lin(ML32,ML64)
C	Win(MCR), Lin(MCR)	Win(MCR), Lin(MCR)	Lin(MCR)
COPASI	Win, Lin, OSX	-	-
Matlab	Win(ML32), Lin(ML64)	Win(ML32), Lin(ML64)	Lin(ML32,ML64)
SBML	Win, Lin, OSX	Win, Lin, OSX	-

Abbreviations used in the table:
Win: Windows; tested with Windows 7 64-Bit
Lin: Linux; tested with openSUSE 64-Bit
OSX: Mac OSX
ML32: Matlab 32-Bit version, tested with Matlab R2011 32-Bit for Windows
ML64: Matlab 64-Bit version, tested with Matlab R2008a 64-Bit for Linux
MCR: Matlab Compiler Runtime, available at `http://www.mathworks.es/products/compiler/mcr/`
Additional notes:

- Matlab Optimization Toolbox might be needed for certain local solvers.
- C implementations: see further details in the corresponding README files regarding further requirements, installation and compilation instructions.

Table S2. Benchmark B1: File list

Folder	Subfolder	FILES
AMIGO		b1_amigo.m<br>pnom.mat<br>README_AMIGO_B1.txt
C		b1_fullC_template.c<br>compile_c.bat<br>fullC_data.mat<br>Makefile<br>nominalpars.h<br>README_C_B1.txt
COPASI		b1.cps<br>README_COPASI_B1.txt<br>yeast_exp.csv<br>yeast_param.csv
Matlab		b1.m<br>b1_obj.m<br>b1_test.m<br>cvodesg_b1.mexa64<br>cvodesg_b1.mexw32<br>README_MATLAB_B1.txt
	dynamics_in_Matlab	b1_dyn_p.m<br>README_MATLAB_DYNAMICS.txt
	example_optimization	b1_bounds.mat<br>b1_obj.m<br>b1_PE.m<br>cvodesg_b1.mexa64<br>cvodesg_b1.mexw32<br>README_MATLAB_OPTIM_EXAMPLE_B1.txt
SBML		b1.xml<br>README_SBML.txt

Table S3. Benchmark B2: File list

**Folder**	**Subfolder**	**FILES**
AMIGO		b2_amigo.m<br>pbest.mat<br>README_AMIGO_B2.txt
C		b2_fullC_template.c<br>compile_c.bat<br>fullC_data.mat<br>Makefile<br>nominalpars.h<br>README_C_B2.txt
COPASI		b2.cps<br>chass_exp_1.csv<br>chass_exp_2.csv<br>chass_exp_3.csv<br>chass_exp_4.csv<br>chass_exp_5.csv<br>chass_param.csv<br>README_COPASI_B2.txt
Matlab		b2.m<br>b2_obj.m<br>b2_pars.m<br>b2_test.m<br>radau5g_b2.mexa64<br>radau5g_b2.mexw32<br>README_MATLAB_B2.txt
	dynamics_in_Matlab	b2_dyn_p.m<br>README_MATLAB_DYNAMICS.txt
	example_optimization	b2_bounds.mat<br>b2_obj.m<br>b2_PE.m<br>radau5g_b2.mexa64<br>radau5g_b2.mexw32<br>README_MATLAB_OPTIM_EXAMPLE_B2.txt
SBML		b2.xml<br>README_SBML.txt

Table S4. Benchmark B3: File list

Folder	Subfolder	FILES
AMIGO		b3_amigo.m<br>b3_data.mat<br>b3_dynamics.m<br>b3_initial.m<br>b3_mex.mexa64<br>b3_mex.mexw32<br>pnom.mat<br>README_AMIGO_B3.txt
C		b3_fullC_template.c<br>compile_c.bat<br>fullC_data.mat<br>Makefile<br>nominalpars.h<br>README_C_B3.txt
COPASI		b3.cps<br>README_COPASI_B3.txt<br>xnom.csv
Matlab		b3.m<br>b3_data.mat<br>b3_dyn.m<br>b3_initial.m<br>b3_mex.mexa64<br>b3_mex.mexw32<br>b3_obj.m<br>b3_test.m<br>README_MATLAB_B3.txt
	dynamics_in_Matlab	b3_dyn_p.m<br>README_MATLAB_DYNAMICS.txt
	example_optimization	b3_bounds.mat<br>b3_data.mat<br>b3_dyn.m<br>b3_initial.m<br>b3_mex.mexa64<br>b3_mex.mexw32<br>b3_obj.m<br>b3_PE.m<br>README_MATLAB_OPTIM_EXAMPLE_B3.txt
SBML		b3.xml<br>README_SBML.txt

Table S5. Benchmark B4: File list

Folder	Subfolder	FILES
AMIGO		b4_amigo.m<br>b3_bounds.mat<br>pnom.mat<br>README_AMIGO_B4.txt
C		b4_fullC_template.c<br>compile_c.bat<br>fullC_data.mat<br>Makefile<br>nominalpars.h<br>README_C_B4.txt
COPASI		b4.cps<br>b4.csv<br>README_COPASI_B4.txt
Matlab		b4.m<br>b4_obj.m<br>b4_test.m<br>radau5g_b4_mex.mexa64<br>radau5g_b4_mex.mexw32<br>README_MATLAB_B4.txt
	dynamics_in_Matlab	b4_dyn_p.m<br>README_MATLAB_DYNAMICS.txt
	example_optimization	b4_bounds.mat<br>b4_obj.m<br>b4_PE.m<br>radau5g_b4_mex.mexa64<br>radau5g_b4_mex.mexw32<br>README_MATLAB_OPTIM_EXAMPLE_B4.txt
SBML		b4.xml<br>README_SBML.txt

Table S6. Benchmark B5: File list

Folder	Subfolder	FILES
AMIGO		b5_amigo.m<br>logic.c<br>pnom.mat<br>README_AMIGO_B5.txt
C		b5_fullC_template.c<br>compile_c.bat<br>fullC_data.mat<br>Makefile<br>nominalpars.h<br>README_C_B5.txt
Matlab		b5_data.mat<br>b5_obj.m<br>b5_test.m<br>cvodesg_b5.mexa64<br>cvodesg_b5.mexw32<br>README_MATLAB_B5.txt
	example_optimization	b5_bounds.mat<br>b5_data.mat<br>b5_obj.m<br>b5_PE.m<br>cvodesg_b5.mexa64<br>cvodesg_b5.mexw32<br>README_MATLAB_OPTIM_EXAMPLE_B5.txt
SBML-qual		B5.xml<br>README_SBML-qual.txt

Table S7. Benchmark B6: File list

Folder	Subfolder	FILES
AMIGO		b6.mexa64
		b6.mexglx
		b6_amigo.m
		b6_obj.m
		dm_hkgn53_wls_5_003
		pbest.mat
		README_AMIGO_B6.txt
C		dm_hkgn53_wls_5_003
		ggn
		ggn.c
		ggn.h
		ggn.o
		Makefile
		README.txt
	fly	...
	lib32	...
	lib64	...
	selected	...
	util	...
Matlab		b6.mexa64
		b6.mexglx
		b6_dyn.m
		b6_obj.m
		b6_test.m
		callunfold.m
		dm_hkgn53_wls_5_003
		README_MATLAB_B6.txt
		setParameters
		setParameters.m
		unfold
	example_optimization	b6.mexa64
		b6.mexglx
		b6_bounds.mat
		b6_obj.m
		b6_PE.m
		dm_hkgn53_wls_5_003
		README_MATLAB_OPTIM_EXAMPLE_B6.txt

List of files provided with the B6 benchmark. NOTE: additional files included in the auxiliary folders (fly, lib32, lib64, selected, util) are not listed here.

2 Requirements, installation, and instructions

2.1 COPASI

Download the COPASI software from `http://copasi.org/tiki-index.php?page=download`. We strongly recommend using the latest version. The benchmarks presented here have been tested with COPASI version 4.13 (Build 87). Note that using older versions may cause errors with some benchmarks.

Installation instructions: follow the instructions in:
`http://copasi.org/tiki-index.php?page=Installation&structure=DocumentationNew`.

Test the installation with the examples included in the COPASI distribution. Then test the benchmark models, e.g. B4:

1. Open the b4.cps file, either by double-clicking on it (which will automatically start COPASI) or by launching COPASI and going to the File→Open... tab.

2. Now the model is loaded in COPASI. You can browse the model details on the left side of the COPASI screen, in the "Model" menu. The different tasks are in the "Tasks" menu.

3. Run the "Time Course" task, which should result in an output like the one shown in Figure S1 (to view the plot, choose "Concentrations, Volumes, and Global Quantity Values" in the Output Assistant).

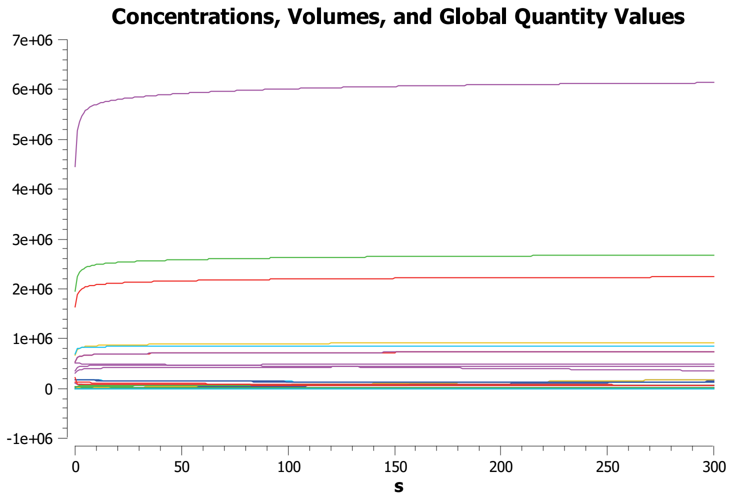

Figure S1. Benchmark 4. Copasi: time course. Plot of the result of the Time Course task in COPASI, with default settings.

To perform parameter estimation in COPASI, follow these steps:

1. Open the b*.cps file, either by double-clicking on it (which will automatically start COPASI) or by launching COPASI and going to the File→Open... tab.

2. Now the model is loaded in COPASI. You can browse the model details on the left side of the COPASI screen, in the "Model" menu. The different tasks are in the "Tasks" menu.

3. To perform parameter estimation, go to Tasks→Parameter Estimation.

4. Note that the model parameters have, by default, the nominal values. While this is fine for simulation purposes, in a typical parameter estimation benchmark problem the start values for the parameters (or "guesses") must be different from the nominal ones. Usually one would like the initial values to be random guesses taken from within reasonable bounds. To do this, check the "Randomize Start Values" box at the upper left part of the Parameter Estimation screen (just below the "Parameter Estimation" title in bold font). *This should be done for benchmarks B1, B3, and B4.* Note that benchmark B2 is an exception because it uses real data; in this case the nominal parameter values are the starting point of the optimization, and the goal of benchmark B2 is to find a parameter vector that improves the fit obtained by the nominal vector.

5. Before you can execute a parameter estimation task you need to specify the dataset which COPASI will use to fit the parameters you have specified. To choose an objective function, click on the "Experimental Data" button on the upper right corner of the screen, and select a weight method. Information about the different weight schemes is given in the following page of the COPASI manual: `http://www.copasi.org/tiki-index.php?page=OD.Experimental.Data&structure=OD`. Note that COPASI scales the weights so that for each experiment the maximal occurring weight is 1, which makes the definition of the objective functions different from the ones used in other formats.

6. Click the "Output Assistant" button in the lower right corner. This will open a window where you can select the type of plot(s) that will be generated to show the parameter estimation results.

7. Select the optimization method in the "Method" section (lower half of the screen), and change its default settings if desired.

8. Finally, click the "Run" button at the bottom of the screen to start the optimization.

2.2 Matlab

You will need Matlab R2008 or newer. If you want to use the pre-compiled mex-files provided, you will need to use either a 32 bit version (in Windows 7) or a 64 bit version (in Linux). Note that the 32 bit requirement applies to the Matlab version, not to the operating system (you can use 32 bit Matlab in a 64 bit OS).

The Matlab implementation includes a number of files. While the files list may vary slightly from one benchmark to the other, the core files are:

- b*_test.m carries out a test integration.
- b*.m returns the system derivatives (dxdt) for a particular parameter vector (p), states vector (x), and time instant (t). If no parameter or states vector is provided, it returns the nominal values of the parameters and the initial conditions for the states.
- b*_obj.m calculates the objective function used in the optimizations for a particular parameter vector. This function returns three outputs: the objective function itself, the constraints, and the residuals.
- b*_dyn.m integrates the system dynamics for a particular parameter vector, and returns the system outputs that will be used in the calculation of the objective function.

Before using the models in an optimization it is advisable to test that there are no compatibility issues with the files and that the results are reproducible. For the Matlab implementations–which can be used alone or in combination with the AMIGO toolbox– test files are provided, named *b ∗ _test.m* (*b1_test.m*, *b2_test.m*, ...). The Matlab implementation can be tested simply by running:

>> b*_test
This script, b*_test.m, integrates the b* model with nominal parameter values, calculates the objective function, and displays its value.

The Matlab scripts allow the user to set up a customized parameter estimation with any method implemented in Matlab. By selecting b*_obj.m as the objective function, new optimization methods can be tested and their results can be benchmarked against those obtained with AMIGO.

2.2.1 Pre-compiled (fixed) vs. editable dynamics in the Matlab implementations

For every benchmark the model dynamics are provided in pre-compiled mex-files (with extension .mexa64 for Linux 64 bit, .mexw32 for Windows 32 bit). These implementations are computationally faster than the standard Matlab code, hence their use is recommended.

Additionally, for benchmarks B1–B4 the complete dynamics are also provided in Matlab code, as m-files named "b*_dyn_p.m" included in a subfolder of the root Matlab folder named "dynamics_in_Matlab". They can be used for inspecting and changing the systems equations (only for advanced users). If you wish to do so, you must replace in the b*_obj.m files the calls to the mex-files by appropriate calls to the m-files.

Table S8. Mex-file implementations provided for use Matlab/AMIGO

	B1	B2	B3	B4	B5	B6
Linux 64 bit	*	*	*	*	*	*
Linux 32 bit	–	–	–	–	–	*
Matlab 32 bit	*	*	*	*	*	–

2.3 AMIGO

You will need Matlab R2008 or newer.

The latest version of AMIGO can be downloaded freely for non-commercial use from the CSIC webpage. However, please note that a specific version of AMIGO is provided with this collection of benchmarks, which contains some adjustments not available in other released versions. To avoid compatibility issues, **the AMIGO implementations of the benchmark problems should be used with the version of AMIGO provided with this benchmark, AMIGO2014bench**. This AMIGO version can be downloaded from `https://sites.google.com/site/biopredynbenchmarks/download`. Alternatively, it is also supplied as Additional Files 4 and 5 in the original publication, available at the BMC Systems Biology webpage. However, please note that the above link contains the most up-to-data version.

Installation instructions:

1. Unzip the .zip archive in your computer. IMPORTANT: to avoid errors, store the AMIGO folder in a directory without spaces (i.e., avoid routes such as C: Program Files AMIGO. Instead, use e.g. C: AMIGO)

2. Start a Matlab session and go to the AMIGO folder

3. Type:
>> AMIGO_Startup
every time you want to use the AMIGO toolbox

Please make sure AMIGO is properly installed (e.g. by running some of the examples included in the Appendix of AMIGO's user guide) before attempting to run the benchmark problems described here.

The AMIGO implementation of the benchmarks consists of a main file called b*_amigo.m and, in some cases, of some additional files. Using the AMIGO implementation is straightforward:

1. Copy the file(s) in the AMIGO folder
2. Start a Matlab session and go to the AMIGO folder
3. Initialize the AMIGO toolbox: run the script
   >> AMIGO_Startup
4. Prepare the model: run
   >> AMIGO_Prep('b*_amigo')
   This step only has to be performed the first time the model is used
5. Start an optimization: run
   >> AMIGO_PE('b*_amigo')
   This will carry out a parameter estimation task using the default options in AMIGO. If you want to customize the optimization, edit the corresponding options before this step. For example, if you are using eSS as the optimization method for parameter estimation (default), the options can be changed in the following file: Kernel/Opt_solvers/ess_options_defaults

To test the benchmarks, load and run benchmark B4 following the steps above. In the ess_options_defaults file, set:
opts.maxtime=3600;
opts.local.solver='fmincon';
opts.local.finish='fmincon';
This should result in a fit similar to the one shown on Figure S10 in this document.

Parameter estimation is just one of the tasks that can be carried out in AMIGO. The implementation provided can also be used for model simulation (AMIGO_SModel, AMIGO_SObs, AMIGO_SData), identifiability analysis (AMIGO_LRank), etc. To do this, replace the AMIGO_PE in step (5) with the corresponding method. Please note that, depending on the task, you may want to modify the parameter values too: in most cases, benchmark files are provided by default with random guesses for the parameter values; but to carry out e.g. local ranking of the parameters with LRank, the nominal (or an optimal) parameter vector should be used. This can be done by changing the values of inputs.model.par and inputs.PEsol.global_theta_guess in the AMIGO files.

2.4 C

Each C directory contains the following files:

- b*_fullC_template.c is a file that contains examples for some simple tasks that you can do using the benchmark, e.g. evaluate a parameter vector
- fullC_data.mat is a Matlab file containing the experimental design and data
- compile_c.bat is a Windows batch script with the GCC command to compile the C implementations
- Makefile is used for compiling the template file in Linux
- nominalpars.h is a header file that contains the nominal (or best known) parameter values
- README_C_*.txt contains instructions

Note that, due to the particularities of being a spatial model, the implementation of **benchmark B6** has some differences with that of the other benchmarks. If you wish to use the C implementation of B6, please refer to the detailed instructions provided in the corresponding README file. For benchmarks B1–B5, refer to the instructions provided in the subsections below (which can also be found in the corresponding README files).

2.4.1 Requirements and installation instructions for Windows

Requirements for Windows (32-bit and 64-bit):

- A 32-bit installation of Matlab or Matlab Compiler Runtime (MCR) is needed. MCR is available at `http://www.mathworks.es/products/compiler/mcr/`. Note that you can run the 32-bits libraries even if you have the 64-bit version of Windows
- GCC compiler; this distribution has been tested with the minGW version shipped with RTools: `http://cran.r-project.org/bin/windows/Rtools/` (tested with Rtools version 3.1.0.1939 and gcc-4.6.3)
- a specific version of AMIGO (AMIGO2014bench) must be used with this collection of benchmarks. This AMIGO version can be downloaded from `https://sites.google.com/site/biopredynbenchmarks/download`. Alternatively, it is also supplied as Additional Files 4 and 5 in the original publication, available at the BMC Systems Biology webpage. However, please note that the above link contains the most up-to-data version.

How to compile and run benchmarks B1–B5:

1. A compiled version of the libAMIGO library is needed. For Windows 32 bits the library is included in the AMIGO distribution, under:
   AMIGO2014bench/Kernel/libAMIGO/lib_win32/vs

   On Windows, the libAMIGO library should be on the system path, e.g.:
   C:/Desktop/AMIGO2014bench/Kernel/libAMIGO/lib_win32/vs

   How do I set my system path under Windows?
   `http://www.mathworks.es/support/solutions/en/data/1-15ZLK/`
2. Set the location of your Matlab installation in the first lines of the compile_c.bat file. Two different examples:

   a) A Matlab compiler installation
   set "MATLAB_PATH=C:/Program Files (x86)/MATLAB/MATLAB Compiler Runtime/v82"

   b) A MatlabR2011b version installed under C:/MATLAB
   set "MATLAB_PATH=C:/MATLAB/R2011b"
3. Set the location of your Matlab libraries:
   set "MATLAB_LIB=%MATLAB_PATH%/bin/win32"
4. Set the location of you AMIGO installation directory, e.g.:
   set "AMIGO_PATH=.../.../AMIGO2014bench"
5. Run the script compile_c.bat file in the Windows command line
6. Run the binary, e.g.: B1

2.4.2 Requirements and installation instructions for Linux and macOSx

Requirements for Linux (32-bit and 64-bit):

- An installation of Matlab or Matlab Compiler Runtime (MCR) is needed. MCR is available at `http://www.mathworks.es/products/compiler/mcr/`. Choose the MCR version appropriate for your machine(32-bit or 64-bit)
- GCC compiler; this distribution has been tested with the minGW version shipped with RTools: `http://cran.r-project.org/bin/windows/Rtools/` (tested with Rtools version 3.1.0.1939 and gcc-4.6.3)
- a specific version of AMIGO (AMIGO2014bench) must be used with this collection of benchmarks. This AMIGO version can be downloaded from `https://sites.google.com/site/biopredynbenchmarks/download`. Alternatively, it is also supplied as Additional Files 4 and 5 in the original publication, available at the BMC Systems Biology webpage. However, please note that the above link contains the most up-to-data version.

How to compile and run benchmarks B1–B5:

1. Make sure you have Matlab or Matlab compiler runtime installed
   (available at `http://www.mathworks.es/products/compiler/mcr/`)
2. Move into the the AMIGO directory:
   AMIGO2014bench
3. Execute the following steps (you can copy and paste after editing the paths):
   #This path requires edition. Introduce the location of your matlab installation.
   export MATLAB_PATH=/usr/local/matlab

   #If you have a 32 bits matlab installation choose glnx86 otherwise choose glnxa64
   #export MATLAB_LIB=$MATLAB_PATH/bin/glnxa64
   export MATLAB_LIB=$MATLAB_PATH/bin/glnx86

   #Change the AMIGO PATH to match the location of your AMIGO installation
   export AMIGO_PATH=$(pwd)

   Alternatively, you can modify the Makefile in order not to depend on the environment variables
4. Execute the compile_libAMIGO.sh script to compile the libAMIGO library. Make sure you have execution permission:
   chmod +x compile_libAMIGO.sh
   ./compile_libAMIGO.sh
5. Move to the C folder of the corresponding benchmark
6. Compile the the benchmark:
   make clean
   make
7. Execute the binary,e.g.:
   ./B1

2.4.3 Brief overview of the template file

In the b*_fullC_template.c file we provide the example code to perform a number of tasks:

1. How to evaluate an objective function, how_to_evaluate(AMIGO_problem* amigo_problem)
   We show the objective function value obtained with the nominal parameter vector (or with a good guess), and how to generate a new parameter vector and evaluate it

2. Implement your own objective function, objective_function(double* x, void* data)
   The objective function used in the previous example is the the squared difference between the data and the model simulation scaled by a factor, which in this case corresponds to the inverse of the expected experimental error. It is possible to use a different objective function if you prefer. To this end, we illustrate how the computations are done in the function:
   how_to_create_a_new_objective_function(AMIGO_problem* amigo_problem). By modifying it, an alternative objective function can be defined

3. Call an optimization algorithm, random_search(AMIGO_problem* amigo_problem)
   Each optimization solve in C is called in a specific way. However, it is common to provide an objective function that receives an array (double*) of values and a pointer that allows you to specify the location of some ad hoc data. To illustrate how an optimization algorithm can be configured, we provide a (pseudo) optimization algorithm, i.e. a random search, where parameter solutions are randomly generated and evaluated. Please not that random search is NOT a recommended optimization method, and it is provided here solely for demonstration purposes.

2.4.4 Brief Note on Compiler Versions

Minor differences related with numerical precision might arise depending on the the used compiler and optimization flags. On Windows we have tested the fullC benchmarks with gcc-4.6.3. The library libAMIGO provided for Windows 32-bits (note that it can be executed in a 64-bit OS) was compiled with Visual Studio 2010.

On Linux we have tested the fullC benchmarks with gcc-4.3.1 (64-bits).

3 Testing reproducibility

IMPORTANT: please note that, due to the use of different compilers, compiler options, platforms, and/or execution types, there may be small differences in the integrations, which will originate different objective function values. Some results are included in Table S10 below. The tolerances used for integrating the models in Matlab (with or without AMIGO) are shown in Table S9.

Table S9. Integrator tolerances set in Matlab for the different benchmarks

	B1	B2	B3	B4	B5	B6
Relative tolerance	10^{-4}	10^{-4}	10^{-6}	10^{-6}	10^{-6}	10^{-6}
Absolute tolerance	10^{-6}	10^{-6}	10^{-6}	10^{-6}	10^{-3}	10^{-6}

Table S10. Differences in objective function values with different solvers

**Model**	**Compiler**	**Opt. Flag**	**solver**	**execution**	**Matlab**	**LLK**
B1	VS	/Od	cvodes	standard	PCWIN	7.8177420E+05
B1	VS	/Od	cvodes	costMex	PCWIN	7.8177420E+05
B1	VS	/O1	cvodes	standard	PCWIN	7.8177420E+05
B1	VS	/O1	cvodes	costMex	PCWIN	7.8177420E+05
B1	VS	/O2	cvodes	standard	PCWIN	7.8177420E+05
B1	VS	/O2	cvodes	costMex	PCWIN	
B1	VS	/Ox	cvodes	standard	PCWIN	7.8177420E+05
B1	VS	/Ox	cvodes	costMex	PCWIN	
B1	gcc	O0	cvodes	standard	PCWIN	1.6011050E+06
B1	gcc	O0	cvodes	costMex	PCWIN	1.7985910E+06
B1	gcc	O1	cvodes	standard	PCWIN	1.0493670E+06
B1	gcc	O1	cvodes	costMex	PCWIN	9.7428280E+05
B1	gcc	O2	cvodes	standard	PCWIN	1.0493670E+06
B1	gcc	O2	cvodes	costMex	PCWIN	9.7428280E+05
B1	gcc	O3	cvodes	standard	PCWIN	1.0493670E+06
B1	gcc	O3	cvodes	costMex	PCWIN	9.7428280E+05
B1	gcc	O0	cvodes	fullC	PCWIN	4.9725670E+05
B1	gcc	O1	cvodes	fullC	PCWIN	1.6641370E+06
B1	gcc	O2	cvodes	fullC	PCWIN	3.8916910E+05
B1	gcc	O3	cvodes	fullC	PCWIN	3.8916910E+05
B1	gcc		cvodes	standard	GLXA64	1.0845720E+06
B1	gcc		cvodes	costMex	GLXA64	1.0845720E+06
B1	gcc		cvodes	MATLAB	GLXA64	1.0857000E+06
B1	gcc		cvodes	standard	GLNX86	6.5381130E+05
B1	gcc		cvodes	costMex	GLNX86	6.5381130E+05
B1	gcc		cvodes	MATLAB	GLNX86	6.5498000E+05
B1	gcc		cvodes	fullC	GLXA64	1.0845720E+06
B1	gcc		cvodes	fullC	GLNX86	
B2	NA	NA	ode15s	standard	PCWIN	3.2451160E+04
B2	g95	O0	radau5	standard	PCWIN	3.1096170E+04
B2	NA	NA	cvodes	MATLAB	PCWIN	3.1136000E+04
B2	VS	/Od	cvodes	standard	PCWIN	3.1138130E+04
B2	VS	/Od	cvodes	costMex	PCWIN	3.1138130E+00
B2	g95	O1	radau5	standard	PCWIN	3.1096170E+04
B2	VS	/O1	cvodes	standard	PCWIN	3.1138130E+04

B2	VS	/O1	cvodes	costMex	PCWIN	3.1138130E+04
B2	g95	O2	radau5	standard	PCWIN	3.1096170E+04
B2	VS	/O2	cvodes	standard	PCWIN	3.1138130E+04
B2	VS	/O2	cvodes	costMex	PCWIN	3.1138130E+04
B1	g95	O3	radau5	standard	PCWIN	3.1096170E+04
B2	VS	/Ox	cvodes	standard	PCWIN	3.1138130E+04
B2	VS	/Ox	cvodes	costMex	PCWIN	3.1138130E+04
B2	gcc	O0	cvodes	standard	PCWIN	3.1138130E+04
B2	gcc	O0	cvodes	costMex	PCWIN	3.1138130E+00
B2	gcc	O1	cvodes	standard	PCWIN	3.1138130E+04
B2	gcc	O1	cvodes	costMex	PCWIN	3.1138130E+04
B2	gcc	O2	cvodes	standard	PCWIN	3.1138130E+04
B2	gcc	O2	cvodes	costMex	PCWIN	3.1138130E+04
B2	gcc	O3	cvodes	standard	PCWIN	3.1138130E+04
B2	gcc	O3	cvodes	costMex	PCWIN	3.1138130E+04
B2	gcc	O0	cvodes	fullC	PCWIN	3.1132610E+04
B2	gcc	O1	cvodes	fullC	PCWIN	3.1132610E+04
B2	gcc	O2	cvodes	fullC	PCWIN	3.1132610E+04
B2	gcc	O3	cvodes	fullC	PCWIN	3.1132610E+04
B2	gcc		cvodes	standard	GLXA64	3.1138130E+04
B2	gcc		cvodes	costMex	GLXA64	3.1138130E+04
B2	gcc		cvodes	MATLAB	GLXA64	3.1136000E+04
B2	g95		radau5	standard	GLXA64	3.1096170E+04
B2	gcc		cvodes	standard	GLNX86	3.1138130E+04
B2	gcc		cvodes	costMex	GLNX86	3.1138130E+04
B2	gcc		cvodes	MATLAB	GLNX86	3.1136000E+04
B2	g95		radau5	standard	GLNX86	3.1096170E+04
B2	gcc		cvodes	fullC	GLXA64	3.1132610E+04
B2	gcc		cvodes	fullC	GLXA86	3.1132610E+04
B3	NA	NA	cvodes	MATLAB	PCWIN	4.9778000E-05
B3	VS	/Od	cvodes	costMex	PCWIN	2.5644680E-05
B3	VS	/O1	cvodes	costMex	PCWIN	2.5644680E-05
B3	VS	/O2	cvodes	costMex	PCWIN	2.5644680E-05
B3	VS	/Ox	cvodes	costMex	PCWIN	2.5644680E-05
B3	gcc	O0	cvodes	costMex	PCWIN	2.8044740E-05
B3	gcc	O1	cvodes	costMex	PCWIN	2.8044740E-05
B3	gcc	O2	cvodes	costMex	PCWIN	2.8044740E-05
B3	gcc	O3	cvodes	costMex	PCWIN	2.8044740E-05
B3	gcc	O0	cvodes	fullc	PCWIN	3.9159330E-05
B3	gcc	O1	cvodes	fullc	PCWIN	4.9993660E-05
B3	gcc	O2	cvodes	fullc	PCWIN	3.7396250E-05
B3	gcc	O3	cvodes	fullc	PCWIN	3.7396250E-05
B3	gcc		cvodes	costMex	GLXA64	2.7314070E-05
B3	gcc		cvodes	MATLAB	GLXA64	0.0000000E+00
B3	gcc		cvodes	costMex	GLNX86	2.9083310E-05
B3	gcc		cvodes	fullC	GLXA64	5.7404440E-05
B3	gcc		cvodes	fullC	GLNX86	5.1935180E-05
B4	NA	NA	ode15s	standard	PCWIN	3.9066890E+01
B4	NA	NA	radau5	MATLAB	PCWIN	3.9067350E+01
B4	g95	O0	radau5	standard	PCWIN	3.9066950E+01
B4	VS	/Od	cvodes	standard	PCWIN	3.9071020E+01
B4	VS	/Od	cvodes	costMex	PCWIN	3.9071020E+01
B4	g95	O1	radau5	standard	PCWIN	3.9067350E+01
B4	VS	/O1	cvodes	standard	PCWIN	3.9071020E+01

B4	VS	/O1	cvodes	costMex	PCWIN	3.9071020E+01
B4	g95	O1	radau5	standard	PCWIN	3.9066950E+01
B4	VS	/O1	cvodes	standard	PCWIN	3.9071020E+01
B4	VS	/O1	cvodes	costMex	PCWIN	3.9071020E+01
B4	g95	O3	radau5	standard	PCWIN	3.9066950E+01
B4	VS	/Ox	cvodes	standard	PCWIN	3.9071020E+01
B4	VS	/Ox	cvodes	costMex	PCWIN	3.9071020E+01
B4	gcc	O0	cvodes	standard	PCWIN	3.9071000E+01
B4	gcc	O0	cvodes	costMex	PCWIN	3.9071020E+01
B4	gcc	O1	cvodes	standard	PCWIN	3.9071000E+01
B4	gcc	O1	cvodes	costMex	PCWIN	3.9071020E+01
B4	gcc	O2	cvodes	standard	PCWIN	3.9071000E+01
B4	gcc	O2	cvodes	costMex	PCWIN	3.9071020E+01
B4	gcc	O3	cvodes	standard	PCWIN	3.9071000E+01
B4	gcc	O3	cvodes	costMex	PCWIN	3.9071020E+01
B4	gcc	O0	cvodes	fullc	PCWIN	3.9071010E+01
B4	gcc	O1	cvodes	fullc	PCWIN	3.9071030E+01
B4	gcc	O2	cvodes	fullc	PCWIN	3.9071020E+01
B4	gcc	O3	cvodes	fullc	PCWIN	3.9071020E+01
B4	gcc		cvodes	standard	GLXA64	3.9071030E+01
B4	gcc		cvodes	costMex	GLXA64	3.9071030E+01
B4	gcc		cvodes	MATLAB	GLXA64	3.9067350E+01
B4	NA	NA	ode15s	MATLAB	GLXA64	3.9066890E+01
B4	gcc		cvodes	standard	GLNX86	3.9071030E+01
B4	gcc		cvodes	costMex	GLNX86	3.9071030E+01
B4	gcc		cvodes	MATLAB	GLNX86	3.9067350E+01
B4	NA	NA	ode15s	MATLAB	GLNX86	3.9066890E+01
B4	g95		radau5	standard	GLNX86	3.9066950E+01
B4	gcc		cvodes	fullC	GLXA64	3.9071030E+01
B4	gcc		cvodes	fullC	GLNX86	3.9071030E+01
B5	VS	/Od	cvodes	standard	PCWIN	4.2726770E+03
B5	VS	/Od	cvodes	costMex	PCWIN	4.2726770E+03
B5	VS	/O1	cvodes	standard	PCWIN	4.2726770E+03
B5	VS	/O1	cvodes	costMex	PCWIN	4.2726770E+03
B5	VS	/O2	cvodes	standard	PCWIN	4.2726770E+03
B5	VS	/O2	cvodes	costMex	PCWIN	4.2726770E+03
B5	VS	/O3	cvodes	standard	PCWIN	4.2726770E+03
B5	VS	/O3	cvodes	costMex	PCWIN	4.2726770E+03
B5	gcc	O0	cvodes	standard	PCWIN	4.2726770E+03
B5	gcc	O0	cvodes	costMex	PCWIN	4.2726770E+03
B5	gcc	O1	cvodes	standard	PCWIN	4.2726770E+03
B5	gcc	O1	cvodes	costMex	PCWIN	4.2726770E+03
B5	gcc	O2	cvodes	standard	PCWIN	4.2726770E+03
B5	gcc	O2	cvodes	costMex	PCWIN	4.2726770E+03
B5	gcc	O3	cvodes	standard	PCWIN	4.2726770E+03
B5	gcc	O3	cvodes	costMex	PCWIN	4.2726770E+03
B5	gcc	O0	cvodes	fullc	PCWIN	4.2726770E+03
B5	gcc	O1	cvodes	fullc	PCWIN	4.2726770E+03
B5	gcc	O2	cvodes	fullc	PCWIN	4.2726770E+03
B5	gcc	O3	cvodes	fullc	PCWIN	4.2726770E+03
B5	gcc		cvodes	standard	GLXA64	4.2737460E+03
B5	gcc		cvodes	costMex	GLXA64	4.2737460E+03
B5	gcc		cvodes	MATLAB	GLXA64	4.2737000E+03
B5	gcc		cvodes	standard	GLNX86	4.2726770E+03

B5	gcc		cvodes	costMex	GLNX86	4.2726770E+03
B5	gcc		cvodes	fullC	GLXA64	4.2737460E+03
B5	gcc		cvodes	fullC	GLNX86	4.2726770E+03

4 Models: parameters, outputs, and data

4.1 B1: *S. cerevisiae*

Table S11 lists the model parameters and their nominal values. The measurable outputs of model B1 ("observables") consist of 38 metabolite concentrations (observables 1–38) and 6 fluxes (observables 39–44), which are listed in tables S12 and S13.

Table S11. Benchmark 1: parameter values

Param. name	Param. #	Param. value
Vmax_0001	p1	54.1365634735 mM·s^{-1}
Keq_0001	p2	10.54
Km0025_0001	p3	0.1 mM
Km0709_0001	p4	0.1 mM
Km0710_0001	p5	0.1 mM
Km1399_0001	p6	0.527 mM
Vmax_0004	p7	29.8936617862 mM·s^{-1}
Keq_0004	p8	10.54
Km0063_0004	p9	0.1 mM
Km0709_0004	p10	0.1 mM
Km0710_0004	p11	0.1 mM
Km1399_0004	p12	0.527 mM
Vmax_0005	p13	2.192738642 mM·s^{-1}
Keq_0005	p14	0.1207708779 mM
Km1543_0005	p15	0.467 mM
Km0002_0005	p16	0.1 mM
Km1538_0005	p17	0.282 mM
Vmax_0007	p18	0.0865554727 mM·s^{-1}
Keq_0007	p19	2
Km0077_0007	p20	0.1 mM
Km0312_0007	p21	0.1 mM
Vmax_0008	p22	0.016859852 mM·s^{-1}
Keq_0008	p23	2
Km0082_0008	p24	0.1 mM
Km0380_0008	p25	0.1 mM
Km0529_0008	p26	0.1 mM
Km1331_0008	p27	0.1 mM
Vmax_0012	p28	0.3473759638 mM·s^{-1}
Keq_0012	p29	11.6114349411
Km0991_0012	p30	0.298 mM
Km1203_0012	p31	0.0867 mM
Km0118_0012	p32	0.1 mM
Km1198_0012	p33	1.5 mM
Vmax_0014	p34	0.0017311095 mM·s^{-1}
Keq_0014	p35	0.2 mM
Km0142_0014	p36	0.1 mM
Km0313_0014	p37	0.1 mM
Km0419_0014	p38	0.1 mM
Vmax_0015	p39	0.0024235532 mM·s^{-1}
Keq_0015	p40	2
Km0141_0015	p41	0.1 mM
Km1212_0015	p42	0.1 mM
Km0142_0015	p43	0.1 mM

Km1207_0015	p44	0.1 mM
Vmax_0016	p45	0.4631679518 mM·s^{-1}
Keq_0016	p46	0.3795066414
Km0178_0016	p47	0.1 mM
Km1399_0016	p48	0.527 mM
Km0039_0016	p49	0.1 mM
Km0456_0016	p50	0.1 mM
Vmax_0018	p51	0.643588026 mM·s^{-1}
Keq_0018	p52	1.1744966443
Km0176_0018	p53	0.1 mM
Km0991_0018	p54	0.298 mM
Km0180_0018	p55	0.175 mM
Km0953_0018	p56	0.1 mM
Vmax_0020	p57	0.6422416075 mM·s^{-1}
Keq_0020	p58	1.3071895425
Km0551_0020	p59	0.1 mM
Km1360_0020	p60	0.153 mM
Km0349_0020	p61	0.1 mM
Km1322_0020	p62	0.1 mM
Vmax_0023	p63	0.2885182424 mM·s^{-1}
Keq_0023	p64	2
Km0162_0023	p65	0.1 mM
Km0165_0023	p66	0.1 mM
Vmax_0024	p67	0.6732092322 mM·s^{-1}
Keq_0024	p68	2
Km0232_0024	p69	0.1 mM
Km0373_0024	p70	0.1 mM
Km0162_0024	p71	0.1 mM
Km0529_0024	p72	0.1 mM
Vmax_0027	p73	0.2758234397 mM·s^{-1}
Keq_0027	p74	2
Km0835_0027	p75	0.1 mM
Km0454_0027	p76	0.1 mM
Vmax_0029	p77	0.4808637373 mM·s^{-1}
Keq_0029	p78	0.2 mM
Km0010_0029	p79	0.1 mM
Km0291_0029	p80	0.1 mM
Km0456_0029	p81	0.1 mM
Vmax_0032	p82	0.1786889648 mM·s^{-1}
Keq_0032	p83	0.88 mM
Km0390_0032	p84	0.1 mM
Km0423_0032	p85	0.44 mM
Km1322_0032	p86	0.1 mM
Vmax_0038	p87	0.0034622189 mM·s^{-1}
Keq_0038	p88	0.2 mM
Km0577_0038	p89	0.1 mM
Km0158_0038	p90	0.1 mM
Km0722_0038	p91	0.1 mM
Vmax_0039	p92	0.2752464032 mM·s^{-1}
Keq_0039	p93	2
Km0210_0039	p94	0.1 mM
Km0211_0039	p95	0.1 mM
Vmax_0040	p96	0.4587440054 mM·s^{-1}
Keq_0040	p97	0.2 mM

Km0349_0040	p98	0.1 mM
Km0210_0040	p99	0.1 mM
Km1322_0040	p100	0.1 mM
Vmax_0041	p101	0.0014504293 mM$\cdot$s^{-1}
Keq_0041	p102	2
Km0231_0041	p103	0.1 mM
Km1212_0041	p104	0.1 mM
Km1207_0041	p105	0.1 mM
Km1445_0041	p106	0.1 mM
Vmax_0060	p107	0.2885182424 mM$\cdot$s^{-1}
Keq_0060	p108	2
Km0165_0060	p109	0.1 mM
Km0009_0060	p110	0.1 mM
Vmax_0061	p111	0.6732092322 mM$\cdot$s^{-1}
Keq_0061	p112	0.1156
Km0009_0061	p113	0.1 mM
Km1198_0061	p114	1.5 mM
Km0010_0061	p115	0.1 mM
Km1203_0061	p116	0.0867 mM
Vmax_0065	p117	0.6422416075 mM$\cdot$s^{-1}
Keq_0065	p118	1.3071895425
Km0261_0065	p119	0.1 mM
Km1360_0065	p120	0.153 mM
Km0324_0065	p121	0.1 mM
Km1322_0065	p122	0.1 mM
Vmax_0079	p123	0.9637124801 mM$\cdot$s^{-1}
Keq_0079	p124	0.1792167832 mM
Km0301_0079	p125	0.1 mM
Km0434_0079	p126	4.29 mM
Km0999_0079	p127	0.1 mM
Km0302_0079	p128	0.1 mM
Km0394_0079	p129	1.29 mM
Km0991_0079	p130	0.298 mM
Km1322_0079	p131	0.1 mM
Vmax_0080	p132	0.1760765283 mM$\cdot$s^{-1}
Keq_0080	p133	2
Km0306_0080	p134	0.1 mM
Km1212_0080	p135	0.1 mM
Km0322_0080	p136	0.1 mM
Km1207_0080	p137	0.1 mM
Vmax_0091	p138	5.910259852 mM$\cdot$s^{-1}
Keq_0091	p139	2
Km0335_0091	p140	0.1 mM
Km0340_0091	p141	0.1 mM
Vmax_0096	p142	1.3652683229 mM$\cdot$s^{-1}
Keq_0096	p143	2
Km0146_0096	p144	0.1 mM
Km1212_0096	p145	0.1 mM
Km0016_0096	p146	0.1 mM
Km1207_0096	p147	0.1 mM
Vmax_0097	p148	1.3652683229 mM$\cdot$s^{-1}
Keq_0097	p149	0.0720126454
Km1399_0097	p150	0.527 mM
Km0146_0097	p151	0.1 mM

Km0456_0097	p152	0.1 mM
Vmax_0103	p153	0.1106755978 mM·s^{-1}
Keq_0103	p154	2
Km0373_0103	p155	0.1 mM
Km0367_0103	p156	0.1 mM
Km0529_0103	p157	0.1 mM
Vmax_0108	p158	0.9003803216 mM·s^{-1}
Keq_0108	p159	0.6013986014
Km0373_0108	p160	0.1 mM
Km0434_0108	p161	4.29 mM
Km0445_0108	p162	0.1 mM
Km0394_0108	p163	1.29 mM
Km1101_0108	p164	0.1 mM
Km1322_0108	p165	0.1 mM
Vmax_0111	p166	0.141738392 mM·s^{-1}
Keq_0111	p167	111.6 mM
Km0373_0111	p168	0.1 mM
Km0362_0111	p169	55.8 mM
Km0529_0111	p170	0.1 mM
Vmax_0115	p171	0.3662258223 mM·s^{-1}
Keq_0115	p172	0.6013986014
Km0434_0115	p173	4.29 mM
Km1192_0115	p174	0.1 mM
Km0394_0115	p175	1.29 mM
Km1191_0115	p176	0.1 mM
Vmax_0118	p177	0.3662258223 mM·s^{-1}
Keq_0118	p178	1.1744966443
Km0145_0118	p179	0.1 mM
Km0991_0118	p180	0.298 mM
Km0180_0118	p181	0.175 mM
Km1182_0118	p182	0.1 mM
Vmax_0142	p183	0.0414346818 mM·s^{-1}
Keq_0142	p184	2.6461538462
Km0386_0142	p185	0.1 mM
Km0434_0142	p186	4.29 mM
Km0394_0142	p187	1.29 mM
Km0423_0142	p188	0.44 mM
Vmax_0144	p189	0.0295962013 mM·s^{-1}
Keq_0144	p190	0.2 mM
Km1413_0144	p191	0.1 mM
Km0386_0144	p192	0.1 mM
Km1012_0144	p193	0.1 mM
Vmax_0148	p194	2.6350043346 mM·s^{-1}
Keq_0148	p195	1.7631913541
Km0423_0148	p196	0.44 mM
Km0434_0148	p197	4.29 mM
Km0394_0148	p198	1.29 mM
Vmax_0151	p199	0.2095027131 mM·s^{-1}
Keq_0151	p200	0.2 mM
Km0299_0151	p201	0.1 mM
Km0403_0151	p202	0.1 mM
Km0725_0151	p203	0.1 mM
Vmax_0152	p204	0.2492605269 mM·s^{-1}
Keq_0152	p205	0.88 mM

Km0393_0152	p206	0.1 mM
Km0423_0152	p207	0.44 mM
Km0725_0152	p208	0.1 mM
Vmax_0153	p209	0.7477815806 mM·s^{-1}
Keq_0153	p210	0.1266223488
Km0785_0153	p211	0.975 mM
Km0849_0153	p212	0.162 mM
Km0973_0153	p213	0.1 mM
Km0393_0153	p214	0.1 mM
Km0739_0153	p215	0.1 mM
Km1322_0153	p216	0.1 mM
Vmax_0154	p217	0.2501645507 mM·s^{-1}
Keq_0154	p218	0.6013986014
Km0298_0154	p219	0.1 mM
Km0434_0154	p220	4.29 mM
Km0201_0154	p221	0.1 mM
Km0394_0154	p222	1.29 mM
Vmax_0165	p223	63.8399999998 mM·s^{-1}
Keq_0165	p224	10788.8495781657
Km0359_0165	p225	0.178 mM
Km1203_0165	p226	0.0867 mM
Km0680_0165	p227	55.5 mM
Km1198_0165	p228	1.5 mM
Vmax_0173	p229	0.2561951009 mM·s^{-1}
Keq_0173	p230	626.9662921348
Km0359_0173	p231	0.178 mM
Km1207_0173	p232	0.1 mM
Km0362_0173	p233	55.8 mM
Km1212_0173	p234	0.1 mM
Vmax_0195	p235	0.1719323841 mM·s^{-1}
Keq_0195	p236	0.1251511689
Km0568_0195	p237	0.193 mM
Km1543_0195	p238	0.467 mM
Km0409_0195	p239	0.02 mM
Km1538_0195	p240	0.282 mM
Vmax_0202	p241	0.4295079449 mM·s^{-1}
Keq_0202	p242	2
Km0427_0202	p243	0.1 mM
Km1386_0202	p244	0.1 mM
Km0633_0202	p245	0.1 mM
Km1187_0202	p246	0.1 mM
Vmax_0203	p247	0.1184848249 mM·s^{-1}
Keq_0203	p248	3.14092 mM
Km0515_0203	p249	0.1 mM
Km0999_0203	p250	0.1 mM
Km0427_0203	p251	0.1 mM
Km0991_0203	p252	0.298 mM
Km1399_0203	p253	0.527 mM
Vmax_0207	p254	0.2615898731 mM·s^{-1}
Keq_0207	p255	0.2 mM
Km0015_0207	p256	0.1 mM
Km0725_0207	p257	0.1 mM
Km0965_0207	p258	0.1 mM
Vmax_0208	p259	0.7847696192 mM·s^{-1}

Keq_0208	p260	0.2051282051
Km0434_0208	p261	4.29 mM
Km0973_0208	p262	0.1 mM
Km0979_0208	p263	0.1 mM
Km0015_0208	p264	0.1 mM
Km0423_0208	p265	0.44 mM
Km0633_0208	p266	0.1 mM
Vmax_0211	p267	1.5218375558 mM·s^{-1}
Keq_0211	p268	0.0611282051 mM
Km0434_0211	p269	4.29 mM
Km0973_0211	p270	0.1 mM
Km0999_0211	p271	0.1 mM
Km0423_0211	p272	0.44 mM
Km0633_0211	p273	0.1 mM
Km0969_0211	p274	0.1 mM
Km0991_0211	p275	0.298 mM
Vmax_0214	p276	0.331272799 mM·s^{-1}
Keq_0214	p277	2
Km0455_0214	p278	0.1 mM
Km0973_0214	p279	0.1 mM
Km1194_0214	p280	0.1 mM
Km1322_0214	p281	0.1 mM
Vmax_0215	p282	1.1282986732 mM·s^{-1}
Keq_0215	p283	0.6013986014
Km0434_0215	p284	4.29 mM
Km0973_0215	p285	0.1 mM
Km0295_0215	p286	0.1 mM
Km0394_0215	p287	1.29 mM
Vmax_0216	p288	3.3944017338 mM·s^{-1}
Keq_0216	p289	1.1744966443
Km0991_0216	p290	0.298 mM
Km1271_0216	p291	0.1 mM
Km0180_0216	p292	0.175 mM
Km0973_0216	p293	0.1 mM
Vmax_0219	p294	1.7730407721 mM·s^{-1}
Keq_0219	p295	0.2 mM
Km0295_0219	p296	0.1 mM
Km1212_0219	p297	0.1 mM
Km0978_0219	p298	0.1 mM
Km1207_0219	p299	0.1 mM
Km1322_0219	p300	0.1 mM
Vmax_0225	p301	0.2019627697 mM·s^{-1}
Keq_0225	p302	0.0466200466
Km0434_0225	p303	4.29 mM
Km1386_0225	p304	0.1 mM
Km0326_0225	p305	0.1 mM
Km0633_0225	p306	0.1 mM
Vmax_0226	p307	139.4439156137 mM·s^{-1}
Keq_0226	p308	66.511627907 mM^{-1}
Km0394_0226	p309	1.29 mM
Km1322_0226	p310	0.1 mM
Km0434_0226	p311	4.29 mM
Vmax_0231	p312	0.0183597622 mM·s^{-1}
Keq_0231	p313	2

Km0262_0231	p314	0.1 mM
Km1212_0231	p315	0.1 mM
Km0122_0231	p316	0.1 mM
Km1207_0231	p317	0.1 mM
Vmax_0233	p318	0.027661975 mM·s^{-1}
Keq_0233	p319	20 mM^{-1}
Km0664_0233	p320	0.1 mM
Km1212_0233	p321	0.1 mM
Km1275_0233	p322	0.1 mM
Km0662_0233	p323	0.1 mM
Km1207_0233	p324	0.1 mM
Vmax_0234	p325	0.0286140852 mM·s^{-1}
Keq_0234	p326	0.2 mM
Km1207_0234	p327	0.1 mM
Km1578_0234	p328	0.1 mM
Km0456_0234	p329	0.1 mM
Km1212_0234	p330	0.1 mM
Km1579_0234	p331	0.1 mM
Vmax_0235	p332	0.0286140852 mM·s^{-1}
Keq_0235	p333	0.01156 mM
Km0297_0235	p334	0.1 mM
Km1198_0235	p335	1.5 mM
Km0209_0235	p336	0.1 mM
Km0456_0235	p337	0.1 mM
Km1203_0235	p338	0.0867 mM
Vmax_0236	p339	0.0182089633 mM·s^{-1}
Keq_0236	p340	2
Km0209_0236	p341	0.1 mM
Km1212_0236	p342	0.1 mM
Km0296_0236	p343	0.1 mM
Km1207_0236	p344	0.1 mM
Vmax_0237	p345	0.0182089633 mM·s^{-1}
Keq_0237	p346	2
Km1212_0237	p347	0.1 mM
Km1579_0237	p348	0.1 mM
Km1207_0237	p349	0.1 mM
Km1569_0237	p350	0.1 mM
Vmax_0238	p351	0.0286140852 mM·s^{-1}
Keq_0238	p352	20 mM^{-1}
Km0296_0238	p353	0.1 mM
Km1212_0238	p354	0.1 mM
Km1275_0238	p355	0.1 mM
Km1207_0238	p356	0.1 mM
Km1576_0238	p357	0.1 mM
Vmax_0239	p358	0.0286140852 mM·s^{-1}
Keq_0239	p359	20 mM^{-1}
Km1212_0239	p360	0.1 mM
Km1275_0239	p361	0.1 mM
Km1576_0239	p362	0.1 mM
Km1207_0239	p363	0.1 mM
Km1577_0239	p364	0.1 mM
Vmax_0240	p365	0.0286140852 mM·s^{-1}
Keq_0240	p366	20 mM^{-1}
Km1212_0240	p367	0.1 mM

Km1275_0240	p368	0.1 mM
Km1577_0240	p369	0.1 mM
Km1207_0240	p370	0.1 mM
Km1578_0240	p371	0.1 mM
Vmax_0241	p372	0.3719831077 mM·s^{-1}
Keq_0241	p373	2000 mM^{-1}_cubed
Km0122_0241	p374	0.1 mM
Km1212_0241	p375	0.1 mM
Km1275_0241	p376	0.1 mM
Km0297_0241	p377	0.1 mM
Km1207_0241	p378	0.1 mM
Vmax_0242	p379	0.027661975 mM·s^{-1}
Keq_0242	p380	20 mM^{-1}
Km0657_0242	p381	0.1 mM
Km1212_0242	p382	0.1 mM
Km1275_0242	p383	0.1 mM
Km0664_0242	p384	0.1 mM
Km1207_0242	p385	0.1 mM
Vmax_0243	p386	0.007654966 mM·s^{-1}
Keq_0243	p387	2
Km0700_0243	p388	0.1 mM
Km0657_0243	p389	0.1 mM
Vmax_0244	p390	0.0172664704 mM·s^{-1}
Keq_0244	p391	2
Km0662_0244	p392	0.1 mM
Km1212_0244	p393	0.1 mM
Km0666_0244	p394	0.1 mM
Km1207_0244	p395	0.1 mM
Vmax_0250	p396	4.6832050287 mM·s^{-1}
Keq_0250	p397	0.0538903614 mM
Km0434_0250	p398	4.29 mM
Km0445_0250	p399	0.1 mM
Km0999_0250	p400	0.1 mM
Km0394_0250	p401	1.29 mM
Km0455_0250	p402	0.1 mM
Km0991_0250	p403	0.298 mM
Km1322_0250	p404	0.1 mM
Vmax_0257	p405	0.0162071757 mM·s^{-1}
Keq_0257	p406	2
Km0539_0257	p407	0.1 mM
Km1331_0257	p408	0.1 mM
Km0471_0257	p409	0.1 mM
Km0633_0257	p410	0.1 mM
Vmax_0259	p411	0.001139623 mM·s^{-1}
Keq_0259	p412	20 mM^{-1}
Km0475_0259	p413	0.1 mM
Km1212_0259	p414	0.1 mM
Km1275_0259	p415	0.1 mM
Km0481_0259	p416	0.1 mM
Km1207_0259	p417	0.1 mM
Vmax_0267	p418	0.002279246 mM·s^{-1}
Keq_0267	p419	20 mM^{-1}
Km0481_0267	p420	0.1 mM
Km1212_0267	p421	0.1 mM

Km1275_0267	p422	0.1 mM
Km0493_0267	p423	0.1 mM
Km1207_0267	p424	0.1 mM
Vmax_0269	p425	0.002279246 mM·s^{-1}
Keq_0269	p426	20 mM^{-1}
Km0493_0269	p427	0.1 mM
Km1212_0269	p428	0.1 mM
Km1275_0269	p429	0.1 mM
Km0499_0269	p430	0.1 mM
Km1207_0269	p431	0.1 mM
Vmax_0278	p432	0.2429323601 mM·s^{-1}
Keq_0278	p433	2
Km0515_0278	p434	0.1 mM
Km1377_0278	p435	0.1 mM
Vmax_0279	p436	0.4587440054 mM·s^{-1}
Keq_0279	p437	0.2 mM
Km0324_0279	p438	0.1 mM
Km0515_0279	p439	0.1 mM
Km1322_0279	p440	0.1 mM
Vmax_0280	p441	1.2002358883 mM·s^{-1}
Keq_0280	p442	2
Km0516_0280	p443	0.1 mM
Km0940_0280	p444	0.1 mM
Vmax_0300	p445	2.8005504059 mM·s^{-1}
Keq_0300	p446	2
Km0373_0300	p447	0.1 mM
Km1271_0300	p448	0.1 mM
Km0522_0300	p449	0.1 mM
Km0529_0300	p450	0.1 mM
Vmax_0302	p451	1.2002358883 mM·s^{-1}
Keq_0302	p452	2
Km0522_0302	p453	0.1 mM
Km0516_0302	p454	0.1 mM
Vmax_0307	p455	0.2955292357 mM·s^{-1}
Keq_0307	p456	0.0926654239
Km0419_0307	p457	0.1 mM
Km0434_0307	p458	4.29 mM
Km1559_0307	p459	0.649 mM
Km0394_0307	p460	1.29 mM
Km0539_0307	p461	0.1 mM
Km1322_0307	p462	0.1 mM
Vmax_0309	p463	0.0412581087 mM·s^{-1}
Keq_0309	p464	10 mM^{-1}
Km1012_0309	p465	0.1 mM
Km1039_0309	p466	0.2 mM
Km0980_0309	p467	0.1 mM
Vmax_0310	p468	0.5955016523 mM·s^{-1}
Keq_0310	p469	0.02 mM_squared
Km0980_0310	p470	0.1 mM
Km0178_0310	p471	0.1 mM
Km0419_0310	p472	0.1 mM
Km0981_0310	p473	0.1 mM
Vmax_0311	p474	0.4054065996 mM·s^{-1}
Keq_0311	p475	1116

Km0981_0311	p476	0.1 mM
Km1233_0311	p477	0.1 mM
Km0362_0311	p478	55.8 mM
Km0980_0311	p479	0.1 mM
Vmax_0312	p480	0.0577613521 mM·s^{-1}
Keq_0312	p481	1116
Km0841_0312	p482	0.1 mM
Km1234_0312	p483	0.1 mM
Km0362_0312	p484	55.8 mM
Km0981_0312	p485	0.1 mM
Vmax_0317	p486	0.4170288838 mM·s^{-1}
Keq_0317	p487	200 mM^{-1}_squared
Km1059_0317	p488	0.1 mM
Km1212_0317	p489	0.1 mM
Km1275_0317	p490	0.1 mM
Km0262_0317	p491	0.1 mM
Km0722_0317	p492	0.1 mM
Km1207_0317	p493	0.1 mM
Vmax_0326	p494	0.0023369978 mM·s^{-1}
Keq_0326	p495	20 mM^{-1}
Km0419_0326	p496	0.1 mM
Km0654_0326	p497	0.1 mM
Km0589_0326	p498	0.1 mM
Vmax_0330	p499	0.0065435937 mM·s^{-1}
Keq_0330	p500	6.6511627907
Km0394_0330	p501	1.29 mM
Km0613_0330	p502	0.1 mM
Km0434_0330	p503	4.29 mM
Km0615_0330	p504	0.1 mM
Vmax_0336	p505	0.033719704 mM·s^{-1}
Keq_0336	p506	2
Km0529_0336	p507	0.1 mM
Km1524_0336	p508	0.1 mM
Km0380_0336	p509	0.1 mM
Km0619_0336	p510	0.1 mM
Vmax_0337	p511	0.0004661974 mM·s^{-1}
Keq_0337	p512	0.2 mM
Km1331_0337	p513	0.1 mM
Km0619_0337	p514	0.1 mM
Km1322_0337	p515	0.1 mM
Vmax_0339	p516	0.331272799 mM·s^{-1}
Keq_0339	p517	2
Km0061_0339	p518	0.1 mM
Km1275_0339	p519	0.1 mM
Km0837_0339	p520	0.1 mM
Km1269_0339	p521	0.1 mM
Vmax_0340	p522	0.0005180105 mM·s^{-1}
Keq_0340	p523	20 mM^{-1}
Km1084_0340	p524	0.1 mM
Km1445_0340	p525	0.1 mM
Km0475_0340	p526	0.1 mM
Vmax_0344	p527	0.0096672846 mM·s^{-1}
Keq_0344	p528	2
Km0625_0344	p529	0.1 mM

Km1212_0344	p530	0.1 mM
Km1207_0344	p531	0.1 mM
Km1487_0344	p532	0.1 mM
Vmax_0349	p533	0.1419740567 mM·s^{-1}
Keq_0349	p534	2
Km1194_0349	p535	0.1 mM
Km0061_0349	p536	0.1 mM
Vmax_0352	p537	0.5851149955 mM·s^{-1}
Keq_0352	p538	2
Km0016_0352	p539	0.1 mM
Km0232_0352	p540	0.1 mM
Vmax_0353	p541	0.1985005508 mM·s^{-1}
Keq_0353	p542	2
Km0008_0353	p543	0.1 mM
Km0056_0353	p544	0.1 mM
Vmax_0355	p545	0.0368918659 mM·s^{-1}
Keq_0355	p546	2
Km0943_0355	p547	0.1 mM
Km1376_0355	p548	0.1 mM
Km0633_0355	p549	0.1 mM
Km0745_0355	p550	0.1 mM
Vmax_0361	p551	2.2108191185 mM·s^{-1}
Keq_0361	p552	2
Km0645_0361	p553	0.1 mM
Km0743_0361	p554	0.1 mM
Km0644_0361	p555	0.1 mM
Km0739_0361	p556	0.1 mM
Vmax_0362	p557	1.5791565132 mM·s^{-1}
Keq_0362	p558	0.2 mM
Km0644_0362	p559	0.1 mM
Km0645_0362	p560	0.1 mM
Km1107_0362	p561	0.1 mM
Vmax_0364	p562	0.009242201 mM·s^{-1}
Keq_0364	p563	0.2 mM
Km0656_0364	p564	0.1 mM
Km0633_0364	p565	0.1 mM
Km0654_0364	p566	0.1 mM
Vmax_0366	p567	17.5300909501 mM·s^{-1}
Keq_0366	p568	18.1065088757
Km0188_0366	p569	0.0169 mM
Km1360_0366	p570	0.153 mM
Vmax_0386	p571	0.0048692984 mM·s^{-1}
Keq_0386	p572	0.2 mM
Km0595_0386	p573	0.1 mM
Km1101_0386	p574	0.1 mM
Km1212_0386	p575	0.1 mM
Km0456_0386	p576	0.1 mM
Km0529_0386	p577	0.1 mM
Km1065_0386	p578	0.1 mM
Km1207_0386	p579	0.1 mM
Vmax_0387	p580	0.0048692983 mM·s^{-1}
Keq_0387	p581	0.2 mM
Km1065_0387	p582	0.1 mM
Km1101_0387	p583	0.1 mM

Km1212_0387	p584	0.1 mM
Km0456_0387	p585	0.1 mM
Km0529_0387	p586	0.1 mM
Km1161_0387	p587	0.1 mM
Km1207_0387	p588	0.1 mM
Vmax_0389	p589	0.0048692983 mM·s^{-1}
Keq_0389	p590	0.2 mM
Km1101_0389	p591	0.1 mM
Km1161_0389	p592	0.1 mM
Km1212_0389	p593	0.1 mM
Km0456_0389	p594	0.1 mM
Km0529_0389	p595	0.1 mM
Km1207_0389	p596	0.1 mM
Km1286_0389	p597	0.1 mM
Vmax_0391	p598	0.0048692983 mM·s^{-1}
Keq_0391	p599	0.2 mM
Km1101_0391	p600	0.1 mM
Km1212_0391	p601	0.1 mM
Km1286_0391	p602	0.1 mM
Km0456_0391	p603	0.1 mM
Km0529_0391	p604	0.1 mM
Km1207_0391	p605	0.1 mM
Km1449_0391	p606	0.1 mM
Vmax_0393	p607	1.9093865602 mM·s^{-1}
Keq_0393	p608	0.002 mM_cubed
Km1101_0393	p609	0.1 mM
Km1212_0393	p610	0.1 mM
Km1449_0393	p611	0.1 mM
Km0456_0393	p612	0.1 mM
Km0529_0393	p613	0.1 mM
Km1084_0393	p614	0.1 mM
Km1207_0393	p615	0.1 mM
Vmax_0397	p616	0.6809514269 mM·s^{-1}
Keq_0397	p617	0.2 mM
Km1101_0397	p618	0.1 mM
Km1212_0397	p619	0.1 mM
Km1255_0397	p620	0.1 mM
Km0456_0397	p621	0.1 mM
Km0529_0397	p622	0.1 mM
Km0602_0397	p623	0.1 mM
Km1207_0397	p624	0.1 mM
Vmax_0398	p625	133.5099446538 mM·s^{-1}
Keq_0398	p626	0.002 mM_cubed
Km0373_0398	p627	0.1 mM
Km1101_0398	p628	0.1 mM
Km1212_0398	p629	0.1 mM
Km0456_0398	p630	0.1 mM
Km0529_0398	p631	0.1 mM
Km1207_0398	p632	0.1 mM
Km1255_0398	p633	0.1 mM
Vmax_0399	p634	0.2126628293 mM·s^{-1}
Keq_0399	p635	19.5
Km0423_0399	p636	0.44 mM
Km0602_0399	p637	0.1 mM

Km0633_0399	p638	0.1 mM
Km0434_0399	p639	4.29 mM
Km0529_0399	p640	0.1 mM
Km0595_0399	p641	0.1 mM
Vmax_0407	p642	0.0015540314 mM·s^{-1}
Keq_0407	p643	19.5
Km0423_0407	p644	0.44 mM
Km0633_0407	p645	0.1 mM
Km1454_0407	p646	0.1 mM
Km0434_0407	p647	4.29 mM
Km0529_0407	p648	0.1 mM
Km1449_0407	p649	0.1 mM
Vmax_0432	p650	0.014607895 mM·s^{-1}
Keq_0432	p651	0.2 mM
Km0602_0432	p652	0.1 mM
Km1101_0432	p653	0.1 mM
Km1212_0432	p654	0.1 mM
Km0456_0432	p655	0.1 mM
Km0529_0432	p656	0.1 mM
Km1073_0432	p657	0.1 mM
Km1207_0432	p658	0.1 mM
Vmax_0433	p659	0.014607895 mM·s^{-1}
Keq_0433	p660	0.2 mM
Km1073_0433	p661	0.1 mM
Km1101_0433	p662	0.1 mM
Km1212_0433	p663	0.1 mM
Km0456_0433	p664	0.1 mM
Km0529_0433	p665	0.1 mM
Km1176_0433	p666	0.1 mM
Km1207_0433	p667	0.1 mM
Vmax_0434	p668	0.014607895 mM·s^{-1}
Keq_0434	p669	0.2 mM
Km1101_0434	p670	0.1 mM
Km1176_0434	p671	0.1 mM
Km1212_0434	p672	0.1 mM
Km0456_0434	p673	0.1 mM
Km0529_0434	p674	0.1 mM
Km1207_0434	p675	0.1 mM
Km1302_0434	p676	0.1 mM
Vmax_0435	p677	0.0048692983 mM·s^{-1}
Keq_0435	p678	0.2 mM
Km1101_0435	p679	0.1 mM
Km1212_0435	p680	0.1 mM
Km1302_0435	p681	0.1 mM
Km0456_0435	p682	0.1 mM
Km0529_0435	p683	0.1 mM
Km1207_0435	p684	0.1 mM
Km1454_0435	p685	0.1 mM
Vmax_0438	p686	133.1333439792 mM·s^{-1}
Keq_0438	p687	20 mM^{-1}
Km0710_0438	p688	0.1 mM
Km1275_0438	p689	0.1 mM
Km0709_0438	p690	0.1 mM
Vmax_0439	p691	0.9485049398 mM·s^{-1}

Keq_0439	p692	2
Km0709_0439	p693	0.1 mM
Km1535_0439	p694	0.1 mM
Km0710_0439	p695	0.1 mM
Km1537_0439	p696	0.1 mM
Vmax_0445	p697	0.5739005538 mM·s^{-1}
Keq_0445	p698	0.1156
Km0722_0445	p699	0.1 mM
Km1198_0445	p700	1.5 mM
Km0456_0445	p701	0.1 mM
Km1203_0445	p702	0.0867 mM
Vmax_0446	p703	0.4160606166 mM·s^{-1}
Keq_0446	p704	6.6511627907
Km0120_0446	p705	0.1 mM
Km0394_0446	p706	1.29 mM
Km1322_0446	p707	0.1 mM
Km0434_0446	p708	4.29 mM
Km0722_0446	p709	0.1 mM
Km1487_0446	p710	0.1 mM
Vmax_0450	p711	25.0072374427 mM·s^{-1}
Keq_0450	p712	0.0398434783 mM
Km0555_0450	p713	1.15 mM
Km0629_0450	p714	0.29 mM
Km0764_0450	p715	0.079 mM
Vmax_0451	p716	0.4322118678 mM·s^{-1}
Keq_0451	p717	2
Km0725_0451	p718	0.1 mM
Km0066_0451	p719	0.1 mM
Vmax_0462	p720	0.0368918659 mM·s^{-1}
Keq_0462	p721	2
Km0745_0462	p722	0.1 mM
Km0943_0462	p723	0.1 mM
Km0190_0462	p724	0.1 mM
Km0633_0462	p725	0.1 mM
Vmax_0466	p726	13.7906063214 mM·s^{-1}
Keq_0466	p727	1.0362694301
Km0568_0466	p728	0.193 mM
Km1207_0466	p729	0.1 mM
Km0335_0466	p730	0.1 mM
Km1212_0466	p731	0.1 mM
Vmax_0467	p732	14.3453751324 mM·s^{-1}
Keq_0467	p733	0.6103626943
Km0568_0467	p734	0.193 mM
Km0557_0467	p735	0.0589 mM
Vmax_0470	p736	10.0719265766 mM·s^{-1}
Keq_0470	p737	589.2239248641 mM^{-1}
Km0180_0470	p738	0.175 mM
Km0419_0470	p739	0.1 mM
Km1203_0470	p740	0.0867 mM
Km0991_0470	p741	0.298 mM
Km1198_0470	p742	1.5 mM
Vmax_0471	p743	10.0719265766 mM·s^{-1}
Keq_0471	p744	34.0571428571 mM^{-1}
Km0180_0471	p745	0.175 mM

Km0419_0471	p746	0.1 mM
Km1212_0471	p747	0.1 mM
Km0991_0471	p748	0.298 mM
Km1207_0471	p749	0.1 mM
Vmax_0476	p750	6.1984682154 mM·s^{-1}
Keq_0476	p751	0.2018116112
Km0419_0476	p752	0.1 mM
Km0434_0476	p753	4.29 mM
Km0991_0476	p754	0.298 mM
Km0394_0476	p755	1.29 mM
Km0999_0476	p756	0.1 mM
Km1322_0476	p757	0.1 mM
Vmax_0481	p758	0.2602857706 mM·s^{-1}
Keq_0481	p759	0.2 mM
Km0754_0481	p760	0.1 mM
Km1212_0481	p761	0.1 mM
Km0750_0481	p762	0.1 mM
Km1207_0481	p763	0.1 mM
Vmax_0483	p764	0.2129610851 mM·s^{-1}
Keq_0483	p765	200 mM^{-1}_squared
Km0750_0483	p766	0.1 mM
Km0837_0483	p767	0.1 mM
Km0754_0483	p768	0.1 mM
Vmax_0486	p769	68.0634738371 mM·s^{-1}
Keq_0486	p770	0.0107844557 mM^{-1}
Km0764_0486	p771	0.079 mM
Km1198_0486	p772	1.5 mM
Km1322_0486	p773	0.1 mM
Km0075_0486	p774	0.000737 mM
Km1203_0486	p775	0.0867 mM
Vmax_0489	p776	0.311 mM·s^{-1}
Keq_0489	p777	0.4379562044 mM
Km0767_0489	p778	0.0685 mM
Km0765_0489	p779	0.15 mM
Km1322_0489	p780	0.1 mM
Vmax_0491	p781	0.452259852 mM·s^{-1}
Keq_0491	p782	8.1732490156
Km0629_0491	p783	0.29 mM
Km1203_0491	p784	0.0867 mM
Km0767_0491	p785	0.0685 mM
Km1198_0491	p786	1.5 mM
Vmax_0495	p787	0.016859852 mM·s^{-1}
Keq_0495	p788	2.9197080292
Km0380_0495	p789	0.1 mM
Km0767_0495	p790	0.0685 mM
Km0082_0495	p791	0.1 mM
Km0529_0495	p792	0.1 mM
Vmax_0499	p793	0.2933037983 mM·s^{-1}
Keq_0499	p794	2
Km0120_0499	p795	0.1 mM
Km0325_0499	p796	0.1 mM
Km0301_0499	p797	0.1 mM
Km1487_0499	p798	0.1 mM
Vmax_0502	p799	1.1684758001 mM·s^{-1}

Keq_0502	p800	1.95
Km1039_0502	p801	0.2 mM
Km1487_0502	p802	0.1 mM
Km0306_0502	p803	0.1 mM
Km1003_0502	p804	0.195 mM
Vmax_0510	p805	0.9982731186 mM·s^{-1}
Keq_0510	p806	0.1207708779 mM
Km1543_0510	p807	0.467 mM
Km0773_0510	p808	0.1 mM
Km1538_0510	p809	0.282 mM
Vmax_0514	p810	0.480706014 mM·s^{-1}
Keq_0514	p811	0.124751439 mM
Km0434_0514	p812	4.29 mM
Km0999_0514	p813	0.1 mM
Km1565_0514	p814	0.049 mM
Km0423_0514	p815	0.44 mM
Km0633_0514	p816	0.1 mM
Km0782_0514	p817	0.1 mM
Km0991_0514	p818	0.298 mM
Vmax_0525	p819	0.003115997 mM·s^{-1}
Keq_0525	p820	0.0020512821 mM_squared
Km0785_0525	p821	0.975 mM
Km0141_0525	p822	0.1 mM
Km0633_0525	p823	0.1 mM
Km0722_0525	p824	0.1 mM
Vmax_0528	p825	0.0044835735 mM·s^{-1}
Keq_0528	p826	0.6013986014
Km0434_0528	p827	4.29 mM
Km0782_0528	p828	0.1 mM
Km0394_0528	p829	1.29 mM
Km0739_0528	p830	0.1 mM
Vmax_0529	p831	0.0044835735 mM·s^{-1}
Keq_0529	p832	1.1428571429
Km0586_0529	p833	0.175 mM
Km0782_0529	p834	0.1 mM
Km0582_0529	p835	0.1 mM
Km0739_0529	p836	0.1 mM
Vmax_0534	p837	52.08 mM·s^{-1}
Keq_0534	p838	0.0739298918
Km0434_0534	p839	4.29 mM
Km0563_0534	p840	1.57 mM
Km0394_0534	p841	1.29 mM
Km0568_0534	p842	0.193 mM
Vmax_0536	p843	0.4327773636 mM·s^{-1}
Keq_0536	p844	0.00668168
Km1010_0536	p845	0.1 mM
Km1198_0536	p846	1.5 mM
Km1006_0536	p847	0.1 mM
Km1203_0536	p848	0.0867 mM
Vmax_0537	p849	0.1442591212 mM·s^{-1}
Keq_0537	p850	0.2 mM
Km1011_0537	p851	0.1 mM
Km1010_0537	p852	0.1 mM
Km1322_0537	p853	0.1 mM

Vmax_0538	p854	0.2019627697 mM·s^{-1}
Keq_0538	p855	1.1744966443
Km0207_0538	p856	0.1 mM
Km0991_0538	p857	0.298 mM
Km0180_0538	p858	0.175 mM
Km1011_0538	p859	0.1 mM
Vmax_0542	p860	0.2758234397 mM·s^{-1}
Keq_0542	p861	2
Km0454_0542	p862	0.1 mM
Km0836_0542	p863	0.1 mM
Vmax_0543	p864	0.643588026 mM·s^{-1}
Keq_0543	p865	1.1428571429
Km0180_0543	p866	0.175 mM
Km0373_0543	p867	0.1 mM
Km0529_0543	p868	0.1 mM
Km0835_0543	p869	0.1 mM
Vmax_0545	p870	1.0113526123 mM·s^{-1}
Keq_0545	p871	0.01156 mM
Km0836_0545	p872	0.1 mM
Km1198_0545	p873	1.5 mM
Km0176_0545	p874	0.1 mM
Km1203_0545	p875	0.0867 mM
Km0456_0545	p876	0.1 mM
Vmax_0547	p877	1.1282986732 mM·s^{-1}
Keq_0547	p878	2
Km0978_0547	p879	0.1 mM
Km1212_0547	p880	0.1 mM
Km1014_0547	p881	0.1 mM
Km1207_0547	p882	0.1 mM
Vmax_0548	p883	0.530488875 mM·s^{-1}
Keq_0548	p884	0.6013986014
Km0434_0548	p885	4.29 mM
Km1014_0548	p886	0.1 mM
Km0394_0548	p887	1.29 mM
Km1238_0548	p888	0.1 mM
Vmax_0549	p889	0.5978097982 mM·s^{-1}
Keq_0549	p890	2
Km0373_0549	p891	0.1 mM
Km1014_0549	p892	0.1 mM
Km0529_0549	p893	0.1 mM
Km1233_0549	p894	0.1 mM
Vmax_0550	p895	0.1183117139 mM·s^{-1}
Keq_0550	p896	20 mM^{-1}
Km0837_0550	p897	0.1 mM
Km1616_0550	p898	0.1 mM
Km1620_0550	p899	0.1 mM
Vmax_0553	p900	18.0455211578 mM·s^{-1}
Keq_0553	p901	0.2 mM
Km0033_0553	p902	0.1 mM
Km0025_0553	p903	0.1 mM
Km0750_0553	p904	0.1 mM
Vmax_0558	p905	0.3636483927 mM·s^{-1}
Keq_0558	p906	0.2 mM
Km0218_0558	p907	0.1 mM

Km1212_0558	p908	0.1 mM
Km0028_0558	p909	0.1 mM
Km0529_0558	p910	0.1 mM
Km1207_0558	p911	0.1 mM
Vmax_0559	p912	0.1106755978 mM·s^{-1}
Keq_0559	p913	2
Km0367_0559	p914	0.1 mM
Km0373_0559	p915	0.1 mM
Km0218_0559	p916	0.1 mM
Km0529_0559	p917	0.1 mM
Vmax_0563	p918	0.3173700666 mM·s^{-1}
Keq_0563	p919	0.596 mM
Km0312_0563	p920	0.1 mM
Km0999_0563	p921	0.1 mM
Km0403_0563	p922	0.1 mM
Km0550_0563	p923	0.1 mM
Km0991_0563	p924	0.298 mM
Vmax_0564	p925	0.0865554727 mM·s^{-1}
Keq_0564	p926	2
Km0550_0564	p927	0.1 mM
Km0207_0564	p928	0.1 mM
Vmax_0565	p929	0.1463018303 mM·s^{-1}
Keq_0565	p930	0.0349654321
Km0849_0565	p931	0.162 mM
Km1198_0565	p932	1.5 mM
Km1203_0565	p933	0.0867 mM
Km1565_0565	p934	0.049 mM
Vmax_0566	p935	0.3067913892 mM·s^{-1}
Keq_0566	p936	0.2 mM
Km0076_0566	p937	0.1 mM
Km0086_0566	p938	0.1 mM
Km0456_0566	p939	0.1 mM
Vmax_0568	p940	6.6911327679 mM·s^{-1}
Keq_0568	p941	0.2 mM
Km0633_0568	p942	0.1 mM
Km1322_0568	p943	0.1 mM
Vmax_0570	p944	0.2122571005 mM·s^{-1}
Keq_0570	p945	3.24
Km1365_0570	p946	0.1 mM
Km0849_0570	p947	0.162 mM
Vmax_0594	p948	0.0014504293 mM·s^{-1}
Keq_0594	p949	2
Km0089_0594	p950	0.1 mM
Km0499_0594	p951	0.1 mM
Km0619_0594	p952	0.1 mM
Km0918_0594	p953	0.1 mM
Vmax_0658	p954	2.2004324618 mM·s^{-1}
Keq_0658	p955	0.02023 mM
Km0940_0658	p956	0.1 mM
Km1198_0658	p957	1.5 mM
Km0180_0658	p958	0.175 mM
Km0456_0658	p959	0.1 mM
Km1203_0658	p960	0.0867 mM
Vmax_0661	p961	2.2004324618 mM·s^{-1}

Keq_0661	p962	0.35 mM
Km0940_0661	p963	0.1 mM
Km1207_0661	p964	0.1 mM
Km0180_0661	p965	0.175 mM
Km0456_0661	p966	0.1 mM
Km1212_0661	p967	0.1 mM
Vmax_0663	p968	0.4631679518 mM·s^{-1}
Keq_0663	p969	1.1744966443
Km0056_0663	p970	0.1 mM
Km0991_0663	p971	0.298 mM
Km0180_0663	p972	0.175 mM
Km1016_0663	p973	0.1 mM
Vmax_0667	p974	0.0158107997 mM·s^{-1}
Keq_0667	p975	2
Km0943_0667	p976	0.1 mM
Km1376_0667	p977	0.1 mM
Vmax_0669	p978	0.4631679518 mM·s^{-1}
Keq_0669	p979	2
Km0039_0669	p980	0.1 mM
Km1212_0669	p981	0.1 mM
Km0008_0669	p982	0.1 mM
Km1207_0669	p983	0.1 mM
Vmax_0670	p984	0.2529346506 mM·s^{-1}
Keq_0670	p985	0.01906 mM
Km1020_0670	p986	0.1 mM
Km0427_0670	p987	0.1 mM
Km0955_0670	p988	0.00953 mM
Vmax_0674	p989	0.6072342727 mM·s^{-1}
Keq_0674	p990	0.021239
Km0991_0674	p991	0.298 mM
Km1399_0674	p992	0.527 mM
Km0180_0674	p993	0.175 mM
Km0955_0674	p994	0.00953 mM
Vmax_0678	p995	0.643588026 mM·s^{-1}
Keq_0678	p996	2
Km0953_0678	p997	0.1 mM
Km1212_0678	p998	0.1 mM
Km0959_0678	p999	0.1 mM
Km1207_0678	p1000	0.1 mM
Vmax_0688	p1001	13.9503755002 mM·s^{-1}
Keq_0688	p1002	2
Km1151_0688	p1003	0.1 mM
Km1212_0688	p1004	0.1 mM
Km0062_0688	p1005	0.1 mM
Km1207_0688	p1006	0.1 mM
Vmax_0694	p1007	0.2529346506 mM·s^{-1}
Keq_0694	p1008	20 mM^{-1}
Km1048_0694	p1009	0.1 mM
Km1275_0694	p1010	0.1 mM
Km1195_0694	p1011	0.1 mM
Vmax_0696	p1012	13.9503755002 mM·s^{-1}
Keq_0696	p1013	0.1156
Km0062_0696	p1014	0.1 mM
Km1198_0696	p1015	1.5 mM

Km0063_0696	p1016	0.1 mM
Km1203_0696	p1017	0.0867 mM
Vmax_0697	p1018	18.0455211578 mM·s^{-1}
Keq_0697	p1019	20 mM^{-1}
Km0750_0697	p1020	0.1 mM
Km1151_0697	p1021	0.1 mM
Km0033_0697	p1022	0.1 mM
Vmax_0698	p1023	0.0079053998 mM·s^{-1}
Keq_0698	p1024	2
Km0037_0698	p1025	0.1 mM
Km1059_0698	p1026	0.1 mM
Vmax_0699	p1027	0.6732092322 mM·s^{-1}
Keq_0699	p1028	1.1744966443
Km0291_0699	p1029	0.1 mM
Km0991_0699	p1030	0.298 mM
Km0180_0699	p1031	0.175 mM
Km1021_0699	p1032	0.1 mM
Vmax_0713	p1033	1.0084943582 mM·s^{-1}
Keq_0713	p1034	0.1156
Km0066_0713	p1035	0.1 mM
Km1198_0713	p1036	1.5 mM
Km1203_0713	p1037	0.0867 mM
Km1271_0713	p1038	0.1 mM
Vmax_0722	p1039	2.2108191185 mM·s^{-1}
Keq_0722	p1040	0.2051282051
Km0573_0722	p1041	0.1 mM
Km0785_0722	p1042	0.975 mM
Km0633_0722	p1043	0.1 mM
Km0743_0722	p1044	0.1 mM
Vmax_0723	p1045	0.9474939079 mM·s^{-1}
Keq_0723	p1046	3.3955857385
Km0557_0723	p1047	0.0589 mM
Km0574_0723	p1048	0.1 mM
Vmax_0724	p1049	0.4211708517 mM·s^{-1}
Keq_0724	p1050	2
Km0304_0724	p1051	0.1 mM
Km0120_0724	p1052	0.1 mM
Vmax_0726	p1053	0.0651116429 mM·s^{-1}
Keq_0726	p1054	0.0046620047 mM
Km0434_0726	p1055	4.29 mM
Km1029_0726	p1056	0.1 mM
Km0633_0726	p1057	0.1 mM
Km1322_0726	p1058	0.1 mM
Km1416_0726	p1059	0.1 mM
Vmax_0727	p1060	0.1760765283 mM·s^{-1}
Keq_0727	p1061	2
Km0322_0727	p1062	0.1 mM
Km1012_0727	p1063	0.1 mM
Km1029_0727	p1064	0.1 mM
Km1487_0727	p1065	0.1 mM
Vmax_0731	p1066	0.4913659937 mM·s^{-1}
Keq_0731	p1067	0.1156
Km0306_0731	p1068	0.1 mM
Km1198_0731	p1069	1.5 mM

Km0304_0731	p1070	0.1 mM
Km1203_0731	p1071	0.0867 mM
Vmax_0732	p1072	0.4913659936 mM·s^{-1}
Keq_0732	p1073	2
Km0306_0732	p1074	0.1 mM
Km1207_0732	p1075	0.1 mM
Km0304_0732	p1076	0.1 mM
Km1212_0732	p1077	0.1 mM
Vmax_0736	p1078	0.1106755978 mM·s^{-1}
Keq_0736	p1079	2
Km0028_0736	p1080	0.1 mM
Km0539_0736	p1081	0.1 mM
Km0019_0736	p1082	0.1 mM
Km0467_0736	p1083	0.1 mM
Vmax_0739	p1084	0.300405194 mM·s^{-1}
Keq_0739	p1085	0.006013986 mM_squared
Km0018_0739	p1086	0.1 mM
Km0434_0739	p1087	4.29 mM
Km0394_0739	p1088	1.29 mM
Km0456_0739	p1089	0.1 mM
Km0943_0739	p1090	0.1 mM
Km1322_0739	p1091	0.1 mM
Vmax_0757	p1092	0.003978907 mM·s^{-1}
Keq_0757	p1093	0.2 mM
Km0126_0757	p1094	0.1 mM
Km1153_0757	p1095	0.1 mM
Km1322_0757	p1096	0.1 mM
Vmax_0758	p1097	0.0023873442 mM·s^{-1}
Keq_0758	p1098	1.0362694301
Km0568_0758	p1099	0.193 mM
Km0126_0758	p1100	0.1 mM
Vmax_0759	p1101	0.5754977208 mM·s^{-1}
Keq_0759	p1102	0.2 mM
Km1191_0759	p1103	0.1 mM
Km1212_0759	p1104	0.1 mM
Km0145_0759	p1105	0.1 mM
Km1207_0759	p1106	0.1 mM
Km1322_0759	p1107	0.1 mM
Vmax_0762	p1108	0.2529346506 mM·s^{-1}
Keq_0762	p1109	0.2 mM
Km1195_0762	p1110	0.1 mM
Km0722_0762	p1111	0.1 mM
Km1020_0762	p1112	0.1 mM
Vmax_0770	p1113	0.4426356386 mM·s^{-1}
Keq_0770	p1114	34.6020761246
Km1203_0770	p1115	0.0867 mM
Km1537_0770	p1116	0.1 mM
Km1198_0770	p1117	1.5 mM
Km1535_0770	p1118	0.1 mM
Vmax_0792	p1119	0.0845961934 mM·s^{-1}
Keq_0792	p1120	0.2 mM
Km0467_0792	p1121	0.1 mM
Km0526_0792	p1122	0.1 mM
Km1322_0792	p1123	0.1 mM

Vmax_0800	p1124	2.5622074094 mM·s^{-1}
Keq_0800	p1125	5.8636363636
Km0434_0800	p1126	4.29 mM
Km0739_0800	p1127	0.1 mM
Km0394_0800	p1128	1.29 mM
Km0785_0800	p1129	0.975 mM
Vmax_0806	p1130	0.0078791928 mM·s^{-1}
Keq_0806	p1131	0.2 mM
Km0539_0806	p1132	0.1 mM
Km0467_0806	p1133	0.1 mM
Km1322_0806	p1134	0.1 mM
Vmax_0811	p1135	4.7902015737 mM·s^{-1}
Keq_0811	p1136	1.3840698309
Km0434_0811	p1137	4.29 mM
Km1538_0811	p1138	0.282 mM
Km0394_0811	p1139	1.29 mM
Km1559_0811	p1140	0.649 mM
Vmax_0813	p1141	0.1924031986 mM·s^{-1}
Keq_0813	p1142	1116
Km0841_0813	p1143	0.1 mM
Km1233_0813	p1144	0.1 mM
Km0362_0813	p1145	55.8 mM
Km1012_0813	p1146	0.1 mM
Vmax_0816	p1147	0.3662258223 mM·s^{-1}
Keq_0816	p1148	2
Km0455_0816	p1149	0.1 mM
Km1266_0816	p1150	0.1 mM
Km0979_0816	p1151	0.1 mM
Km1322_0816	p1152	0.1 mM
Vmax_0818	p1153	0.3662258223 mM·s^{-1}
Keq_0818	p1154	0.6711409396
Km0991_0818	p1155	0.298 mM
Km1182_0818	p1156	0.1 mM
Km1192_0818	p1157	0.1 mM
Km1266_0818	p1158	0.1 mM
Vmax_0820	p1159	0.331272799 mM·s^{-1}
Keq_0820	p1160	2
Km1269_0820	p1161	0.1 mM
Km1386_0820	p1162	0.1 mM
Km0633_0820	p1163	0.1 mM
Km1270_0820	p1164	0.1 mM
Vmax_0821	p1165	0.2366234278 mM·s^{-1}
Keq_0821	p1166	0.2 mM
Km1270_0821	p1167	0.1 mM
Km0456_0821	p1168	0.1 mM
Km1545_0821	p1169	0.1 mM
Vmax_0851	p1170	0.3069834099 mM·s^{-1}
Keq_0851	p1171	1.1744966443
Km0951_0851	p1172	0.1 mM
Km0991_0851	p1173	0.298 mM
Km0180_0851	p1174	0.175 mM
Km1032_0851	p1175	0.1 mM
Vmax_0855	p1176	0.4609059687 mM·s^{-1}
Keq_0855	p1177	0.0601398601 mM

Km0302_0855	p1178	0.1 mM
Km0434_0855	p1179	4.29 mM
Km0300_0855	p1180	0.1 mM
Km0394_0855	p1181	1.29 mM
Km1322_0855	p1182	0.1 mM
Vmax_0858	p1183	0.0077553704 mM·s^{-1}
Keq_0858	p1184	2
Km1351_0858	p1185	0.1 mM
Km1416_0858	p1186	0.1 mM
Km1343_0858	p1187	0.1 mM
Km1413_0858	p1188	0.1 mM
Vmax_0874	p1189	0.0055704698 mM·s^{-1}
Keq_0874	p1190	2
Km0471_0874	p1191	0.1 mM
Km1153_0874	p1192	0.1 mM
Km0089_0874	p1193	0.1 mM
Km0526_0874	p1194	0.1 mM
Vmax_0877	p1195	0.0068801984 mM·s^{-1}
Keq_0877	p1196	0.2 mM
Km1337_0877	p1197	0.1 mM
Km0456_0877	p1198	0.1 mM
Km1351_0877	p1199	0.1 mM
Vmax_0880	p1200	0.0106367059 mM·s^{-1}
Keq_0880	p1201	1
Km0471_0880	p1202	0.1 mM
Km1039_0880	p1203	0.2 mM
Km0526_0880	p1204	0.1 mM
Km1337_0880	p1205	0.1 mM
Vmax_0883	p1206	0.3931157225 mM·s^{-1}
Keq_0883	p1207	0.2 mM
Km0201_0883	p1208	0.1 mM
Km1616_0883	p1209	0.1 mM
Km0390_0883	p1210	0.1 mM
Km1469_0883	p1211	0.1 mM
Km1620_0883	p1212	0.1 mM
Vmax_0886	p1213	35.0101324198 mM·s^{-1}
Keq_0886	p1214	11.742077956
Km0434_0886	p1215	4.29 mM
Km0557_0886	p1216	0.0589 mM
Km0394_0886	p1217	1.29 mM
Km0555_0886	p1218	1.15 mM
Vmax_0887	p1219	4.3906511702 mM·s^{-1}
Keq_0887	p1220	0.6013986014
Km0434_0887	p1221	4.29 mM
Km1427_0887	p1222	0.1 mM
Km0394_0887	p1223	1.29 mM
Km1426_0887	p1224	0.1 mM
Vmax_0888	p1225	1.9882923639 mM·s^{-1}
Keq_0888	p1226	1.3989637306
Km0568_0888	p1227	0.193 mM
Km0567_0888	p1228	0.135 mM
Vmax_0889	p1229	21.6709527908 mM·s^{-1}
Keq_0889	p1230	0.2 mM
Km0340_0889	p1231	0.1 mM

Km1207_0889	p1232	0.1 mM
Km0456_0889	p1233	0.1 mM
Km0577_0889	p1234	0.1 mM
Km1212_0889	p1235	0.1 mM
Vmax_0891	p1236	2.4095741643 mM·s^{-1}
Keq_0891	p1237	0.0988034188
Km0260_0891	p1238	0.117 mM
Km1198_0891	p1239	1.5 mM
Km0258_0891	p1240	0.1 mM
Km1203_0891	p1241	0.0867 mM
Vmax_0892	p1242	43.3131197144 mM·s^{-1}
Keq_0892	p1243	1055.8833738285
Km0075_0892	p1244	0.000737 mM
Km0394_0892	p1245	1.29 mM
Km0260_0892	p1246	0.117 mM
Km0434_0892	p1247	4.29 mM
Vmax_0893	p1248	17.5300909501 mM·s^{-1}
Keq_0893	p1249	0.2888888889
Km0260_0893	p1250	0.117 mM
Km0188_0893	p1251	0.0169 mM
Vmax_0900	p1252	0.0077553704 mM·s^{-1}
Keq_0900	p1253	2
Km1342_0900	p1254	0.1 mM
Km1416_0900	p1255	0.1 mM
Km1346_0900	p1256	0.1 mM
Km1413_0900	p1257	0.1 mM
Vmax_0901	p1258	0.0077553704 mM·s^{-1}
Keq_0901	p1259	2
Km1343_0901	p1260	0.1 mM
Km1416_0901	p1261	0.1 mM
Km1342_0901	p1262	0.1 mM
Km1413_0901	p1263	0.1 mM
Vmax_0902	p1264	0.9474939079 mM·s^{-1}
Keq_0902	p1265	2
Km0574_0902	p1266	0.1 mM
Km0573_0902	p1267	0.1 mM
Vmax_0904	p1268	0.1106755978 mM·s^{-1}
Keq_0904	p1269	0.6013986014
Km0019_0904	p1270	0.1 mM
Km0434_0904	p1271	4.29 mM
Km0018_0904	p1272	0.1 mM
Km0394_0904	p1273	1.29 mM
Vmax_0908	p1274	0.6285081392 mM·s^{-1}
Keq_0908	p1275	0.6013986014
Km0434_0908	p1276	4.29 mM
Km0973_0908	p1277	0.1 mM
Km1364_0908	p1278	0.1 mM
Km0299_0908	p1279	0.1 mM
Km0394_0908	p1280	1.29 mM
Km1322_0908	p1281	0.1 mM
Vmax_0909	p1282	0.0865554727 mM·s^{-1}
Keq_0909	p1283	2
Km0078_0909	p1284	0.1 mM
Km0077_0909	p1285	0.1 mM

Vmax_0910	p1286	0.1442591212 mM·s^{-1}
Keq_0910	p1287	0.2 mM
Km0326_0910	p1288	0.1 mM
Km0078_0910	p1289	0.1 mM
Km0633_0910	p1290	0.1 mM
Vmax_0911	p1291	0.6285081392 mM·s^{-1}
Keq_0911	p1292	0.6013986014
Km0300_0911	p1293	0.1 mM
Km0456_0911	p1294	0.1 mM
Km0434_0911	p1295	4.29 mM
Km1364_0911	p1296	0.1 mM
Km0394_0911	p1297	1.29 mM
Km1322_0911	p1298	0.1 mM
Vmax_0912	p1299	0.4952665679 mM·s^{-1}
Keq_0912	p1300	2
Km0120_0912	p1301	0.1 mM
Km0403_0912	p1302	0.1 mM
Km1365_0912	p1303	0.1 mM
Km1487_0912	p1304	0.1 mM
Vmax_0913	p1305	0.1840748335 mM·s^{-1}
Keq_0913	p1306	2
Km1187_0913	p1307	0.1 mM
Km0076_0913	p1308	0.1 mM
Vmax_0914	p1309	0.6285081392 mM·s^{-1}
Keq_0914	p1310	0.3084095392
Km0327_0914	p1311	0.1 mM
Km0434_0914	p1312	4.29 mM
Km1003_0914	p1313	0.195 mM
Km0325_0914	p1314	0.1 mM
Km0394_0914	p1315	1.29 mM
Km1322_0914	p1316	0.1 mM
Vmax_0915	p1317	0.4609059687 mM·s^{-1}
Keq_0915	p1318	0.596 mM
Km0999_0915	p1319	0.1 mM
Km1386_0915	p1320	0.1 mM
Km0327_0915	p1321	0.1 mM
Km0633_0915	p1322	0.1 mM
Km0991_0915	p1323	0.298 mM
Vmax_0916	p1324	1.2560473118 mM·s^{-1}
Keq_0916	p1325	0.2051282051
Km0434_0916	p1326	4.29 mM
Km1408_0916	p1327	0.1 mM
Km0423_0916	p1328	0.44 mM
Km1386_0916	p1329	0.1 mM
Vmax_0917	p1330	1.7211244031 mM·s^{-1}
Keq_0917	p1331	13.4228187919 mM
Km0259_0917	p1332	0.00298 mM
Km1039_0917	p1333	0.2 mM
Km1322_0917	p1334	0.1 mM
Vmax_0918	p1335	2.4095741643 mM·s^{-1}
Keq_0918	p1336	0.035
Km0258_0918	p1337	0.1 mM
Km0991_0918	p1338	0.298 mM
Km0180_0918	p1339	0.175 mM

Km0259_0918	p1340	0.00298 mM
Vmax_0919	p1341	0.0005180105 mM·s^{-1}
Keq_0919	p1342	20 mM^{-1}
Km1084_0919	p1343	0.1 mM
Km1366_0919	p1344	0.1 mM
Km0481_0919	p1345	0.1 mM
Vmax_0922	p1346	0.001139623 mM·s^{-1}
Keq_0922	p1347	20 mM^{-1}
Km1212_0922	p1348	0.1 mM
Km1275_0922	p1349	0.1 mM
Km1445_0922	p1350	0.1 mM
Km1207_0922	p1351	0.1 mM
Km1366_0922	p1352	0.1 mM
Vmax_0938	p1353	0.2192738642 mM·s^{-1}
Keq_0938	p1354	0.2 mM
Km1377_0938	p1355	0.1 mM
Km0456_0938	p1356	0.1 mM
Km0951_0938	p1357	0.1 mM
Vmax_0939	p1358	0.4083494857 mM·s^{-1}
Keq_0939	p1359	0.2 mM
Km1207_0939	p1360	0.1 mM
Km1377_0939	p1361	0.1 mM
Km0204_0939	p1362	0.1 mM
Km0456_0939	p1363	0.1 mM
Km1212_0939	p1364	0.1 mM
Vmax_0957	p1365	0.3473759638 mM·s^{-1}
Keq_0957	p1366	2
Km0118_0957	p1367	0.1 mM
Km1212_0957	p1368	0.1 mM
Km1035_0957	p1369	0.1 mM
Km1207_0957	p1370	0.1 mM
Vmax_0958	p1371	11.1138381034 mM·s^{-1}
Keq_0958	p1372	0.1141173817
Km0434_0958	p1373	4.29 mM
Km0445_0958	p1374	0.1 mM
Km1399_0958	p1375	0.527 mM
Km0394_0958	p1376	1.29 mM
Km1271_0958	p1377	0.1 mM
Km1322_0958	p1378	0.1 mM
Vmax_0959	p1379	45.7829965006 mM·s^{-1}
Keq_0959	p1380	0.0675521822 mM
Km1399_0959	p1381	0.527 mM
Km0359_0959	p1382	0.178 mM
Km0456_0959	p1383	0.1 mM
Vmax_0961	p1384	12.4820895805 mM·s^{-1}
Keq_0961	p1385	0.0219354839
Km0529_0961	p1386	0.1 mM
Km1198_0961	p1387	1.5 mM
Km1399_0961	p1388	0.527 mM
Km0373_0961	p1389	0.1 mM
Km0456_0961	p1390	0.1 mM
Km1203_0961	p1391	0.0867 mM
Vmax_0962	p1392	39.6190623352 mM·s^{-1}
Keq_0962	p1393	22.9095607235

Km0394_0962	p1394	1.29 mM
Km1360_0962	p1395	0.153 mM
Km0434_0962	p1396	4.29 mM
Km1399_0962	p1397	0.527 mM
Vmax_0967	p1398	0.0048471065 mM·s^{-1}
Keq_0967	p1399	2
Km0158_0967	p1400	0.1 mM
Km0314_0967	p1401	0.1 mM
Km0328_0967	p1402	0.1 mM
Km1322_0967	p1403	0.1 mM
Vmax_0968	p1404	0.0024235532 mM·s^{-1}
Keq_0968	p1405	2
Km0328_0968	p1406	0.1 mM
Km0314_0968	p1407	0.1 mM
Km1405_0968	p1408	0.1 mM
Vmax_0970	p1409	0.0044835735 mM·s^{-1}
Keq_0970	p1410	0.0815850816
Km0434_0970	p1411	4.29 mM
Km1616_0970	p1412	0.1 mM
Km0586_0970	p1413	0.175 mM
Km1620_0970	p1414	0.1 mM
Vmax_0973	p1415	0.0129390814 mM·s^{-1}
Keq_0973	p1416	0.3081664099
Km1559_0973	p1417	0.649 mM
Km1616_0973	p1418	0.1 mM
Km0656_0973	p1419	0.1 mM
Km1620_0973	p1420	0.1 mM
Vmax_0974	p1421	0.0051837111 mM·s^{-1}
Keq_0974	p1422	0.1550387597
Km0394_0974	p1423	1.29 mM
Km1616_0974	p1424	0.1 mM
Km0582_0974	p1425	0.1 mM
Km1620_0974	p1426	0.1 mM
Vmax_0976	p1427	0.0032717969 mM·s^{-1}
Keq_0976	p1428	2
Km0467_0976	p1429	0.1 mM
Km1616_0976	p1430	0.1 mM
Km0587_0976	p1431	0.1 mM
Km1620_0976	p1432	0.1 mM
Vmax_0978	p1433	0.0065435937 mM·s^{-1}
Keq_0978	p1434	2
Km0739_0978	p1435	0.1 mM
Km1616_0978	p1436	0.1 mM
Km0613_0978	p1437	0.1 mM
Km1620_0978	p1438	0.1 mM
Vmax_0982	p1439	2.4200136351 mM·s^{-1}
Keq_0982	p1440	2
Km0577_0982	p1441	0.1 mM
Km1408_0982	p1442	0.1 mM
Vmax_0984	p1443	3.4881688855 mM·s^{-1}
Keq_0984	p1444	2
Km0577_0984	p1445	0.1 mM
Km0581_0984	p1446	0.1 mM
Vmax_0986	p1447	0.0181685708 mM·s^{-1}

Keq_0986	p1448	2
Km1416_0986	p1449	0.1 mM
Km1569_0986	p1450	0.1 mM
Km0700_0986	p1451	0.1 mM
Km1413_0986	p1452	0.1 mM
Vmax_0988	p1453	1.0113526123 mM·s^{-1}
Keq_0988	p1454	0.02023 mM
Km1038_0988	p1455	0.1 mM
Km1198_0988	p1456	1.5 mM
Km0180_0988	p1457	0.175 mM
Km1025_0988	p1458	0.1 mM
Km1203_0988	p1459	0.0867 mM
Vmax_0989	p1460	1.0113526123 mM·s^{-1}
Keq_0989	p1461	6.711409396 mM^{-1}
Km0959_0989	p1462	0.1 mM
Km0991_0989	p1463	0.298 mM
Km1212_0989	p1464	0.1 mM
Km1038_0989	p1465	0.1 mM
Km1207_0989	p1466	0.1 mM
Vmax_0990	p1467	3.1361794073 mM·s^{-1}
Keq_0990	p1468	0.58 mM
Km1426_0990	p1469	0.1 mM
Km0551_0990	p1470	0.1 mM
Km0629_0990	p1471	0.29 mM
Vmax_0992	p1472	0.0577613521 mM·s^{-1}
Keq_0992	p1473	1
Km0373_0992	p1474	0.1 mM
Km1039_0992	p1475	0.2 mM
Km0529_0992	p1476	0.1 mM
Km1234_0992	p1477	0.1 mM
Vmax_0993	p1478	0.002279246 mM·s^{-1}
Keq_0993	p1479	0.1 mM
Km1039_0993	p1480	0.2 mM
Km1302_0993	p1481	0.1 mM
Km0231_0993	p1482	0.1 mM
Km0456_0993	p1483	0.1 mM
Km0529_0993	p1484	0.1 mM
Vmax_0996	p1485	0.6422416075 mM·s^{-1}
Keq_0996	p1486	2
Km0211_0996	p1487	0.1 mM
Km1212_0996	p1488	0.1 mM
Km1207_0996	p1489	0.1 mM
Km1429_0996	p1490	0.1 mM
Vmax_0997	p1491	0.6422416075 mM·s^{-1}
Keq_0997	p1492	0.6013986014
Km0434_0997	p1493	4.29 mM
Km1429_0997	p1494	0.1 mM
Km0261_0997	p1495	0.1 mM
Km0394_0997	p1496	1.29 mM
Vmax_1010	p1497	0.014493233 mM·s^{-1}
Keq_1010	p1498	346.0207612457 mM^{-1}
Km1203_1010	p1499	0.0867 mM
Km1275_1010	p1500	0.1 mM
Km1447_1010	p1501	0.1 mM

Km0037_1010	p1502	0.1 mM
Km1198_1010	p1503	1.5 mM
Vmax_1011	p1504	0.014493233 mM·s^{-1}
Keq_1011	p1505	20 mM^{-1}
Km1212_1011	p1506	0.1 mM
Km1275_1011	p1507	0.1 mM
Km1447_1011	p1508	0.1 mM
Km0037_1011	p1509	0.1 mM
Km1207_1011	p1510	0.1 mM
Vmax_1012	p1511	0.0606080654 mM·s^{-1}
Keq_1012	p1512	0.2 mM
Km0190_1012	p1513	0.1 mM
Km1212_1012	p1514	0.1 mM
Km0633_1012	p1515	0.1 mM
Km1207_1012	p1516	0.1 mM
Km1447_1012	p1517	0.1 mM
Vmax_1014	p1518	0.0015618454 mM·s^{-1}
Keq_1014	p1519	20 mM^{-1}
Km0666_1014	p1520	0.1 mM
Km0595_1014	p1521	0.1 mM
Km0672_1014	p1522	0.1 mM
Vmax_1026	p1523	0.2501645507 mM·s^{-1}
Keq_1026	p1524	0.1550387597
Km0394_1026	p1525	1.29 mM
Km1467_1026	p1526	0.1 mM
Km0298_1026	p1527	0.1 mM
Km1322_1026	p1528	0.1 mM
Vmax_1027	p1529	1.1078715816 mM·s^{-1}
Keq_1027	p1530	2
Km1212_1027	p1531	0.1 mM
Km1469_1027	p1532	0.1 mM
Km0841_1027	p1533	0.1 mM
Km1207_1027	p1534	0.1 mM
Vmax_1038	p1535	0.4482227068 mM·s^{-1}
Keq_1038	p1536	2
Km1212_1038	p1537	0.1 mM
Km1620_1038	p1538	0.1 mM
Km1207_1038	p1539	0.1 mM
Km1616_1038	p1540	0.1 mM
Vmax_1041	p1541	0.378920625 mM·s^{-1}
Keq_1041	p1542	0.354 mM
Km1238_1041	p1543	0.1 mM
Km1045_1041	p1544	0.177 mM
Km1322_1041	p1545	0.1 mM
Vmax_1045	p1546	0.0096672846 mM·s^{-1}
Keq_1045	p1547	2
Km0306_1045	p1548	0.1 mM
Km0654_1045	p1549	0.1 mM
Km0625_1045	p1550	0.1 mM
Km0649_1045	p1551	0.1 mM
Vmax_1049	p1552	4.3906511702 mM·s^{-1}
Keq_1049	p1553	1.58
Km0581_1049	p1554	0.1 mM
Km1408_1049	p1555	0.1 mM

Km0764_1049	p1556	0.079 mM
Km1427_1049	p1557	0.1 mM
Vmax_1050	p1558	3.7484095627 mM·s^{-1}
Keq_1050	p1559	0.93062
Km0551_1050	p1560	0.1 mM
Km0581_1050	p1561	0.1 mM
Km0557_1050	p1562	0.0589 mM
Km0764_1050	p1563	0.079 mM
Vmax_1051	p1564	0.1228088458 mM·s^{-1}
Keq_1051	p1565	0.0385 mM
Km0409_1051	p1566	0.02 mM
Km1322_1051	p1567	0.1 mM
Km1520_1051	p1568	0.00385 mM
Vmax_1052	p1569	0.0255877212 mM·s^{-1}
Keq_1052	p1570	20 mM^{-1}
Km0619_1052	p1571	0.1 mM
Km0595_1052	p1572	0.1 mM
Km1524_1052	p1573	0.1 mM
Vmax_1054	p1574	0.1138205928 mM·s^{-1}
Keq_1054	p1575	7.3417721519
Km0764_1054	p1576	0.079 mM
Km0629_1054	p1577	0.29 mM
Vmax_1055	p1578	0.4295079449 mM·s^{-1}
Keq_1055	p1579	0.79
Km0086_1055	p1580	0.1 mM
Km1039_1055	p1581	0.2 mM
Km0764_1055	p1582	0.079 mM
Km1048_1055	p1583	0.1 mM
Vmax_1063	p1584	0.2598587636 mM·s^{-1}
Keq_1063	p1585	1.1744966443
Km0204_1063	p1586	0.1 mM
Km0991_1063	p1587	0.298 mM
Km0180_1063	p1588	0.175 mM
Km1051_1063	p1589	0.1 mM
Vmax_1072	p1590	0.1508527248 mM·s^{-1}
Keq_1072	p1591	1.6959440559
Km0434_1072	p1592	4.29 mM
Km1545_1072	p1593	0.1 mM
Km0394_1072	p1594	1.29 mM
Km1538_1072	p1595	0.282 mM
Vmax_1084	p1596	4.639348849 mM·s^{-1}
Keq_1084	p1597	1.0660275067
Km0567_1084	p1598	0.135 mM
Km1559_1084	p1599	0.649 mM
Km0633_1084	p1600	0.1 mM
Km1543_1084	p1601	0.467 mM
Vmax_1087	p1602	0.6920590907 mM·s^{-1}
Keq_1087	p1603	1.1744966443
Km0232_1087	p1604	0.1 mM
Km0991_1087	p1605	0.298 mM
Km0180_1087	p1606	0.175 mM
Km1056_1087	p1607	0.1 mM
Vmax_1106	p1608	0.1586 mM·s^{-1}
Km0362_1106	p1609	55.8 mM

Vmax_1115	p1610	3.663578114 mM·s^{-1}
Km0420_1115	p1611	1 mM
Km0419_1115	p1612	0.1 mM
Vmax_1166	p1613	11.4019052879 mM·s^{-1}
Km0565_1166	p1614	74 mM
Km0563_1166	p1615	1.57 mM
Vmax_1172	p1616	0.0622 mM·s^{-1}
Km0765_1172	p1617	0.15 mM
Vmax_1244	p1618	0.1519905285 mM·s^{-1}
Km1324_1244	p1619	1 mM
Km1322_1244	p1620	0.1 mM
Vmax_1266	p1621	0.0723860213 mM·s^{-1}
Km1468_1266	p1622	1 mM
Km1467_1266	p1623	0.1 mM
Vmax_1664	p1624	2.7017716655 mM·s^{-1}
Keq_1664	p1625	2
Km0456_1664	p1626	0.1 mM
Km0445_1664	p1627	0.1 mM
Vmax_1697	p1628	12.22 mM·s^{-1}
Km0456_1697	p1629	0.1 mM
Vmax_1704	p1630	0.0032717969 mM·s^{-1}
Keq_1704	p1631	6.6511627907
Km0394_1704	p1632	1.29 mM
Km0587_1704	p1633	0.1 mM
Km0434_1704	p1634	4.29 mM
Km0589_1704	p1635	0.1 mM
Vmax_1729	p1636	0.0096672846 mM·s^{-1}
Keq_1729	p1637	6.6511627907
Km0394_1729	p1638	1.29 mM
Km0582_1729	p1639	0.1 mM
Km0434_1729	p1640	4.29 mM
Km0584_1729	p1641	0.1 mM
Vmax_1762	p1642	9.12 mM·s^{-1}
Km0680_1762	p1643	55.5 mM
Vmax_1936	p1644	28.0100750866 mM·s^{-1}
Keq_1936	p1645	0.0689655172 mM
Km0629_1936	p1646	0.29 mM
Km1151_1936	p1647	0.1 mM
Km1322_1936	p1648	0.1 mM
Vmax_1979	p1649	4.9371638398 mM·s^{-1}
Km1277_1979	p1650	1 mM
Km1275_1979	p1651	0.1 mM
Vmax_2030	p1652	0.0017311095 mM·s^{-1}
Keq_2030	p1653	0.2 mM
Km0313_2030	p1654	0.1 mM
Km0314_2030	p1655	0.1 mM
Km1322_2030	p1656	0.1 mM
Vmax_2079	p1657	0.01556 mM·s^{-1}
Km1520_2079	p1658	0.00385 mM
V0_2111	p1659	0.1923454949 mM·s^{-1}
ic0002_2111	p1660	0.1 mM
ep0002_2111	p1661	1.14
ic0423_2111	p1662	0.44 mM
ep0423_2111	p1663	0.051

ic0434_2111	p1664	4.29 mM
ep0434_2111	p1665	59.3
ic0526_2111	p1666	0.1 mM
ep0526_2111	p1667	0.05
ic0584_2111	p1668	0.1 mM
ep0584_2111	p1669	0.00359
ic0589_2111	p1670	0.1 mM
ep0589_2111	p1671	0.00243
ic0615_2111	p1672	0.1 mM
ep0615_2111	p1673	0.00243
ic0649_2111	p1674	0.1 mM
ep0649_2111	p1675	0.00359
ic0773_2111	p1676	0.1 mM
ep0773_2111	p1677	0.519
ic0782_2111	p1678	0.1 mM
ep0782_2111	p1679	0.051
ic0955_2111	p1680	0.00953 mM
ep0955_2111	p1681	0.357
ic0965_2111	p1682	0.1 mM
ep0965_2111	p1683	0.136
ic0969_2111	p1684	0.1 mM
ep0969_2111	p1685	0.172
ic0973_2111	p1686	0.1 mM
ep0973_2111	p1687	0.172
ic0981_2111	p1688	0.1 mM
ep0981_2111	p1689	0.0429
ic0991_2111	p1690	0.298 mM
ep0991_2111	p1691	0.268
ic0999_2111	p1692	0.1 mM
ep0999_2111	p1693	0.268
ic1003_2111	p1694	0.195 mM
ep1003_2111	p1695	0.325
ic1006_2111	p1696	0.1 mM
ep1006_2111	p1697	0.075
ic1016_2111	p1698	0.1 mM
ep1016_2111	p1699	0.172
ic1021_2111	p1700	0.1 mM
ep1021_2111	p1701	0.25
ic1025_2111	p1702	0.1 mM
ep1025_2111	p1703	0.239
ic1029_2111	p1704	0.1 mM
ep1029_2111	p1705	0.05
ic1032_2111	p1706	0.1 mM
ep1032_2111	p1707	0.114
ic1035_2111	p1708	0.1 mM
ep1035_2111	p1709	0.129
ic1039_2111	p1710	0.2 mM
ep1039_2111	p1711	0.254
ic1045_2111	p1712	0.177 mM
ep1045_2111	p1713	0.197
ic1048_2111	p1714	0.1 mM
ep1048_2111	p1715	0.028
ic1051_2111	p1716	0.1 mM
ep1051_2111	p1717	0.0965

ic1056_2111	p1718	0.1 mM
ep1056_2111	p1719	0.257
ic1107_2111	p1720	0.1 mM
ep1107_2111	p1721	0.821
ic1405_2111	p1722	0.1 mM
ep1405_2111	p1723	0.0009
ic1467_2111	p1724	0.1 mM
ep1467_2111	p1725	0.02
ic1520_2111	p1726	0.00385 mM
ep1520_2111	p1727	0.0234
ic1545_2111	p1728	0.1 mM
ep1545_2111	p1729	0.067
ic0089_2111	p1730	0.1 mM
ep0089_2111	p1731	0.00153
ic0122_2111	p1732	0.1 mM
ep0122_2111	p1733	$5.6 \cdot 10^{-5}$
ic0918_2111	p1734	0.1 mM
ep0918_2111	p1735	0.000538625
ic0657_2111	p1736	0.1 mM
ep0657_2111	p1737	$9.6 \cdot 10^{-5}$
ic0662_2111	p1738	0.1 mM
ep0662_2111	p1739	0.000125
ic0666_2111	p1740	0.1 mM
ep0666_2111	p1741	0.0056
ic0672_2111	p1742	0.1 mM
ep0672_2111	p1743	0.000812
ic0595_2111	p1744	0.1 mM
ep0595_2111	p1745	0.0005356
ic0700_2111	p1746	0.1 mM
ep0700_2111	p1747	0.000114
ic1059_2111	p1748	0.1 mM
ep1059_2111	p1749	$3.2 \cdot 10^{-5}$
ic1337_2111	p1750	0.1 mM
ep1337_2111	p1751	0.000373
ic1346_2111	p1752	0.1 mM
ep1346_2111	p1753	0.00288
ic1351_2111	p1754	0.1 mM
ep1351_2111	p1755	0.000697
ic1524_2111	p1756	0.1 mM
ep1524_2111	p1757	0.000781
ic1569_2111	p1758	0.1 mM
ep1569_2111	p1759	$1.5 \cdot 10^{-5}$

Table S12. Benchmark 1: Experimental concentrations for one extracellular and 37 intracellular metabolites.

observable number	name	compart–ment	concentration mM	SEM %	source	state #
1	1,3-bisphospho-D-glycerate	cell	0,000737		G.U.	x(19) = 0.000737
2	2-oxoglutarate	cell	0,175	42	M.U.	x(39) = 0.175
3	2-phospho-D-glyceric acid	cell	0,0169	18	G.U.	x(40) = 0.0169
4	3-phospho-serine	cell	0,00298	62	M.U.	x(52) = 0.00298
5	3-phosphoglycerate	cell	0,117	18	G.U.	x(53) = 0.117
6	acetaldehyde	cell	0,178		G.U.	x(79) = 0.178
7	acetate	cell	55,8	46	G.U.	x(80) = 55.8
8	ADP	cell	1,29		G.U.	x(87) = 1.29
9	trehalose 6-phosphate	cell	0,02		G.U.	x(89) = 0.02
10	AMP	cell	0,44		G.U.	x(91) = 0.44
11	ATP	cell	4,29		G.U.	x(93) = 4.29
12	D-fructose 1,6-bisphosphate	cell	1,15	20	G.U.	x(112) = 1.15
13	D-fructose 6-phosphate	cell	0,0589	15	G.U.	x(113) = 0.0589
14	D-glucose	cell	1,57	5	G.U.	x(114) = 1.57
15	D-glucose 1-phosphate	cell	0,135		G.U.	x(115) = 0.135
16	D-glucose 6-phosphate	cell	0,193	10	G.U.	x(116) = 0.193
17	dATP	cell	0,18		[4]	x(123) = 0.175
18	dihydroxyacetone phosphate	cell	0,29	19	G.U.	x(132) = 0.29
19	ethanol	cell	55,5	42	G.U.	x(144) = 55.5
20	glycerol	cell	0,15		G.U.	x(156) = 0.15
21	glyceraldehyde 3-phosphate	cell	0,079	27	G.U.	x(155) = 0.079
22	glycerol 3-phosphate	cell	0,0685	24	G.U.	x(157) = 0.0685
23	GTP	cell	0,98		[4]	x(160) = 0.975
24	IMP	cell	0,16		[4]	x(165) = 0.162
25	L-alanine	cell	0,00953	38	M.U.	x(171) = 0.00953
26	L-glutamate	cell	0,298	18	M.U.	x(180) = 0.298
27	L-glycine	cell	0,195	37	M.U.	x(182) = 0.195
28	L-serine	cell	0,2	15	M.U.	x(196) = 0.2
29	L-threonine	cell	0,177	9	M.U.	x(197) = 0.177
30	NAD	cell	1,5		G.U.	x(217) = 1.5
31	NADH	cell	0,0867		G.U.	x(218) = 0.0867
32	phosphoenolpyruvate	cell	0,153	24	G.U.	x(239) = 0.153
33	pyruvate	cell	0,527	17	G.U.	x(246) = 0.527
34	trehalose	cell	0,00385	20	G.U.	x(261) = 0.00385
35	UDP	cell	0,282		G.U.	x(265) = 0.282
36	UDP-D-glucose	cell	0,467		G.U.	x(266) = 0.467
37	UTP	cell	0,649		G.U.	x(268) = 0.649
38	xanthosine-5-phosphate	cell	0,05		[4]	x(269) = 0.049

Sources: G.U. (glycolysis, unpublished) / M.U. (Manchester Centre for Integrative Systems Biology, unpublished) / [4].

Table S13. Benchmark 1: six experimental flux exchanges

observable number	name	flux amol/s/cell	mM/s	SEM %	state #
39	D-glucose	-74,5	-3,72	32	r_1166: glucose transport
40	carbon dioxide	122	6,11	19	r_1697: CO2 transport
41	ethanol	91,2	4,56	40	r_1762: ethanol transport
42	acetate	1,59	0,0793	28	r_1106: acetate transport
43	glycerol	0,623	0,0311	19	r_1172: glycerol transport
44	trehalose	0,156	0,00778	19	r_2079: trehalose transporter

Six experimental flux exchanges. In addition, ammonia, oxygen, phosphate and sulphate are consumed at unknown rates. Any other flux exchange is assumed to be negligible. Negative fluxes denote flow into the cell. Concentration flux is calculated using an effective volume of 20 fL.

4.2 B2: dynamic CCM of *E. coli*

Experimental data used, but not published, in [5]. Units: concentrations in mmol/l, time in minutes.

Table S14. Benchmark 2: Time-course concentrations of 9 metabolites

time	PEP	G6P	PYR	F6P	GLCex	G1P	6PG	FDP	GAP
0.15	1.99	4.39	4.07	0.62					
0.3	2.10	4.76	3.71	0.66					
0.45	2.09	4.86	3.19	0.74					
0.6	1.84	4.65	3.57	0.62					
0.8	2.31	4.75	3.14	0.75					
2						1.35			
3.5							1.01		
4							0.92		
4.5								0.19	0.28
5.5	2.76	5.52	2.38	0.92	1.256				
11								0.56	0.32
12	3.05	5.86	3.71	1.15			1.15		
12.25							1.19		
13.5					1.311				
16						0.83			
19						0.83			
20								1.00	0.31
21							1.06		
21.5	2.42	4.39	3.19	0.57					
25.75							1.10		
30							1.05	2.83	0.24
31					1.283	0.78			
31.5	2.23	3.60	5.24	0.46					
32.25							1.08		
57						0.84			
58.5							0.97		
59							1.01		
60								1.50	0.30
61	2.52	3.83	4.47	0.57	0.861				
90	2.81	4.30	3.62	0.57				2.26	0.18
91					0.597				
91.5						0.64			
119.5								2.40	0.22
119.75							0.92		
120.5	2.71	4.05	3.62	0.69					
124							0.89		
150.5						0.74			
151					0.096				
178							0.74		
180							0.88	1.25	0.21
180.5	2.71	3.27	2.86	0.46					
181					0.043				
209							0.80		
212.5					0.051				
239.5								0.07	0.22
241					0.048				
270.5					0.048				
299						0.70			

300								0.02	0.20
300.5	2.70	3.38	2.40	0.46					
301					0.06				

Table S15. Benchmark 2: parameter values

Param. name	Param. #	Param. value
kALDO,dhap	p1	0.088 mM
kALDO,eq	p2	0.144 mM
kALDO,fdp	p3	1.75 mM
kALDO,gap	p4	0.088 mM
kALDO,gap,inh	p5	0.6 mM
KDAHPS,e4p	p6	0.035 mM
KDAHPS,pep	p7	0.0053 mM
KENO,eq	p8	6.73 mM
KENO,pg2	p9	0.1 mM
KENO,pep	p10	0.135 mM
KG1PAT,atp	p11	4.42 mM
KG1PAT,fdp	p12	0.119 mM
KG1PAT,g1p	p13	3.2 mM
KG3PDH,dhap	p14	1.0 mM
KG6PDH,g6p	p15	14.4 mM
KG6PDH,nadp	p16	0.0246 mM
KG6PDH,nadph,g6pinh	p17	6.43 mM
KG6PDH,nadph,nadpinh	p18	0.01 mM
KGAPDH,eq	p19	0.63
KGAPDH,gap	p20	0.683 mM
KGAPDH,nad	p21	0.252 mM
KGAPDH,nadh	p22	1.09 mM
KGAPDH,pgp	p23	$1.04 \cdot 10^{-5}$ mM
KPDH,pyr	p24	1159.0 mM
KpepCxylase,fdp	p25	0.7 mM
KpepCxylase,pep	p26	4.07 mM
KPFK,adp,a	p27	128.0 mM
KPFK,adp,b	p28	3.89 mM
KPFK,adpc	p29	4.14 mM
KPFK,amp,a	p30	19.1 mM
KPFK,amp,b	p31	3.2 mM
KPFK,atps	p32	0.123 mM
KPFK,f6ps	p33	0.325 mM
KPFK,pep	p34	3.26 mM
KPGDH,atpinh	p35	208.0 mM
KPGDH,nadp	p36	0.0506 mM
KPGDH,nadphinh	p37	0.0138 mM
KPGDH,pg	p38	37.5 mM
KPGI,eq	p39	0.1725
KPGI,f6p	p40	0.266 mN
KPGI,f6p,pginh	p41	0.2 mM
KPGI,g6p	p42	2.9 mM
KPGI,g6p,pginh	p43	0.2 mM
KPGK,adp	p44	0.185 mM
KPGK,atp	p45	0.653 mM
KPGK,eq	p46	1934.4
KPGK,pg3	p47	0.473 mM
KPGK,pgp	p48	0.0468 mM
KPGluMu,eq	p49	0.188
KPGluMu,pg2	p50	0.369 mM
KPGluMu,pg3	p51	0.2 mM

KPGM,eq	p52	0.196
KPGM,g1p	p53	0.0136 mM
KPGM,g6p	p54	1.038 mM
KPK,adp	p55	0.26 mM
KPK,amp	p56	0.2 mM
KPK,atp	p57	22.5 mM
KPK,fdp	p58	0.19 mM
KPK,pep	p59	0.31 mM
KPTS,a1	p60	3082.3 mM
KPTS,a2	p61	0.01 mM
KPTS,a3	p62	245.3
KPTS,g6p	p63	2.15 mM
KR5PI,eq	p64	4.0
KRPPK,rib5p	p65	0.1 mM
KRu5P,eq	p66	1.4
KSerSynth,pg3	p67	1.0 mM
KSynth1,pep	p68	1.0 mM
KSynth2,pyr	p69	1.0 mM
KTA,eq	p70	1.05
kTIS,dhap	p71	2.8 mM
kTIS,eq	p72	1.39
kTIS,gap	p73	0.3 mM
KTKa,eq	p74	1.2
KTKb,eq	p75	10.0
LPFK	p76	5629067.0
LPK	p77	1000.0
nDAHPS,e4p	p78	2.6
nDAHPS,pep	p79	2.2
nG1PAT,fdp	p80	1.2
nPDH	p81	3.68
npepCxylase,fdp	p82	4.21
nPFK	p83	11.1
nPK	p84	4.0
nPTS,g6p	p85	3.66
rmax,ALDO	p86	17.41464425 mM$\cdot s^{-1}$
rmax,DAHPS	p87	0.1079531227 mM$\cdot s^{-1}$
rmax,ENO	p88	330.4476151 mM$\cdot s^{-1}$
rmax,G1PAT	p89	0.007525458026 mM$\cdot s^{-1}$
rmax,G3PDH	p90	0.01162042696 mM$\cdot s^{-1}$
rmax,G6PDH	p91	1.380196955 mM$\cdot s^{-1}$
rmax,GAPDH	p92	921.5942861 mM$\cdot s^{-1}$
rmax,MetSynth	p93	0.0022627 mM$\cdot s^{-1}$
rmax,MurSynth	p94	$4.3711 \cdot 10^{-4}$ mM$\cdot s^{-1}$
rmax,PDH	p95	6.059531017 mM$\cdot s^{-1}$
rmax,pepCxylase	p96	0.1070205858 mM$\cdot s^{-1}$
rmax,PFK	p97	1840.584747 mM$\cdot s^{-1}$
rmax,PGDH	p98	16.23235977 mM$\cdot s^{-1}$
rmax,PGI	p99	650.9878687 mM$\cdot s^{-1}$
rmax,PGK	p100	3021.773771 mM$\cdot s^{-1}$
rmax,PGluMu	p101	89.04965407 mM$\cdot s^{-1}$
rmax,PGM	p102	0.8398242773 mM$\cdot s^{-1}$
rmax,PK	p103	0.06113150238 mM$\cdot s^{-1}$
rmax,PTS	p104	7829.78 mM$\cdot s^{-1}$
rmax,R5PI	p105	4.83841193 mM$\cdot s^{-1}$

rmax,RPPK	p106	0.01290045226 mM·s^{-1}
rmax,Ru5P	p107	6.739029475 mM·s^{-1}
rmax,SerSynth	p108	0.025712107 mM·s^{-1}
rmax,Synth1	p109	0.01953897003 mM·s^{-1}
rmax,Synth2	p110	0.07361855055 mM·s^{-1}
rmax,TA	p111	10.87164108 mM·s^{-1}
rmax,TIS	p112	68.67474392 mM·s^{-1}
rmax,TKa	p113	9.473384783 mM·s^{-1}
rmax,TKb	p114	86.55855855 mM·s^{-1}
rmax,TrpSynth	p115	0.001037 mM·s^{-1}
VALDO,blf	p116	2.0

Table S16. Benchmark 2: model outputs

Number	Metabolite	Number	Metabolite
1	DHAP	10	2PG
2	E4P	11	3PG
3	F6P	12	PGP
4	FDP	13	PYR
5	GLP	14	RIB5P
6	G6P	15	RIBU5P
7	GAP	16	SED7P
8	PEP	17	XYL5P
9	6PG	18	Extrac. GLC

4.3 B3: regulation of the CCM of *E. coli*

Table S17. Benchmark 3: parameter values

Param. number	Param. name	Description	Param. value
1	$p_{ENV,M_{ACT}}$	Molar mass of acetate	60.05 $g_{ACT}mol^{-1}$
2	$p_{ENV,M_{GLC}}$	Molar mass of glucose	180.156 $g_{GLC}mol^{-1}$
3	$p_{ENV,UC}$	Unit conversion	$9.5 \cdot 10^{-7} g_{DW}(\mu[OD])^{-1}$
4	$p_{AceA,K_{cat}}$	Specific activity	614 $\mu mol(g_{prots})^{-1}$
5	$p_{AceA,n}$	Number of subunits	4
6	$p_{AceA,L}$	Allosteric constant	$5.01 \cdot 10^4$
7	$p_{AceA,K_{ICT}}$	Affinity constant	0.022 $\mu mol g_{DW}^{-1}$
8	$p_{AceA,K_{PEP}}$	Affinity constant	0.055 $\mu mol g_{DW}^{-1}$
9	$p_{AceA,K_{PG3}}$	Affinity constant	0.72 $\mu mol g_{DW}^{-1}$
10	$p_{AceA,K_{AKG}}$	Affinity constant	0.827 $\mu mol g_{DW}^{-1}$
11	$p_{AceB,K_{cat}}$	Specific activity	47.8 $\mu mol(g_{Prot}s)^{-1}$
12	$p_{AceB,K_{GLX}}$	Affinity constant	0.95 $\mu mol g_{DW}^{-1}$
13	$p_{AceB,K_{ACoA}}$	Affinity constant	0.755 $\mu mol g_{DW}^{-1}$
14	$p_{AceB,K_{GLXACoA}}$	Affinity constant	0.719 $\mu mol g_{DW}^{-1}$
15	$p_{AceK,K_{cat,ki}}$	Specific activity	$3.4 \cdot 10^{12}$ s^{-1}
16	$p_{AceK,K_{cat,ph}}$	Specific activity	$1.7 \cdot 10^{9}$ s^{-1}
17	$p_{AceK,n}$	Number of subunits	2
18	$p_{AceK,L}$	Allosteric constant	$1 \cdot 10^8$
19	$p_{AceK,K_{Icd}}$	Affinity constant	0.043 $g_{prot}g_{DW}^{-1}$
20	$p_{AceK,K_{Icd-P}}$	Affinity constant	0.643 $g_{prot}g_{DW}^{-1}$
21	$p_{AceK,K_{PEP}}$	Affinity constant	0.539 $\mu mol g_{DW}^{-1}$
22	$p_{AceK,K_{PYR}}$	Affinity constant	0.038 $\mu mol g_{DW}^{-1}$
23	$p_{AceK,K_{OAA}}$	Affinity constant	0.173 $\mu mol g_{DW}^{-1}$
24	$p_{AceK,K_{GLX}}$	Affinity constant	0.866 $\mu mol g_{DW}^{-1}$
25	$p_{AceA,K_{AKG}}$	Affinity constant	0.82 $\mu mol g_{DW}^{-1}$
26	$p_{AceK,K_{PG3}}$	Affinity constant	1.57 $\mu mol g_{DW}^{-1}$
27	$p_{AceK,K_{ICT}}$	Affinity constant	0.137 $\mu mol g_{DW}^{-1}$
28	$p_{Acoa2act,K_{cat}}$	Specific activity	3079 $\mu mol(g_{Prot}s)^{-1}$
29	$p_{Acoa2act,n}$	Number of subunits	2
30	$p_{Acoa2act,L}$	Allosteric constant	$6.39 \cdot 10^5$
31	$p_{Acoa2act,K_{ACoA}}$	Affinity constant	0.022 $\mu mol g_{DW}^{-1}$
32	$p_{Acoa2act,K_{PYR}}$	Affinity constant	0.022 $\mu mol g_{DW}^{-1}$
33	$p_{Acs,k_{cat}}$	Specific activity	$1.0296 \cdot 10^4 \mu mol(g_{Prots})^{-1}$
34	$p_{Acs,K_{ACT}}$	Affinity constant	$10^{-3} g_{ACT}l^{-1}$
35	$p_{Akg2mal,k_{cat}}$	Specific activity	1530 $\mu mol(g_{Prots})^{-1}$
36	$p_{Akg2mal,k_{AKG}}$	Affinity constant	0.548 $\mu mol g_{DW}^{-1}$
37	$p_{CAMP_{degr},k_{cat}}$	Specific activity	$1 \cdot 10^3 \mu mol(g_{Prots})^{-1}$
38	$p_{CAMP_{degr},k_{cCAMP}}$	Affinity constant	0.1 $\mu mol g_{DW}^{-1}$
39	$p_{Cya,k_{cat}}$	Specific activity	993 $\mu mol(g_{Prots})^{-1}$
40	$p_{Cya,k_{EIIA-P}}$	Affinity constant	$1.7 \cdot 10^{-3} g_{Prot}g_{DW}^{-1}$
41	$p_{Emp,k_{cat},f}$	Spec. activ. forward react.	1011 $\mu mol(g_{Prots})^{-1}$
42	$p_{Emp,k_{cat},r}$	Spec. activ. reverse react.	857.4234 $\mu mol(g_{Prots})^{-1}$
43	$p_{Emp,K_{FBP}}$	Affinity constant	5.92 $\mu mol g_{DW}^{-1}$
44	$p_{Emp,K_{PG3}}$	Affinity constant	16.6 $\mu mol g_{DW}^{-1}$
45	$p_{Eno,k_{cat},f}$	Spec. activ. forward react.	704.9945 $\mu mol(g_{Prots})^{-1}$
46	$p_{Eno,k_{cat},r}$	Spec. activ. reverse react.	529.5067 $\mu mol(g_{Prots})^{-1}$
47	$p_{Eno,K_{PG3}}$	Affinity constant	4.76 $\mu mol g_{DW}^{-1}$

48	$p_{Eno,K_{PEP}}$	Affinity constant	1.11 $\mu mol g_{DW}^{-1}$
49	$p_{Fdp,k_{cat}}$	Specific activity	404.2035 $\mu mol(g_{Prots})^{-1}$
50	$p_{Fdp,n}$	Number of subunits	4
51	$p_{Fdp,L}$	Allosteric constant	$4 \cdot 10^6$
52	$p_{Fdp,K_{FBP}}$	Affinity constant	$3 \cdot 10^{-3} \mu mol g_{DW}^{-1}$
53	$p_{Fdp,K_{PEP}}$	Affinity constant	$0.3 \mu mol g_{DW}^{-1}$
54	$p_{GltA,K_{cat}}$	Specific activity	$5.6761 \cdot 10^3 \mu mol(g_{Prots})^{-1}$
55	$p_{GltA,K_{OAA}}$	Affinity constant	$0.029 \mu mol g_{DW}^{-1}$
56	$p_{GltA,K_{ACoA}}$	Affinity constant	$0.212 \mu mol g_{DW}^{-1}$
57	$p_{GltA,K_{OAAACoA}}$	Affinity constant	$0.029 \mu mol g_{DW}^{-1}$
58	$p_{GltA,K_{AKG}}$	Affinity constant	$0.63 \mu mol g_{DW}^{-1}$
59	$p_{Icd,k_{cat}}$	Specific activity	$695 \mu mol(g_{Prots})^{-1}$
60	$p_{Icd,n}$	Number of subunits	2
61	$p_{Icd,L}$	Allosteric constant	127
62	$p_{Icd,K_{ICT}}$	Affinity constant	$1.6 \cdot 10^{-4} \mu mol g_{DW}^{-1}$
63	$p_{Icd,K_{PEP}}$	Affinity constant	$0.334 \mu mol g_{DW}^{-1}$
64	$p_{Mdh,k_{cat}}$	Specific activity	$5.4375 \cdot 10^3 \mu mol(g_{Prots})^{-1}$
65	$p_{Mdh,n}$	Hill coefficient	1.7
66	$p_{Mdh,k_{MAL}}$	Affinity constant	$10.1 \mu mol g_{DW}^{-1}$
67	$p_{MaeAB,k_{cat}}$	Specific activity	1879 $\mu mol(g_{Prots})^{-1}$
68	$p_{MaeAB,n}$	Number of subunits	1.33
69	$p_{MaeAB,L}$	Allosteric constant	$1.04 \cdot 10^5$
70	$p_{MaeAB,K_{MAL}}$	Affinity constant	$6.24 \cdot 10^{-03} \mu mol g_{DW}^{-1}$
71	$p_{MaeAB,k_{ACoA}}$	Affinity constant	$3.64 \mu mol g_{DW}^{-1}$
72	$p_{MaeAB,k_{CAMP}}$	Affinity constant	$6.54 \mu mol g_{DW}^{-1}$
73	$p_{PckA,k_{cat}}$	Specific activity	$377.3427 \mu mol(g_{Prots})^{-1}$
74	$p_{PckA,k_{OAA}}$	Affinity constant	$0.184 \mu mol g_{DW}^{-1}$
75	$p_{PckA,k_{PEP}}$	Affinity constant	$1000 \mu mol g_{DW}^{-1}$
76	$p_{Pdh,k_{cat}}$	Specific activity	$5.4793 \cdot 10^3 \mu mol(g_{Prots})^{-1}$
77	$p_{Pdh,n}$	Number of subunits	2.65
78	$p_{Pdh,L}$	Allosteric constant	3.4
79	$p_{Pdh,K_{PYR}}$	Affinity constant	$0.128 \mu mol g_{DW}^{-1}$
80	$p_{Pdh,K_{I,PYR}}$	Affinity constant	$0.231 \mu mol g_{DW}^{-1}$
81	$p_{Pdh,K_{GLX}}$	Affinity constant	$0.218 \mu mol g_{DW}^{-1}$
82	$p_{PfkA,K_{cat}}$	Specific activity	$5.3932 \cdot 10^5 \mu mol(g_{Prots})^{-1}$
83	$p_{PfkA,n}$	Number of subunits	4
84	$p_{PfkA,L}$	Allosteric constant	$9.5 \cdot 10^7$
85	$p_{PfkA,K_{G6P}}$	Affinity constant	$0.022 \mu mol g_{DW}^{-1}$
86	$p_{PfkA,K_{PEP}}$	Affinity constant	$0.138 \mu mol g_{DW}^{-1}$
87	$p_{Ppc,K_{cat}}$	Specific activity	$1.4905 \cdot 10^4 \mu mol(g_{Prots})^{-1}$
88	$p_{Ppc,n}$	Number of subunits	3
89	$p_{Ppc,L}$	Allosteric constant	$5.2 \cdot 10^6$
90	$p_{Ppc,K_{PEP}}$	Affinity constant	$0.048 \mu mol g_{DW}^{-1}$
91	$p_{Ppc,K_{FBP}}$	Affinity constant	$0.408 \mu mol g_{DW}^{-1}$
92	$p_{PpsA,k_{cat}}$	Specific activity	$1.32 \mu mol(g_{Prots})^{-1}$
93	$p_{P_{psA},n}$	Number of subunits	2
94	$p_{P_{psA},L}$	Allosteric constant	$1.0 \cdot 10^{-79}$
95	$p_{P_{psA},K_{PYR}}$	Affinity constant	$1.77 \cdot 10^{-3} \mu mol g_{DW}^{-1}$
96	$p_{P_{psA},K_{PEP}}$	Affinity constant	$1.0 \cdot 10^{-3} \mu mol g_{DW}^{-1}$
97	$p_{P_{PykF},K_{cat}}$	Specific activity	$1.3735 \cdot 10^4 \mu mol(g_{Prots})^{-1}$
98	$p_{P_{PykF},n}$	Number of subunits	4
99	$p_{P_{PykF},L}$	Allosteric constant	$1.0 \cdot 10^4$
100	$p_{P_{PykF},K_{PEP}}$	Affinity constant	$5 \mu mol g_{DW}^{-1}$
101	$p_{P_{PykF},K_{FBP}}$	Affinity constant	$0.413 \mu mol g_{DW}^{-1}$

102	p_{P_{PTS},K_l}	Specific activity	$116\mu mol(g_{Prots})^{-1}$
103	$p_{P_{PTS},K_{m_1}}$	Specific activity	$46.3\mu mol(g_{Prots})^{-1}$
104	p_{P_{PTS},K_4}	Specific activity	$2520\mu mol(g_{Prots})^{-1}$
105	$p_{P_{PTS},K_{EIIA}}$	Affinity constant	$8.5\cdot 10^{-3}g_{Prot}g_{DW}^{-1}$
106	$p_{P_{PTS},K_{GLC}}$	Affinity constant	$1.2\cdot 10^{-3}g_{GLC}l^{-1}$
107	$p_{P_{Cra},scale}$	Specific activity	$100g_{Prot}(\mu mols)^{-1}$
108	$p_{P_{Cra},K_{FBP}}$	Affinity constant	$1.36\mu mol g_{DW}^{-1}$
109	$p_{P_{Cra},n}$	Hill coefficient	2
110	$p_{P_{Crp},scale}$	Specific activity	$1.0\cdot 10^{8}g_{Prot}(\mu mols)^{-1}$
111	$p_{P_{Crp},K_{cAMP}}$	Affinity constant	$0.895\mu mol g_{DW}^{-1}$
112	$p_{P_{Crp},n}$	Hill coefficient	1
113	$p_{P_{PdhR},scale}$	Specific activity	$100g_{Prot}(\mu mols)^{-1}$
114	$p_{P_{PdhR},K_{PYR}}$	Affinity constant	$0.164\mu mol g_{DW}^{-1}$
115	$p_{P_{PdhR},n}$	Hill coefficient	1
116	$p_{aceBAK,v_{Cra},unbound}$	Basal expression rate	$1.9\cdot 10^{-9}g_{Prot}(g_{DW}s)^{-1}$
117	$p_{aceBAK,v_{Cra},bound}$	Max. expression rate	$2.0\cdot 10^{-6}g_{Prot}(g_{DW}s)^{-1}$
118	$p_{P_{aceBAK},K_{Cra}}$	Affinity constant	$3.65\cdot 10^{-3}g_{Prot}g_{DW}^{-1}$
119	$p_{aceBAK,aceBfactor}$	Scaling factor	0.3
120	$p_{aceBAK,aceKfactor}$	Scaling factor	0.03
121	$p_{aceBAK,K_{DNA}}$	Affinity constant	2.19 [AU] g_{DW}^{-1}
122	$p_{aceBAK,K_{PYR}}$	Affinity constant	$0.897\mu mol g_{DW}^{-1}$
123	$p_{aceBAK,K_{PYRprime}}$	Affinity constant	$3.01\cdot 10^{-3}\mu mol g_{DW}^{-1}$
124	$p_{aceBAK,K_{GLX}}$	Affinity constant	$4.88\cdot 10^{-3}\mu mol g_{DW}^{-1}$
125	$p_{aceBAK,L}$	Allosteric constant	923
126	$p_{aceBAK,K_{cat,IclR}}$	Specific activity	$9.3\cdot 10^{-4}s^{-1}$
127	$p_{aceBAK,DNA}$	DNA concentration	1 [AU]g_{DW}^{-1}
128	$p_{aceBAK,v_{Crp,bound}}$	Basal expression rate	$2.3\cdot 10^{-10}g_{Prot}(g_{DW}s)^{-1}$
129	$p_{aceBAK,v_{Crp,unbound}}$	Max. expression rate	$2.0\cdot 10^{-8}g_{Prot}(g_{DW}s)^{-1}$
130	$p_{aceBAK,K_{Crp}}$	Affinity constant	$0.341\ g_{Prot}(g_{DW}s)^{-1}$
131	$p_{acs,v_{Crp,unbound}}$	Basal expression rate	$0\ g_{Prot}(g_{DW}s)^{-1}$
132	$p_{acs,v_{Crp,bound}}$	Max. expression rate	$3.9628\cdot 10^{-8}g_{Prot}(g_{DW}s)^{-1}$
133	$p_{acs,n}$	Hill coefficient	2.31
134	$p_{acs,K_{Crp}}$	Affinity constant	$4.7\cdot 10^{-3}g_{Prot}g_{DW}^{-1}$
135	$p_{akg2mal,v_{Crp,unbound}}$	Basal expression rate	$0\ g_{Prot}(g_{DW}s)^{-1}$
136	$p_{akg2mal,v_{Crp,bound}}$	Max. expression rate	$1.4\cdot 10^{-6}g_{Prot}(g_{DW}s)^{-1}$
137	$p_{akg2mal,K_{Crp}}$	Affinity constant	$0.091\ g_{Prot}g_{DW}^{-1}$
138	$p_{akg2mal,n}$	Hill coefficient	0.74
139	$p_{emp,v_{Cra},unbound}$	Max. expression rate	$6.1319\cdot 10^{-7}g_{Prot}(g_{DW}s)^{-1}$
140	$p_{emp,v_{Cra},unbound}$	Basal expression rate	$0\ g_{Prot}(g_{DW}s)^{-1}$
141	$p_{emp,K_{Cra}}$	Affinity constant	$0.09\ g_{Prot}g_{DW}^{-1}$
142	$p_{emp,v_{Crp,unbound}}$	Basal expression rate	$0\ g_{Prot}(g_{DW}s)^{-1}$
143	$p_{emp,v_{Crp,bound}}$	Max. expression rate	$4.7\cdot 10^{-7}g_{Prot}(g_{DW}s)^{-1}$
144	$p_{emp,K_{Crp}}$	Affinity constant	$0.012\ g_{Prot}g_{DW}^{-1}$
145	$p_{eno,v_{Cra,unbound}}$	Max. expression rate	$6.7036\cdot 10^{-7}g_{Prot}(g_{DW}s)^{-1}$
146	$p_{eno,v_{Cra,bound}}$	Basal expression rate	$0\ g_{Prot}(g_{DW}s)^{-1}$
147	$p_{eno,K_{Cra}}$	Affinity constant	$0.016\ g_{Prot}g_{DW}^{-1}$
148	$p_{fdp,v_{Cra,unbound}}$	Basal expression rate	$0\ g_{Prot}(g_{DW}s)^{-1}$
149	$p_{fdp,v_{Cra,bound}}$	Max. expression rate	$2.1375\cdot 10^{-8}g_{Prot}(g_{DW}s)^{-1}$
150	$p_{fdp,K_{Cra}}$	Affinity constant	$1.18\cdot 10^{-3}g_{Prot}g_{DW}^{-1}$
151	$p_{gltA,v_{Crp,unbound}}$	Basal expression rate	$0\ g_{Prot}(g_{DW}s)^{-1}$
152	$p_{gltA,v_{Crp,bound}}$	Max. expression rate	$6.5401\cdot 10^{-7}g_{Prot}(g_{DW}s)^{-1}$
153	$p_{gltA,K_{Crp}}$	Affinity constant	$0.04\ g_{Prot}g_{DW}^{-1}$
154	$p_{gltA,n}$	Hill coefficient	1.07
155	$p_{icd,v_{Cra,unbound}}$	Basal expression rate	$1.1\cdot 10^{-7}g_{Prot}(g_{DW}s)^{-1}$

156	$p_{icd,v_{Cra,bound}}$	Max. expression rate	$8.5 \cdot 10^{-7} g_{Prot}(g_{DW}s)^{-1}$
157	$p_{icd,K_{Cra}}$	Affinity constant	$1.17 \cdot 10^{-3} g_{Prot}g_{DW}^{-1}$
158	$p_{mdh,v_{Crp,unbound}}$	Basal expression rate	$0\ g_{Prot}(g_{DW}s)^{-1}$
159	$p_{mdh,v_{Crp,bound}}$	Max. expression rate	$1.2937 \cdot 10^{-6} g_{Prot}(g_{DW}s)^{-1}$
160	$p_{mdh,K_{Crp}}$	Affinity constant	$0.06\ g_{Prot}g_{DW}^{-1}$
161	$p_{pckA,v_{Cra,unbound}}$	Basal expression rate	$0\ g_{Prot}g_{DW}^{-1}$
162	$p_{pckA,v_{Cra,bound}}$	Max. expression rate	$3.6770 \cdot 10^{-7} g_{Prot}(g_{DW}s)^{-1}$
163	$p_{pckA,K_{Cra}}$	Affinity constant	$5.35 \cdot 10^{-3} g_{Prot}g_{DW}^{-1}$
164	$p_{pdh,v_{PdhR,unbound}}$	Max. expression rate	$7.7463 \cdot 10^{-8} g_{Prot}(g_{DW}s)^{-1}$
165	$p_{pdh,v_{PdhR,bound}}$	Basal expression rate	$2.7973 \cdot 10^{-7} g_{Prot}(g_{DW}s)^{-1}$
166	$p_{pdh,K_{PdhR}}$	Affinity constant	$3.4 \cdot 10^{-3} g_{Prot}g_{DW}^{-1}$
167	$p_{pfkA,v_{Cra,unbound}}$	Max. expression rate	$1.3806 \cdot 10^{-6} g_{Prot}(g_{DW}s)^{-1}$
168	$p_{pfkA,v_{Cra,bound}}$	Basal expression rate	$1.1112 \cdot 10^{-8} g_{Prot}(g_{DW}s)^{-1}$
169	$p_{pfkA,K_{Cra}}$	Affinity constant	$6.3 \cdot 10^{-7} g_{Prot}g_{DW}^{-1}$
170	$p_{ppsA,v_{Cra,unbound}}$	Basal expression rate	$0\ g_{Prot}(g_{DW}s)^{-1}$
171	$p_{ppsA,v_{Cra,unbound}}$	Max. expression rate	$3.3 \cdot 10^{-6} g_{Prot}(g_{DW}s)^{-1}$
172	$p_{ppsA,K_{Cra}}$	Affinity constant	$0.017\ g_{Prot}g_{DW}^{-1}$
173	$p_{pykF,v_{Cra,unbound}}$	Max. expression rate	$1.6324 \cdot 10^{-7} g_{Prot}(g_{DW}s)^{-1}$
174	$p_{pykF,v_{Cra,bound}}$	Basal expression rate	$8.7901 \cdot 10^{-10} g_{Prot}(g_{DW}s)^{-1}$
175	$p_{pykF,K_{Cra}}$	Affinity constant	$2.3 \cdot 10^{-3} g_{Prot}g_{DW}^{-1}$
176	$p_{D,K_{degr}}$	Univ. prot. degr. rate	$2.8 \cdot 10^{-5}\ s^{-1}$
177	$p_{BM,K_{expr}}$	Gene expr. rate const.	$2.0 \cdot 10^{4}\ s$
178	$p_{BM,\mu_{ACT}}$	Growth rate on acetate	$5.6 \cdot 10^{-5}\ s^{-1}$
179	$p_{BM,\mu_{GLC}}$	Growth rate on glucose	$1.8 \cdot 10^{-4}\ s^{-1}$
180	$p_{BM,GLC_{ACoA}}$	1st order rate constant	$1.88\ s^{-1}$
181	$p_{BM,GLC_{AKG}}$	1st order rate constant	$0.978\ s^{-1}$
182	$p_{BM,GLC_{G6P}}$	1st order rate constant	$0.154\ s^{-1}$
183	$p_{BM,GLC_{OAA}}$	1st order rate constant	$6.4\ s^{-1}$
184	$p_{BM,GLC_{PEP}}$	1st order rate constant	$0.423\ s^{-1}$
185	$p_{BM,GLC_{PG3}}$	1st order rate constant	$0.049\ s^{-1}$
186	$p_{BM,GLC_{PYR}}$	1st order rate constant	$0.553\ s^{-1}$
187	$p_{BM,ACT_{ACoA}}$	1st order rate constant	$0.108\ s^{-1}$
188	$p_{BM,ACT_{AKG}}$	1st order rate constant	$0.056\ s^{-1}$
189	$p_{BM,ACT_{G6P}}$	1st order rate constant	$0.076\ s^{-1}$
190	$p_{BM,ACT_{OOA}}$	1st order rate constant	$1.43\ s^{-1}$
191	$p_{BM,ACT_{PEP}}$	1st order rate constant	$0.047\ s^{-1}$
192	$p_{BM,ACT_{PG3}}$	1st order rate constant	$0.066\ s^{-1}$
193	$p_{BM,ACT_{PYR}}$	1st order rate constant	$5.185\ s^{-1}$

Table S18. Benchmark 3: model outputs (states)

Number	Name	Description
1	xOD	Biomass concentration
2	xxACT	Extracellular acetate
3	xxGLC	Extracellular glucose
4	xxACoA	Acetyl-CoA
5	xxAKG	α-Ketoglutarate
6	xxcAMP	Cyclic AMP
7	xxFBP	Fructose-1,6-bisphosphate
8	xxG6P	Glucose-6-phosphate
9	xxGLX	Glyoxylate
10	xxICT	Isocitrate
11	xxMAL	Malate
12	xxOAA	Oxaloacetate
13	xxPEP	Phosphoenolpyruvate
14	xxPG3	3-Phosphoglycerate
15	xxPY	R Pyruvate
16	xxAceA	Isocitrate lyase
17	xxAceB	Malate synthase A
18	xxAceK	Isocitrate dehydrogenase phosphatase/kinase
19	xxAcoa2act	Enzyme for the reaction from ACoA to ACT
20	xxAcs	Acetyl-CoA synthetase
21	xxAkg2mal	Enzyme for the reaction from AKG to MAL
22	xxCAMPdegr	Degradation of cAMP
23	xxCya	Adenylate cyclase
24	xxEmp	Enzyme for the reversible reaction between FBP and PG3
25	xxEno	Enolase
26	xxFdp	Fructose-1,6-bisphosphatase I
27	xxGltA	Citrate synthase
28	xxIcd	Unphosphorylated isocitrate dehydrogenase
29	xxIcd-P	Phosphorylated isocitrate dehydrogenase
30	xxMaeAB	Malic enzymes MaeAB
31	xxMdh	Malate dehydrogenase
32	xxPckA	Phosphoenolpyruvate carboxykinase
33	xxPdh	Pyruvate dehydrogenase
34	xxPfkA	6-phosphofructokinase I
35	xxPpc	Phosphoenolpyruvate carboxylase
36	xxPpsA	Phosphoenolpyruvate synthase
37	xxPykF	Pyruvate kinase I
38	xxEIIA	Unphosphorylated PTS protein EIIA
39	xxEIIA-P	Phosphorylated PTS protein EIIA
40	xxEIICB	PTS protein EIICB
41	xxCra	Free Cra
42	xxCraFBP	Cra bound to fructose-1,6-bisphosphate
43	xxCrp	Free Crp
44	xxCrpcAMP	Crp bound to cyclic AMP
45	xxIclR	IclR
46	xxPdhR	free PdhR
47	xxPdhRPYR	PdhR bound to pyruvate

4.4 B4: CHO

Table S19. Benchmark 4: parameter values

Param. number	Parameter name	Param. value
1	Km_Subset4_D-Glycerate_3-phosphate_c	1000 $\mu mol/l$
2	Km_Subset4_NAD_c	1000 $\mu mol/l$
3	Km_Subset4_L-Glutamate_c	1000 $\mu mol/l$
4	Km_Subset4_L-Leucine_c	1000 $\mu mol/l$
5	Km_Subset4_L-Methionine_c	1000 $\mu mol/l$
6	Km_Subset4_L-Aspartate_c	1000 $\mu mol/l$
7	Km_Subset4_L-Glutamine_c	1000 $\mu mol/l$
8	Km_Subset4_ATP_c	1000 $\mu mol/l$
9	e_substrate_Glud1_2-Oxoglutarate_m	1
10	e_substrate_Glud1_NADH_m	1
11	e_product_Glud1_L-Glutamate_m	$-7 \cdot 10^{-1}$
12	e_product_Glud1_NAD_m	$-7 \cdot 10^{-1}$
13	e_substrate_Glul_L-Glutamate_c	$7 \cdot 10^{-1}$
14	e_substrate_Glul_ATP_c	$7 \cdot 10^{-1}$
15	e_product_Glul_L-Glutamine_c	$-2 \cdot 10^{-1}$
16	e_product_Glul_ADP_c	$-2 \cdot 10^{-1}$
17	e_substrate_Got2_Oxaloacetate_m	1
18	e_substrate_Got2_L-Glutamate_m	1
19	e_product_Got2_2-Oxoglutarate_m	$-7 \cdot 10^{-1}$
20	e_product_Got2_L-Aspartate_m	$-7 \cdot 10^{-1}$
21	e_activator_Got2_L-Malate_m	$5 \cdot 10^{-2}$
22	e_substrate_Mdh2_L-Malate_m	1
23	e_substrate_Mdh2_NAD_m	$5 \cdot 10^{-1}$
24	e_product_Mdh2_Oxaloacetate_m	$-7 \cdot 10^{-1}$
25	e_product_Mdh2_NADH_m	$-5 \cdot 10^{-1}$
26	e_substrate_ND1_a_NADH_m	2
27	e_substrate_ND1_a_CoQ_m	$7 \cdot 10^{-1}$
28	e_substrate_ND1_a_H_in_m	$7 \cdot 10^{-1}$
29	e_product_ND1_a_NAD_m	-2
30	e_product_ND1_a_CoQH_radical_m	$-2 \cdot 10^{-1}$
31	e_product_ND1_a_H_out_m	$-2 \cdot 10^{-1}$
32	e_substrate_Pklr_Phosphoenolpyruvate_c	1
33	e_substrate_Pklr_ADP_c	$7 \cdot 10^{-1}$
34	e_product_Pklr_Pyruvate_c	$-2 \cdot 10^{-1}$
35	e_product_Pklr_ATP_c	$-2.1 \cdot 10^{-1}$
36	e_substrate_Subset0_Oxaloacetate_m	1
37	e_substrate_Subset0_NAD_m	$7 \cdot 10^{-1}$
38	e_substrate_Subset0_Pyruvate_c	1
39	e_product_Subset0_2-Oxoglutarate_m	$-2 \cdot 10^{-1}$
40	e_product_Subset0_NADH_m	$-2 \cdot 10^{-1}$
41	e_activator_Subset0_ADP_m	$5 \cdot 10^{-2}$
42	e_inhibitor_Subset0_ATP_m	$-1 \cdot 10^{-2}$
43	e_substrate_Subset1_NAD_c	$7 \cdot 10^{-1}$
44	e_substrate_Subset1_beta-D-Glucose_c	1
45	e_product_Subset1_NADH_c	$-5 \cdot 10^{-1}$
46	e_product_Subset1_D-Glycerate_3-phosphate_c	-1
47	e_activator_Subset1_ADP_c	$4 \cdot 10^{-2}$
48	e_inhibitor_Subset1_ADP_c	$-1 \cdot 10^{-2}$

49	e_inhibitor_Subset1_ATP_c	$-1 \cdot 10^{-2}$
50	e_inhibitor_Subset1_Phosphoenolpyruvate_c	$-1 \cdot 10^{-2}$
51	e_substrate_Subset2_2-Oxoglutarate_c	1
52	e_substrate_Subset2_L-Aspartate_c	1
53	e_substrate_Subset2_NADH_c	$5 \cdot 10^{-1}$
54	e_product_Subset2_L-Glutamate_c	-1
55	e_product_Subset2_L-Malate_c	-1
56	e_product_Subset2_NAD_c	$-5 \cdot 10^{-1}$
57	e_activator_Subset2_L-Malate_c	$1 \cdot 10^{-1}$
58	e_inhibitor_Subset2_L-Glutamine_c	$-1 \cdot 10^{-1}$
59	e_substrate_Subset26_ADP_m	$5 \cdot 10^{-1}$
60	e_substrate_Subset26_Phosphoenolpyruvate_c	1
61	e_substrate_Subset26_L-Malate_m	1
62	e_product_Subset26_Oxaloacetate_m	-1
63	e_product_Subset26_ATP_m	$-3 \cdot 10^{-1}$
64	e_product_Subset26_L-Malate_c	-1
65	e_substrate_Subset3_Pyruvate_c	1
66	e_substrate_Subset3_NADH_c	$7 \cdot 10^{-1}$
67	e_product_Subset3_L-Lactate_f	$-4 \cdot 10^{-1}$
68	e_product_Subset3_NAD_c	$-7 \cdot 10^{-1}$
69	e_substrate_Subset35_D-Glycerate_3-phosphate_c	1
70	e_product_Subset35_Phosphoenolpyruvate_c	$-7 \cdot 10^{-1}$
71	e_substrate_Subset37_H_in_m	$7 \cdot 10^{-1}$
72	e_substrate_Subset37_CoQH_radical_m	2
73	e_product_Subset37_H_out_m	$-2 \cdot 10^{-1}$
74	e_product_Subset37_CoQ_m	-2
75	e_substrate_Subset5_2-Oxoglutarate_m	1
76	e_substrate_Subset5_NAD_m	$7 \cdot 10^{-1}$
77	e_substrate_Subset5_CoQ_m	$7 \cdot 10^{-1}$
78	e_substrate_Subset5_Orthophosphate_m	$2 \cdot 10^{-1}$
79	e_substrate_Subset5_ADP_m	$7 \cdot 10^{-1}$
80	e_product_Subset5_L-Malate_m	$-5 \cdot 10^{-1}$
81	e_product_Subset5_NADH_m	$-2.1 \cdot 10^{-1}$
82	e_product_Subset5_CoQH_radical_m	$-2 \cdot 10^{-1}$
83	e_product_Subset5_ATP_m	$-2 \cdot 10^{-1}$
84	e_inhibitor_Subset5_Oxaloacetate_m	$-1 \cdot 10^{-2}$
85	e_substrate_adencarr_ADP_c	2
86	e_substrate_adencarr_ATP_m	2
87	e_product_adencarr_ADP_m	-2
88	e_product_adencarr_ATP_c	-2
89	e_substrate_akgcarr_2-Oxoglutarate_m	2
90	e_substrate_akgcarr_L-Malate_c	2
91	e_product_akgcarr_2-Oxoglutarate_c	-2
92	e_product_akgcarr_L-Malate_m	-2
93	e_substrate_aspglucarr_L-Aspartate_m	1
94	e_substrate_aspglucarr_L-Glutamate_c	1
95	e_product_aspglucarr_L-Aspartate_c	-1
96	e_product_aspglucarr_L-Glutamate_m	-1
97	e_substrate_atpase_ATP_c	1
98	e_product_atpase_ADP_c	$-5 \cdot 10^{-1}$
99	e_substrate_atpase1_ADP_m	$7 \cdot 10^{-1}$
100	e_substrate_atpase1_Orthophosphate_m	$7 \cdot 10^{-1}$
101	e_substrate_atpase1_H_out_m	2
102	e_product_atpase1_ATP_m	$-2 \cdot 10^{-1}$

103	e_product_atpase1_H_in_m	-2
104	e_substrate_dicarr_L-Malate_c	1
105	e_substrate_dicarr_Orthophosphate_m	1
106	e_product_dicarr_L-Malate_m	$-7 \cdot 10^{-1}$
107	e_substrate_feed_glc_beta-D-Glucose_f	1
108	e_product_feed_glc_beta-D-Glucose_c	$-7 \cdot 10^{-1}$
109	e_substrate_feed_leu_L-Leucine_f	1
110	e_product_feed_leu_L-Leucine_c	$-7 \cdot 10^{-1}$
111	e_substrate_feed_met_L-Methionine_f	1
112	e_product_feed_met_L-Methionine_c	$-7 \cdot 10^{-1}$
113	e_substrate_glucarr_L-Glutamate_m	2
114	e_product_glucarr_L-Glutamate_c	-2
115	e_substrate_mitphocarr_H_out_m	$7 \cdot 10^{-1}$
116	e_product_mitphocarr_Orthophosphate_m	$-2 \cdot 10^{-1}$
117	e_product_mitphocarr_H_in_m	$-2 \cdot 10^{-1}$

Table S20. Benchmark 4: model outputs (metabolite concentrations)

Number	Metabolite
1	beta-D-Glucose (fermenter)
2	L-Lactate (fermenter)
3	L-Leucine (fermenter)
4	L-Methionine (fermenter)
5	Productprotein (fermenter)
6	L-Glutamate (mitochondria)
7	NAD (mitochondria)
8	2-Oxoglutarate (mitochondria)
9	NADH (mitochondria)
10	L-Glutamine (cytosol)
11	ADP (cytosol)
12	L-Glutamate (cytosol)
13	ATP (cytosol)
14	L-Aspartate (mitochondria)
15	Oxaloacetate (mitochondria)
16	L-Malate (mitochondria)
17	CoQH_radical (mitochondria)
18	H_out (mitochondria)
19	CoQ (mitochondria)
20	H_in (mitochondria)
21	Pyruvate (cytosol)
22	Phosphoenolpyruvate (cytosol)
23	NADH (cytosol)
24	D-Glycerate_3-phosphate (cytosol)
25	NAD (cytosol)
26	beta-D-Glucose (cytosol)
27	L-Malate (cytosol)
28	2-Oxoglutarate (cytosol)
29	L-Aspartate (cytosol)
30	ATP (mitochondria)
31	Orthophosphate (mitochondria)
32	ADP (mitochondria)
33	L-Leucine (cytosol)
34	L-Methionine (cytosol)

4.5 B5: signal transduction logic model

Table S21. Benchmark 5: parameter values

Param. name	Param. #	Param. value
map3k7_n_nik	p1	1.0000
map3k7_k_nik	p2	$9.0500{\cdot}10^{-2}$
tau_nik	p3	7.1398
map3k7_n_mkk4	p4	1.0900
map3k7_k_mkk4	p5	$9.5000{\cdot}10^{-1}$
map3k1_n_mkk4	p6	3.1526
map3k1_k_mkk4	p7	$4.7210{\cdot}10^{-1}$
tau_mkk4	p8	5.4167
traf2_n_ask1	p9	3.4873
traf2_k_ask1	p10	$1.5000{\cdot}10^{-1}$
tau_ask1	p11	5.2731
traf2_n_map3k7	p12	1.0000
traf2_k_map3k7	p13	$9.1800{\cdot}10^{-2}$
tau_map3k7	p14	$1.0000{\cdot}10^{-1}$
ask1_n_mkk7	p15	3.5966
ask1_k_mkk7	p16	$7.7720{\cdot}10^{-1}$
map3k1_n_mkk7	p17	3.9788
map3k1_k_mkk7	p18	$4.0260{\cdot}10^{-1}$
tau_mkk7	p19	$1.0000{\cdot}10^{1}$
tnfa_n_tnfr	p20	1.0188
tnfa_k_tnfr	p21	$1.1730{\cdot}10^{-1}$
tau_tnfr	p22	9.1369
egf_n_egfr	p23	4.8920
egf_k_egfr	p24	$9.0500{\cdot}10^{-2}$
tau_egfr	p25	1.0937
erk_n_ph	p26	4.6110
erk_k_ph	p27	$1.0270{\cdot}10^{-1}$
tau_ph	p28	$1.0600{\cdot}10^{-1}$
nfkb_n_ex	p29	4.9909
nfkb_k_ex	p30	$4.2680{\cdot}10^{-1}$
tau_ex	p31	$6.1460{\cdot}10^{-1}$
raf1_n_mek	p32	1.4875
raf1_k_mek	p33	$5.1950{\cdot}10^{-1}$
tau_mek	p34	9.5228
sos_n_ras	p35	1.4540
sos_k_ras	p36	$7.1190{\cdot}10^{-1}$
tau_ras	p37	2.8385
tnfr_n_traf2	p38	4.1622
tnfr_k_traf2	p39	$5.4760{\cdot}10^{-1}$
tau_traf2	p40	9.9362
nik_n_ikk	p41	4.9970
nik_k_ikk	p42	$1.0290{\cdot}10^{-1}$
tau_ikk	p43	5.7463
pi3k_n_akt	p44	1.0726
pi3k_k_akt	p45	$9.4860{\cdot}10^{-1}$
tau_akt	p46	9.5917
egfr_n_pi3k	p47	4.4151
egfr_k_pi3k	p48	$9.4400{\cdot}10^{-2}$
tau_pi3k	p49	7.7292

ex_n_ikb	p50	4.9991
ex_k_ikb	p51	$6.8520 \cdot 10^{-1}$
ikk_n_ikb	p52	1.5451
ikk_k_ikb	p53	$9.5000 \cdot 10^{-1}$
tau_ikb	p54	$6.7780 \cdot 10^{-1}$
ikb_n_nfkb	p55	4.9997
ikb_k_nfkb	p56	$2.3290 \cdot 10^{-1}$
tau_nfkb	p57	$9.9840 \cdot 10^{-1}$
jnk_n_cjun	p58	1.0759
jnk_k_cjun	p59	$9.4960 \cdot 10^{-1}$
tau_cjun	p60	9.6513
$mkk7\_n\_jnk$	p61	4.8747
$mkk7\_k\_jnk$	p62	$9.1300 \cdot 10^{-2}$
tau_jnk	p63	1.3215
ras_n_map3k1	p64	1.1997
ras_k_map3k1	p65	$8.2980 \cdot 10^{-1}$
tau_map3k1	p66	9.9950
mek_n_erk	p67	2.5354
mek_k_erk	p68	$7.0990 \cdot 10^{-1}$
tau_erk	p69	$1.0000 \cdot 10^{1}$
ras_n_raf1	p70	1.0000
ras_k_raf1	p71	$2.7250 \cdot 10^{-1}$
tau_raf1	p72	$8.5520 \cdot 10^{-1}$
$egfr\_n\_sos$	p73	1.1200
$egfr\_k\_sos$	p74	$9.3750 \cdot 10^{-1}$
ph_n_sos	p75	1.4651
ph_k_sos	p76	$7.2000 \cdot 10^{-1}$
tau_sos	p77	9.3196
$mkk4\_n\_p38$	p78	1.3200
$mkk4\_k\_p38$	p79	$9.3290 \cdot 10^{-1}$
tau_p38	p80	$1.0000 \cdot 10^{-1}$
akt_n_gsk3	p81	1.0608
akt_k_gsk3	p82	$9.0000 \cdot 10^{-2}$
tau_gsk3	p83	$2.6020 \cdot 10^{-1}$
$cjun\_n\_ap1$	p84	4.7985
$cjun\_k\_ap1$	p85	$2.6530 \cdot 10^{-1}$
tau_ap1	p86	$2.0450 \cdot 10^{-1}$

Table S22. Benchmark 5: model outputs

Observable Number	Signals	State Number
1	raf1	19
2	erk	21
3	ap1	26
4	gsk3	25
5	p38	24
6	nfkb	7

4.6 B6: *Drosophila*

Table S23. Benchmark 6: parameter values

Param. name	Description	Param. #	Value
R_1	promoter strength for gap gene 1	p1	12.48991375
R_2	promoter strength for gap gene 2	p2	26.64956536
R_3	promoter strength for gap gene 3	p3	24.21400803
R_4	promoter strength for gap gene 4	p4	19.14696636
$T(1,1)$	effect of gap gene 1 on gap gene 1	p5	-0.02875641
$T(1,2)$	effect of gap gene 1 on gap gene 2	p6	0.03773355
$T(1,3)$	effect of gap gene 1 on gap gene 3	p7	-0.08696411
$T(1,4)$	effect of gap gene 1 on gap gene 4	p8	0.02085833
$T(2,1)$	effect of gap gene 2 on gap gene 1	p9	0.07752513
$T(2,2)$	effect of gap gene 2 on gap gene 2	p10	-0.05127861
$T(2,3)$	effect of gap gene 2 on gap gene 3	p11	0.08351538
$T(2,4)$	effect of gap gene 2 on gap gene 4	p12	-0.03693460
$T(3,1)$	effect of gap gene 3 on gap gene 1	p13	-0.02272315
$T(3,2)$	effect of gap gene 3 on gap gene 2	p14	-0.05630814
$T(3,3)$	effect of gap gene 3 on gap gene 3	p15	0.03840626
$T(3,4)$	effect of gap gene 3 on gap gene 4	p16	0.07150631
$T(4,1)$	effect of gap gene 4 on gap gene 1	p17	0.02276078
$T(4,2)$	effect of gap gene 4 on gap gene 2	p18	-0.02350780
$T(4,3)$	effect of gap gene 4 on gap gene 3	p19	0.01856495
$T(4,4)$	effect of gap gene 4 on gap gene 4	p20	-0.06116937
$E(1,1)$	effect of external gene 1 on gap gene 1	p21	-0.14654266
$E(1,2)$	effect of external gene 1 on gap gene 2	p22	0.01651517
$E(1,3)$	effect of external gene 1 on gap gene 3	p23	0.06442419
$E(1,4)$	effect of external gene 1 on gap gene 4	p24	-0.05467585
$E(2,1)$	effect of external gene 2 on gap gene 1	p25	-0.12247616
$E(2,2)$	effect of external gene 2 on gap gene 2	p26	-0.10550997
$E(2,3)$	effect of external gene 2 on gap gene 3	p27	0.10687539
$E(3,1)$	effect of external gene 3 on gap gene 1	p28	0.02896215
$E(3,2)$	effect of external gene 3 on gap gene 2	p29	-0.07365022
$E(3,3)$	effect of external gene 3 on gap gene 3	p30	-0.06193835
$E(4,1)$	effect of external gene 4 on gap gene 1	p31	0.02710513
$E(4,2)$	effect of external gene 4 on gap gene 2	p32	-0.04491538
$E(4,3)$	effect of external gene 4 on gap gene 3	p33	0.10493181
$lambda_1$	decay rate of gap gene 1	p34	13.76469068
$lambda_2$	decay rate of gap gene 2	p35	7.27890037
$lambda_3$	decay rate of gap gene 3	p36	11.63317492
$lambda_4$	decay rate of gap gene 4	p37	12.03105457

The observables of model 6 are the expression levels of the four gap genes: hunchback (hb), Krüppel (Kr), giant (gt), and knirps (kni). In the data table, horizontal lines divide data obtained at different time points.

Table S24. Benchmark 6: data

lin	t	hb	Kr	gt	kni
4113	10.55	130.56	0	103.74	0
4114	10.55	133.65	0	98.88	0
4115	10.55	136.74	0	78.18	0
4116	10.55	139.42	0	48.74	0
4117	10.55	130.43	0.79	19.75	0
4118	10.55	103.55	16.15	1.85	0
4119	10.55	67.11	49.53	0	0
4120	10.55	30.69	88.23	0	0
4121	10.55	4.62	115.77	0	0
4122	10.55	0	118.49	0	0
4123	10.55	0	115.21	0	2.52
4124	10.55	0	100.6	0	22.34
4125	10.55	0	73.59	0	54.24
4126	10.55	0	42.74	0	85.44
4127	10.55	0	16	0	103.77
4128	10.55	0	1.25	0	103.75
4129	10.55	0	0	0	101.3
4130	10.55	0	0	0.34	96.86
4131	10.55	0	0	8.16	75.51
4132	10.55	0	0	25.8	42.33
4133	10.55	0	0	47.47	12.47
4134	10.55	0	0	67.7	0
4135	10.55	0	0	81.62	0
4136	10.55	0	0	84.11	0
4137	10.55	0	0	81.65	0
4138	10.55	0	0	79.2	0
4139	10.55	0.68	0	76.74	0
8227	24.225	159.89	0	148.7	0
8228	24.225	161.77	0	146.09	0
8229	24.225	163.65	0	134.47	0
8230	24.225	165.54	0	115.97	0
8231	24.225	164.81	0	92.9	0
8232	24.225	158.26	0	67.69	0
8233	24.225	146.76	0.27	42.87	0
8234	24.225	131.29	7.98	21.07	0
8235	24.225	112.88	24.64	5.07	0
8236	24.225	92.59	47.86	0	0
8237	24.225	71.55	75.15	0	0
8238	24.225	50.94	103.91	0	0
8239	24.225	31.96	131.4	0	0
8240	24.225	15.9	154.8	0	0
8241	24.225	4.06	171.16	0	0
8242	24.225	0	173.19	0	0
8243	24.225	0	171.44	0	0
8244	24.225	0	169.69	0	0
8245	24.225	0	167.94	0	3.56
8246	24.225	0	165.75	0	16.68

8247	24.225	0	157.09	0	36.75
8248	24.225	0	141.67	0	60.98
8249	24.225	0	121.48	0	86.72
8250	24.225	0	98.43	0	111.41
8251	24.225	0	74.37	0	132.62
8252	24.225	0	51.05	0	147.99
8253	24.225	0	30.17	0	153.95
8254	24.225	0	13.33	0	152.2
8255	24.225	0	2.05	0	150.45
8256	24.225	0	0	0	148.7
8257	24.225	0	0	0	146.95
8258	24.225	0	0	0.05	145.15
8259	24.225	0	0	6.72	137.66
8260	24.225	0	0	22.25	122.39
8261	24.225	0	0	43.45	101.89
8262	24.225	0	0	67.25	78.62
8263	24.225	0	0	90.76	54.89
8264	24.225	0	0	111.21	32.93
8265	24.225	0	0	125.97	14.83
8266	24.225	0	0	131.2	2.59
8267	24.225	0	0	129.45	0
8268	24.225	0	0	127.7	0
8269	24.225	0	0	125.96	0
8270	24.225	0	0	123.34	0
8271	24.225	0	0	115.11	0
8272	24.225	0	0	101.98	0
8273	24.225	0.01	0	85.57	0
8274	24.225	5.07	0	67.44	0
8275	24.225	17.45	0	49.06	0
8276	24.225	34.6	0	31.81	0
8277	24.225	54.11	0	17	0
8278	24.225	73.71	0	5.85	0
8279	24.225	91.27	0	0	0
8227	30.475	166.81	0	152.75	0
8228	30.475	168.78	0	139.39	0
8229	30.475	170.74	0	120.22	0
8230	30.475	172.7	0	97.19	0
8231	30.475	174.66	0	72.33	0
8232	30.475	176.53	0	47.78	0
8233	30.475	171.77	5.92	25.77	0
8234	30.475	157.92	21.91	8.63	0
8235	30.475	137.12	45.47	0	0
8236	30.475	111.6	73.96	0	0
8237	30.475	83.72	104.64	0	0
8238	30.475	55.92	134.65	0	0
8239	30.475	30.77	160.98	0	0
8240	30.475	10.91	180.53	0	0
8241	30.475	0	187.38	0	0
8242	30.475	0	187.38	0	0
8243	30.475	0	185.48	0	0
8244	30.475	0	183.59	0	0
8245	30.475	0	177.94	0	0
8246	30.475	0	165.41	0	0.89
8247	30.475	0	147.66	0	10.84

8248	30.475	0	126.3	0	29.37
8249	30.475	0	102.89	0	53.53
8250	30.475	0	78.91	0	80.45
8251	30.475	0	55.8	0	107.42
8252	30.475	0	34.91	0	131.81
8253	30.475	0	17.56	0	151.12
8254	30.475	0	4.98	0	162.96
8255	30.475	0	0	0	162.77
8256	30.475	0	0	0	160.88
8257	30.475	0	0	0	158.99
8258	30.475	0	0	0	155.15
8259	30.475	0	0	3.61	144.03
8260	30.475	0	0	19.77	127.14
8261	30.475	0	0	44.54	106.4
8262	30.475	0	0	73.32	83.68
8263	30.475	0	0	101.71	60.75
8264	30.475	0	0	125.53	39.28
8265	30.475	0	0	140.84	20.87
8266	30.475	0	0	141.95	7.04
8267	30.475	0	0	140.06	0
8268	30.475	0	0	136.54	0
8269	30.475	0	0	124.41	0
8270	30.475	3.12	0	105.41	0
8271	30.475	15.3	0	82.44	0
8272	30.475	33.82	0	58.21	0
8273	30.475	55.76	0	35.3	0
8274	30.475	78.35	0	16.17	0
8275	30.475	98.97	0	3.08	0
8276	30.475	115.15	0	0	0
8277	30.475	124.6	0	0	0
8278	30.475	123.64	0	0	0
8279	30.475	121.67	0	0	0
8227	36.725	169.88	0	162.91	0
8228	36.725	171.88	0	146.29	0
8229	36.725	173.88	0	119.87	0
8230	36.725	175.88	0	87.91	0
8231	36.725	177.88	0	54.89	0
8232	36.725	179.88	0	25.49	0
8233	36.725	181.88	0	4.62	0
8234	36.725	183.68	4.55	0	0
8235	36.725	172.74	17.73	0	0
8236	36.725	145.74	37.74	0	0
8237	36.725	108.89	62.74	0	0
8238	36.725	68.7	90.76	0	0
8239	36.725	31.94	119.77	0	0
8240	36.725	5.67	147.64	0	0
8241	36.725	0	172.16	0	0
8242	36.725	0	189.11	0	0
8243	36.725	0	193.68	0	0
8244	36.725	0	191.7	0	0
8245	36.725	0	189.73	0	0
8246	36.725	0	187.75	0	0
8247	36.725	0	185.16	0	6.2
8248	36.725	0	174.21	0	24.21

8249	36.725	0	154.78	0	50.14
8250	36.725	0	129.62	0	80.22
8251	36.725	0	101.34	0	110.81
8252	36.725	0	72.49	0	138.47
8253	36.725	0	45.48	0	159.87
8254	36.725	0	22.61	0	171.86
8255	36.725	0	6.09	0	169.97
8256	36.725	0	0	0	167.99
8257	36.725	0	0	6.06	166.01
8258	36.725	0	0	27.29	163.99
8259	36.725	0	0	57.8	155.19
8260	36.725	0	0	91.55	137.07
8261	36.725	0	0	122.78	112.82
8262	36.725	0	0	146.01	85.46
8263	36.725	0	0	154.15	57.88
8264	36.725	0	0	152.18	32.84
8265	36.725	0	0	150.18	12.94
8266	36.725	0	0	141.92	0.65
8267	36.725	0	0	124.79	0
8268	36.725	0	0	101.91	0
8269	36.725	4.24	0	76.23	0
8270	36.725	17.69	0	50.58	0
8271	36.725	37.32	0	27.61	0
8272	36.725	60.16	0	9.83	0
8273	36.725	83.38	0	0	0
8274	36.725	104.32	0	0	0
8275	36.725	120.48	0	0	0
8276	36.725	129.51	0	0	0
8277	36.725	127.91	0	0	0
8278	36.725	125.91	0	0	0
8279	36.725	123.33	0	0	0
8227	42.975	135.28	0	163.11	0
8228	42.975	136.88	0	145.09	0
8229	42.975	138.47	0	117.9	0
8230	42.975	140.06	0	85.72	0
8231	42.975	141.67	0	52.91	0
8232	42.975	146.31	3.41	24.03	0
8233	42.975	154.88	15.2	3.87	0
8234	42.975	165.49	33.83	0	0
8235	42.975	176.18	57.56	0	0
8236	42.975	184.92	84.55	0	0
8237	42.975	189	112.9	0	0
8238	42.975	149.24	140.61	0	0
8239	42.975	108.12	165.61	0	0
8240	42.975	63.55	185.74	0	0
8241	42.975	24.53	198.03	0	0
8242	42.975	0.45	198.03	0	0
8243	42.975	0	196.03	0	0
8244	42.975	0	194.03	0	0
8245	42.975	0	192.03	0	0.01
8246	42.975	0	185.85	0	7.82
8247	42.975	0	170.45	0	27.3
8248	42.975	0	148.3	0	54.46
8249	42.975	0	121.83	0	85.47

8250	42.975	0	93.41	0	116.65
8251	42.975	0	65.29	0	144.47
8252	42.975	0	39.63	0	165.57
8253	42.975	0	18.49	0	176.03
8254	42.975	0	3.85	0	174.03
8255	42.975	0	0	0	172.03
8256	42.975	0	0	0.18	170.03
8257	42.975	0	0	13.11	158.5
8258	42.975	0	0	40.96	129.76
8259	42.975	0	0	76.28	91.92
8260	42.975	0	0	111.98	52.82
8261	42.975	0	0	141.31	19.94
8262	42.975	0	0	157.81	0.36
8263	42.975	0	0	156.03	0
8264	42.975	0	0	154.03	0
8265	42.975	0	0	152.03	0
8266	42.975	0	0	147.8	0
8267	42.975	0	0	132.32	0
8268	42.975	2.96	0	108.32	0
8269	42.975	17.64	0	79.95	0
8270	42.975	40.55	0	51.14	0
8271	42.975	67.3	0	25.61	0
8272	42.975	93.75	0	6.88	0
8273	42.975	115.95	0	0	0
8274	42.975	130.24	0	0	0
8275	42.975	131.31	0	0	0
8276	42.975	129.31	0	0	0
8277	42.975	127.33	0	0	0
8278	42.975	123.2	0	0	0
8279	42.975	100.41	0	0	0
8227	49.225	131.58	0	165.83	0
8228	49.225	133.13	0	150.93	0
8229	49.225	134.68	0	121.2	0
8230	49.225	136.23	0	83.58	0
8231	49.225	137.77	0	45.38	0
8232	49.225	145.57	0	14.22	0
8233	49.225	161.4	6.26	0	0
8234	49.225	175.83	25.26	0	0
8235	49.225	179.96	53.47	0	0
8236	49.225	181.89	87.1	0	0
8237	49.225	156.53	122.24	0	0
8238	49.225	91.78	154.75	0	0
8239	49.225	25.5	180.36	0	0
8240	49.225	0	192.54	0	0
8241	49.225	0	194.51	0	0
8242	49.225	0	194.51	0	0
8243	49.225	0	192.54	0	0
8244	49.225	0	190.58	0	0
8245	49.225	0	185.84	0	5.39
8246	49.225	0	170.66	0	23.81
8247	49.225	0	147.39	0	51.07
8248	49.225	0	119.09	0	82.9
8249	49.225	0	88.65	0	115.18
8250	49.225	0	58.89	0	143.97

8251	49.225	0	32.47	0	165.51
8252	49.225	0	11.96	0	174.86
8253	49.225	0	0	0	172.9
8254	49.225	0	0	0	170.93
8255	49.225	0	0	0	168.94
8256	49.225	0	0	0.68	155.61
8257	49.225	0	0	15.45	126.8
8258	49.225	0	0	43.91	89.96
8259	49.225	0	0	78.88	52.23
8260	49.225	0	0	113.5	20.39
8261	49.225	0	0	141.23	0.91
8262	49.225	0	0	155.21	0
8263	49.225	0	0	153.25	0
8264	49.225	0	0	151.28	0
8265	49.225	0	0	149.22	0
8266	49.225	0.55	0	138.97	0
8267	49.225	12.78	0	118.42	0
8268	49.225	36.47	0	91.74	0
8269	49.225	65.75	0	62.94	0
8270	49.225	95.04	0	35.79	0
8271	49.225	119.08	0	13.88	0
8272	49.225	132.89	0	0.56	0
8273	49.225	131.58	0	0	0
8274	49.225	129.65	0	0	0
8275	49.225	127.71	0	0	0
8276	49.225	125.78	0	0	0
8277	49.225	123.84	0	0	0
8278	49.225	116.06	0	0	0
8279	49.225	89.98	0	0	0
8227	55.475	124.8	0	158.54	0
8228	55.475	126.27	0	141.1	0
8229	55.475	127.74	0	103.15	0
8230	55.475	129.21	0	57.55	0
8231	55.475	130.67	0	17.76	0
8232	55.475	136.85	0	0	0
8233	55.475	149.59	8.31	0	0
8234	55.475	163.05	30.77	0	0
8235	55.475	170.68	62.65	0	0
8236	55.475	172.52	99.01	0	0
8237	55.475	148.44	134.68	0	0
8238	55.475	76.77	164.27	0	0
8239	55.475	10.69	181.34	0	0
8240	55.475	0	183.21	0	0
8241	55.475	0	185.08	0	0
8242	55.475	0	185.08	0	0
8243	55.475	0	183.21	0	0
8244	55.475	0	181.34	0	7.53
8245	55.475	0	179.47	0	30.03
8246	55.475	0	170.13	0	61.73
8247	55.475	0	144.85	0	96.96
8248	55.475	0	110.02	0	130.29
8249	55.475	0	71.97	0	156.51
8250	55.475	0	36.76	0	170.12
8251	55.475	0	10.2	0	168.25

8252	55.475	0	0	0	166.38
8253	55.475	0	0	0	164.5
8254	55.475	0	0	0	155.42
8255	55.475	0	0	1.3	135.58
8256	55.475	0	0	18.52	108.89
8257	55.475	0	0	49.25	79.09
8258	55.475	0	0	85.44	49.77
8259	55.475	0	0	119.4	24.33
8260	55.475	0	0	143.83	6.03
8261	55.475	0	0	149.56	0
8262	55.475	0	0	147.69	0
8263	55.475	0	0	145.82	0
8264	55.475	1.26	0	141.59	0
8265	55.475	13.59	0	126.84	0
8266	55.475	35.19	0	104.28	0
8267	55.475	61.51	0	77.66	0
8268	55.475	88.19	0	50.5	0
8269	55.475	111.11	0	26.2	0
8270	55.475	126.38	0	7.92	0
8271	55.475	128.47	0	0	0
8272	55.475	126.64	0	0	0
8273	55.475	124.8	0	0	0
8274	55.475	122.97	0	0	0
8275	55.475	121.13	0	0	0
8276	55.475	119.29	0	0	0
8277	55.475	110.37	0	0	0
8278	55.475	83.94	0	0	0
8279	55.475	50.11	0	0	0
8227	61.725	114.94	0	145.74	0
8228	61.725	116.29	0	138.57	0
8229	61.725	117.64	0	108.05	0
8230	61.725	119	0	65.86	0
8231	61.725	120.35	0	25.28	0
8232	61.725	126.03	0	0.19	0
8233	61.725	138.87	9.98	0	0
8234	61.725	151.84	36.71	0	0
8235	61.725	157.2	73.14	0	0
8236	61.725	158.89	111.9	0	0
8237	61.725	146.45	145.31	0	0
8238	61.725	66.53	164.6	0	0
8239	61.725	0.06	166.31	0	0
8240	61.725	0	168.03	0	0
8241	61.725	0	169.74	0	0
8242	61.725	0	162.39	0	0
8243	61.725	0	139.32	0	0.03
8244	61.725	0	107.73	0	8.93
8245	61.725	0	72.93	0	30.16
8246	61.725	0	40.02	0	58.8
8247	61.725	0	13.91	0	90.15
8248	61.725	0	0	0	119.67
8249	61.725	0	0	0	143.02
8250	61.725	0	0	0	156.02
8251	61.725	0	0	0	154.31
8252	61.725	0	0	0	152.59

8253	61.725	0	0	0	141.22
8254	61.725	0	0	0	114.35
8255	61.725	0	0	6.34	79.59
8256	61.725	0	0	41.45	44.23
8257	61.725	0	0	89.13	15.24
8258	61.725	0	0	129.2	0
8259	61.725	0	0	140.59	0
8260	61.725	0	0	138.88	0
8261	61.725	0	0	137.16	0
8262	61.725	0	0	131.04	0
8263	61.725	0	0	107.91	0
8264	61.725	0	0	74.86	0
8265	61.725	3.75	0	40.34	0
8266	61.725	18.57	0	12.37	0
8267	61.725	40.53	0	0	0
8268	61.725	65.4	0	0	0
8269	61.725	89.15	0	0	0
8270	61.725	107.99	0	0	0
8271	61.725	118.31	0	0	0
8272	61.725	116.63	0	0	0
8273	61.725	114.94	0	0	0
8274	61.725	113.25	0	0	0
8275	61.725	111.56	0	0	0
8276	61.725	109.81	0	0	0
8277	61.725	93.6	0	0	0
8278	61.725	61.49	0	0	0
8279	61.725	27.04	0	0	0
8227	67.975	102	0	127.5	0
8228	67.975	103.2	0	119.47	0
8229	67.975	104.4	0	82.05	0
8230	67.975	105.6	0	35.05	0
8231	67.975	106.82	0	1.93	0
8232	67.975	114.67	2.02	0	0
8233	67.975	128.27	16.88	0	0
8234	67.975	138	42.19	0	0
8235	67.975	139.5	72.98	0	0
8236	67.975	134.13	104.05	0	0
8237	67.975	79.62	129.95	0	0
8238	67.975	15.91	144	0	0
8239	67.975	0	145.5	0	0
8240	67.975	0	147	0	0
8241	67.975	0	148.5	0	0
8242	67.975	0	148.5	0	0
8243	67.975	0	137.35	0	0
8244	67.975	0	101.86	0	0
8245	67.975	0	56.78	0	6.67
8246	67.975	0	17.35	0	36.27
8247	67.975	0	0	0	76.9
8248	67.975	0	0	0	114.99
8249	67.975	0	0	0	137.55
8250	67.975	0	0	0	136.5
8251	67.975	0	0	0	135
8252	67.975	0	0	0	133.5
8253	67.975	0	0	0	132

8254	67.975	0	0	0	121.9
8255	67.975	0	0	3.41	92.61
8256	67.975	0	0	20.68	55.25
8257	67.975	0	0	46.86	20.94
8258	67.975	0	0	75.76	0.31
8259	67.975	0	0	101.49	0
8260	67.975	0	0	118.43	0
8261	67.975	0	0	120	0
8262	67.975	0	0	118.5	0
8263	67.975	0	0	109.32	0
8264	67.975	0.83	0	86.46	0
8265	67.975	8.77	0	57.17	0
8266	67.975	22.96	0	28.38	0
8267	67.975	40.86	0	6.71	0
8268	67.975	60.02	0	0	0
8269	67.975	78.14	0	0	0
8270	67.975	93.03	0	0	0
8271	67.975	102.64	0	0	0
8272	67.975	103.5	0	0	0
8273	67.975	102	0	0	0
8274	67.975	100.5	0	0	0
8275	67.975	99	0	0	0
8276	67.975	95.35	0	0	0
8277	67.975	74.6	0	0	0
8278	67.975	43.95	0	0	0
8279	67.975	15.19	0	0	0

5 Typical results: parameter estimation with local methods

In this section we report parameter estimation results obtained with local optimization techniques. Repeated local searches were launched in a multi-start procedure, starting from initial parameter vectors with values chosen randomly from within the parameter bounds. The number of local searches was fixed so that their overall CPU time was comparable to that consumed in optimizations where the global method eSS was used, which are reported in the next section. While there was great variability in the results obtained for the different benchmarks, a conclusion was common to all of them: in all cases, the local methods were outperformed by the global optimization method eSS. In the case of benchmark B1, all the local searches (with DHC, FMINSEARCH, FMINCON) failed to converge. The following figure shows histograms of the results obtained by the local methods (i.e., objective function values reached and the frequency with which they were found) for benchmarks B2–B6.

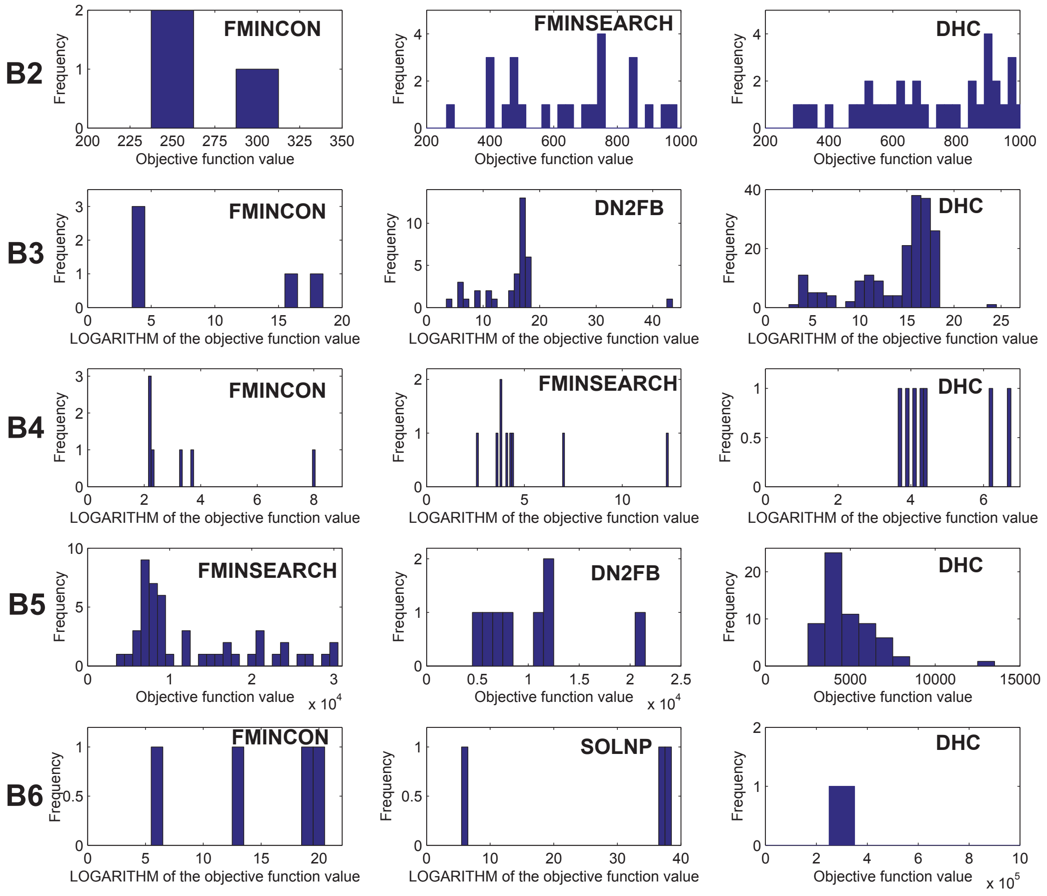

Figure S2. Histograms of local searches. The X axis shows the values of the solutions found by the different local methods, and the Y axis shows their frequency. The computational cost associated to each benchmark and to each method is different, hence the number of local searches performed varies from one method to another:
Benchmark B2: 5 local searches for FMINCON, 50 for FMINSEARCH and DHC. To improve visualization, only the results that achieved an objective function value $f < 1000$ are shown (3/5 for FMINCON, 24/50 for FMINSEARCH, 34/50 for DHC).
Benchmark B3: 100 local searches for FMINCON, 1000 for DN2FB and DHC. Only the results that converged are shown (5/100 for FMINCON, 36/1000 for DN2FB, 188/1000 for DHC).
Benchmark B4: The number of local searches is 10 for every case. Only the results that converged are shown (7/10 for FMINCON, 9/10 for FMINSEARCH, 7/10 for DHC).
Benchmark B5: 10 local searches for FMINSEARCH, 100 for DN2FB and DHC.
Benchmark B6: 50000 local searches for FMINCON and DHC, 100000 for SOLNP. Only the results that converged are shown (3/50000 for FMINCON, 4/100000 for SOLNP, 1/50000 for DHC).

6 Typical results: parameter estimation with eSS

In this section we report parameter estimation results obtained with the eSS method [6] using the AMIGO toolbox implementation [2]. The main settings used with the eSS method are summarized in Table S25 below; they correspond to the default settings of the algorithm. The key element in the eSS algorithm is arguably the so-called Reference Set (RefSet): an evolving set of solutions which are selected for their quality and/or diversity, and which are combined among them to give new solutions (additionally, new solutions can be obtained from local searches launched from existing solutions). Therefore many of the settings refer to the way the RefSet is calculated. In the table, *nvar* stands for the number of unknown parameters. The local method and the maximum CPU time set are benchmark-dependent, and they are specified in Table 2 in the article. For further details of the eSS method the reader is referred to [6].

Table S25. Summary of eSS options

Number of initial solutions generated	10·nvar
Number of elements in RefSet	Positive solution of $b \cdot (b-1) - 10$·nvar= 0, rounded to the closest even number
Type of RefSet initialization	Take bounds, middle point and fill by euclidean distance
Type of combination of RefSet elements	Hyper-rectangles
Type of RefSet regeneration	Regeneration by distance diversity
Max. number of RefSet elements deleted at regeneration	Half of the RefSet elements
Criterion for diversification in the RefSet	Euclidean distance
Number of iterations between local searches	10
Number of function evaluations before 1st local search	100·nvar
Min. number of function evaluations between local searches	200·nvar

Convergence curves of all the benchmarks are shown in figure S3 in the next page. Other results are reported in the subsequent subsections.

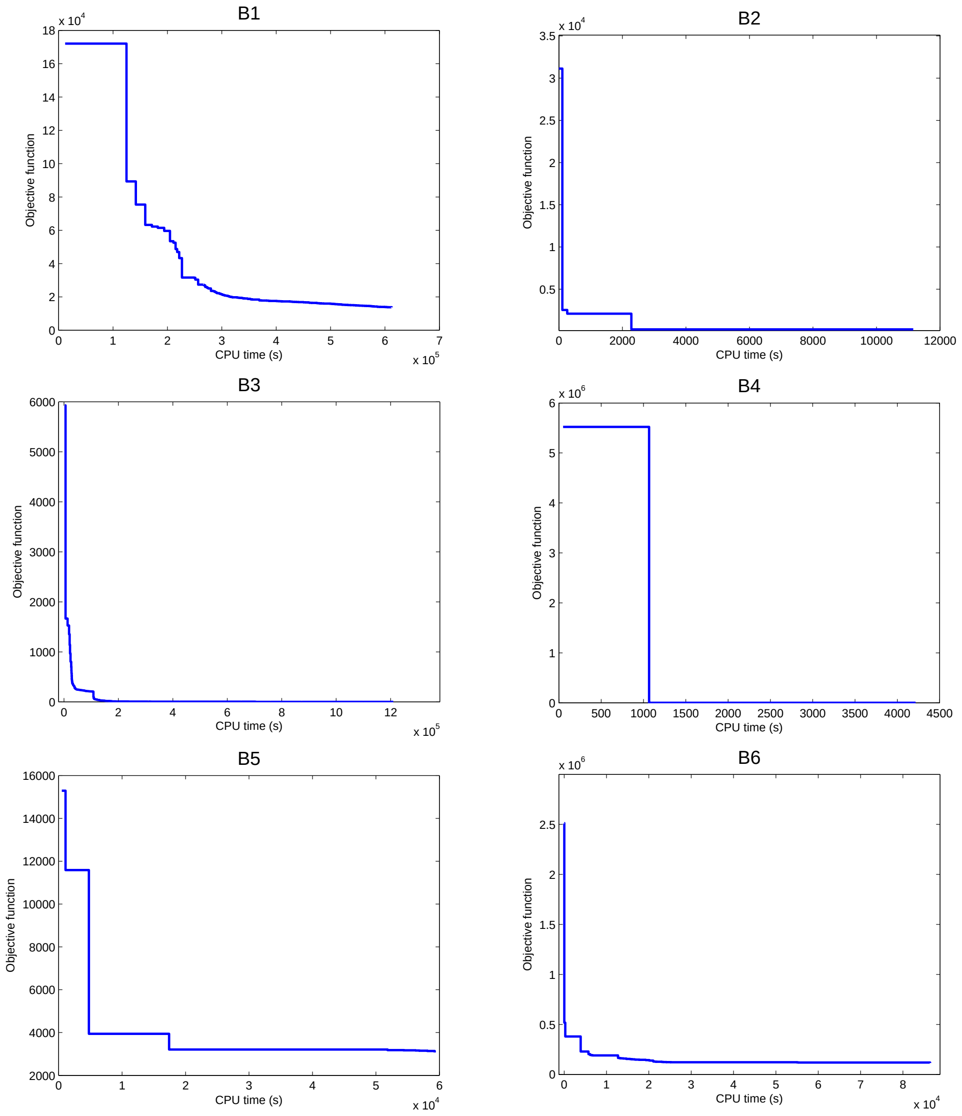

Figure S3. Convergence curves. Result of a typical parameter estimation with the eSS method. The figures plot the objective function value as a function of the computation time.

6.1 B1: *S. cerevisiae*

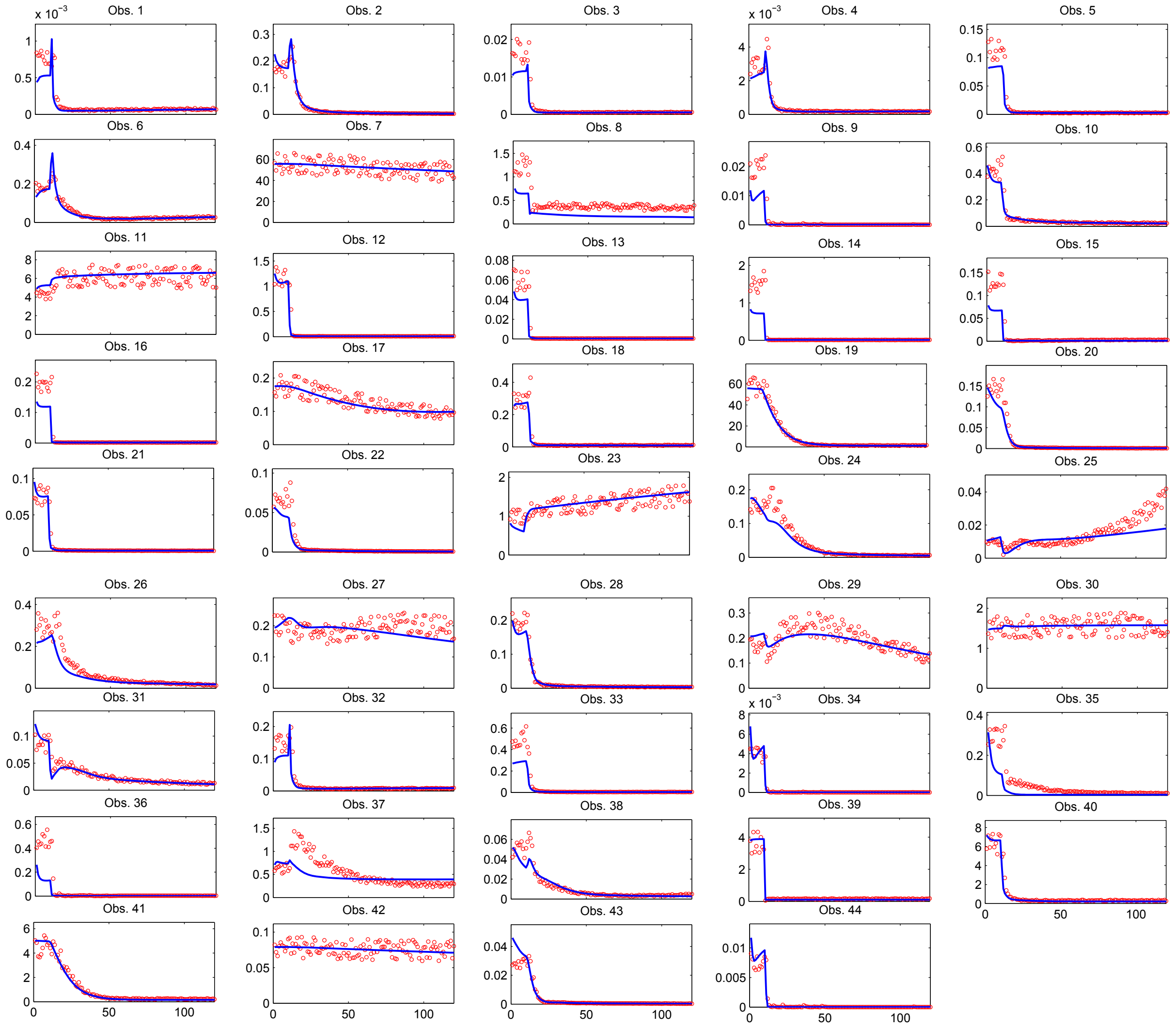

Figure S4. Benchmark 1. Data fits: time courses. Pseudo-experimental data (circles) vs. optimal solution (solid lines) for the 44 observed states. X axis: time [s]; Y axis: metabolite concentrations [mM].

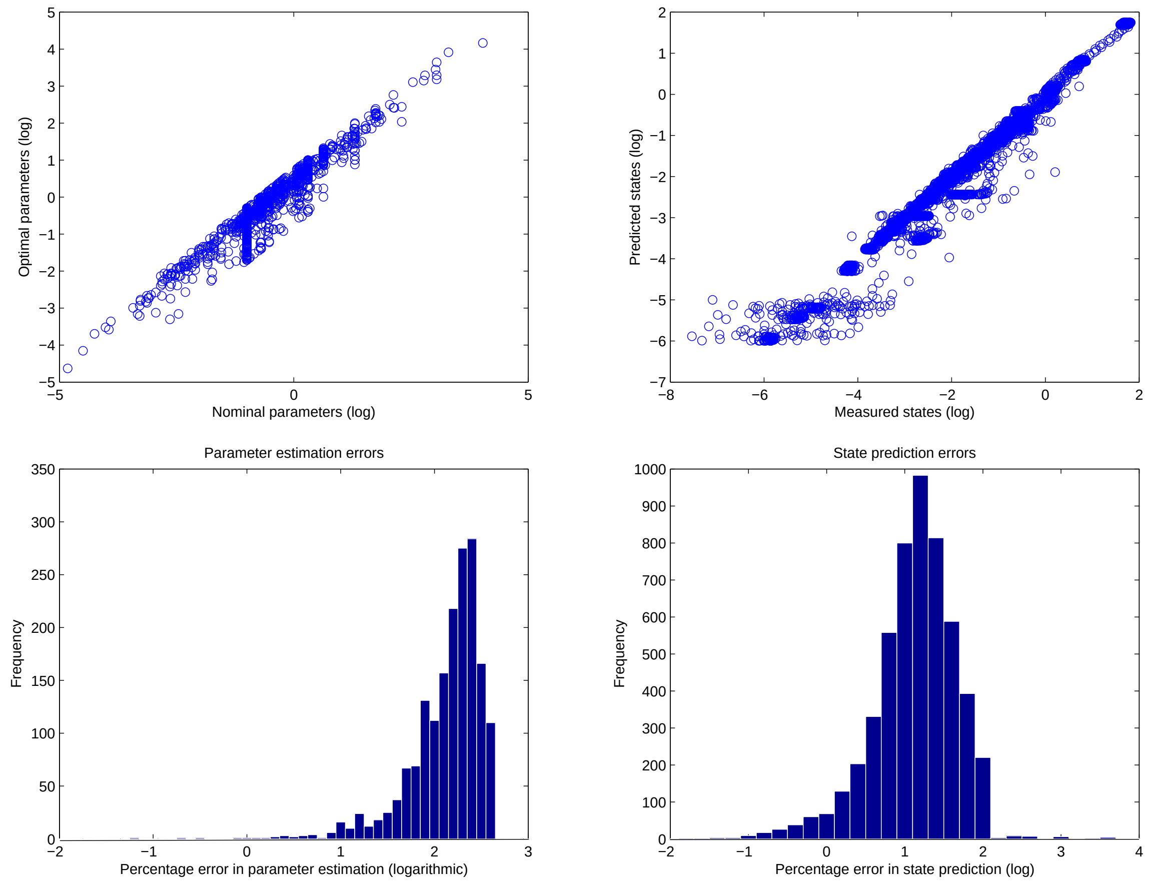

Figure S5. Benchmark 1, typical parameter estimation results. Left side: difference between the nominal parameter vector and the optimal solution. Top left: absolute values. Bottom left: histogram of the differences in %. Right side: data fits (states). Top right: pseudo-experimental vs. simulated data (optimal solution). Bottom right: histogram of the state errors in %.

6.2 B2: dynamic CCM of *E. coli*

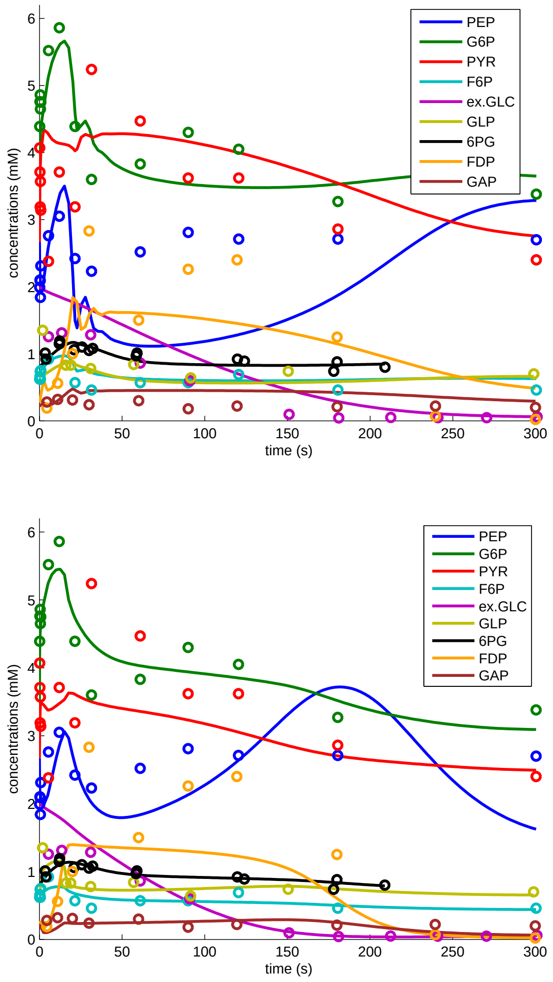

Figure S6. Benchmark 2. Data fits: time courses. Data (circles) vs. model simulations (solid lines), for the 9 metabolites for which there is experimental data available. Top: simulation with the parameter vector reported in the original publication [5]; Bottom: simulation with the optimized parameter vector. Note the improvement in the prediction of PEP and GAP.

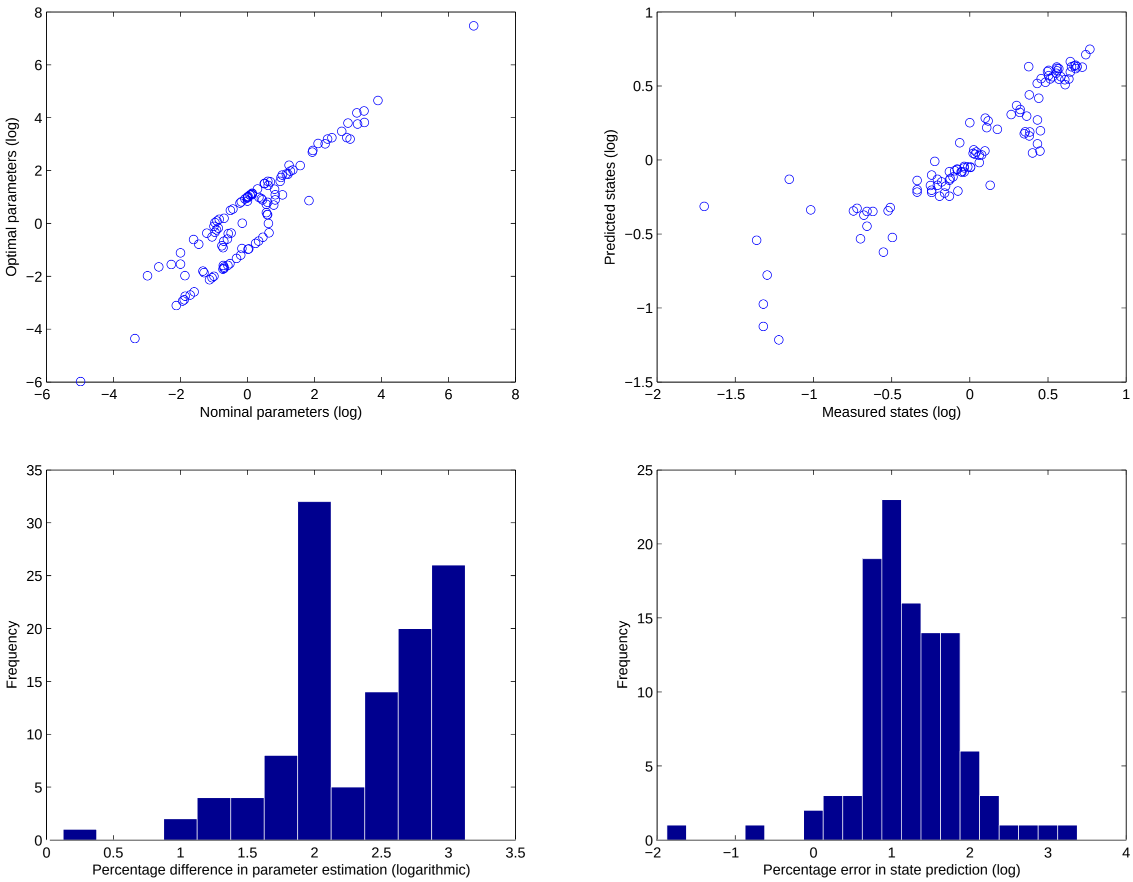

Figure S7. Benchmark 2, typical parameter estimation results. Left side: difference between the original parameter vector and the optimal solution. Top left: absolute values. Bottom left: histogram of the differences in %. Right side: data fits (states). Top right: experimental vs. simulated data (optimal solution). Bottom right: histogram of the state errors in %.

6.3 B3: regulation of the CCM of *E. coli*

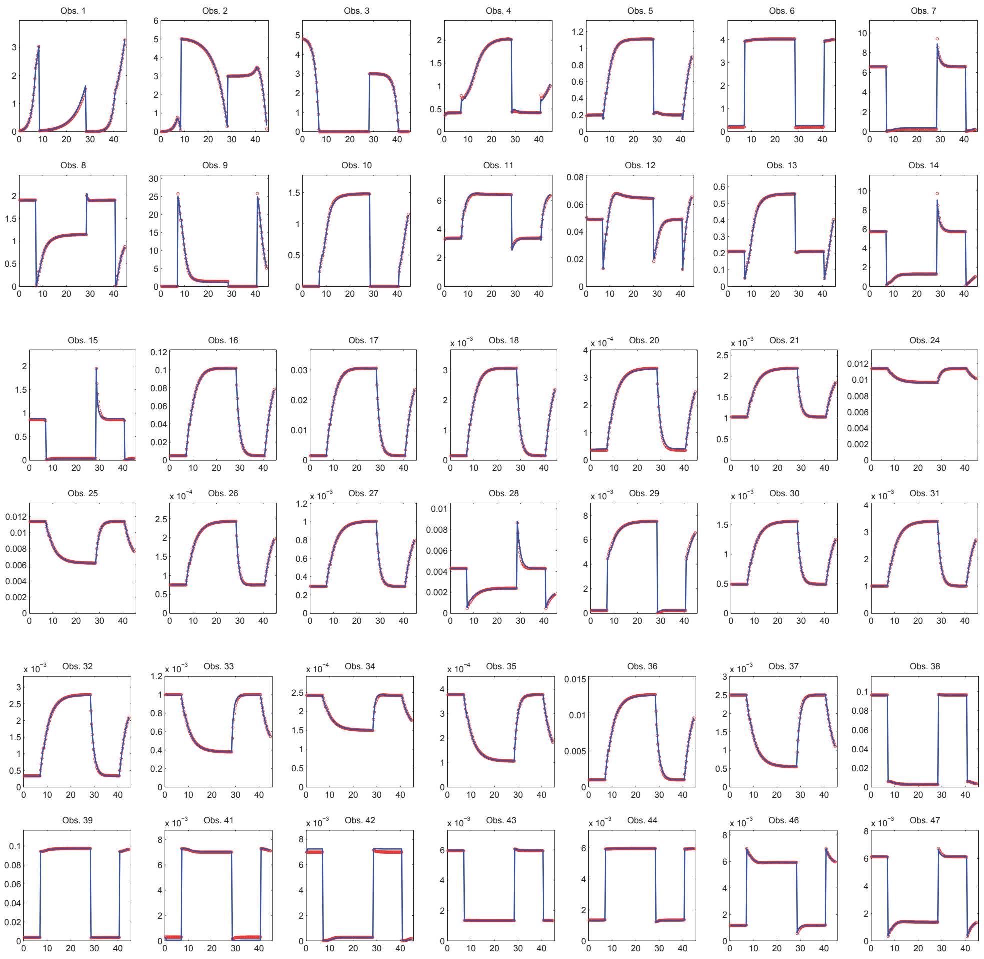

Figure S8. Benchmark 3. Data fits: time courses. Pseudo-experimental data (circles) vs. optimal solution (solid lines) for the 42 metabolites with time-varying concentration. The x axis represents the time in hours, while the y axis represents the magnitude of each state; the units of the states are gl^{-1} for carbon sources, $\frac{\mu mol}{g_{DW}}$ for metabolites, $\frac{g_{Prot}}{g_{DW}}$ for proteins, and [OD] for the biomass concentration.

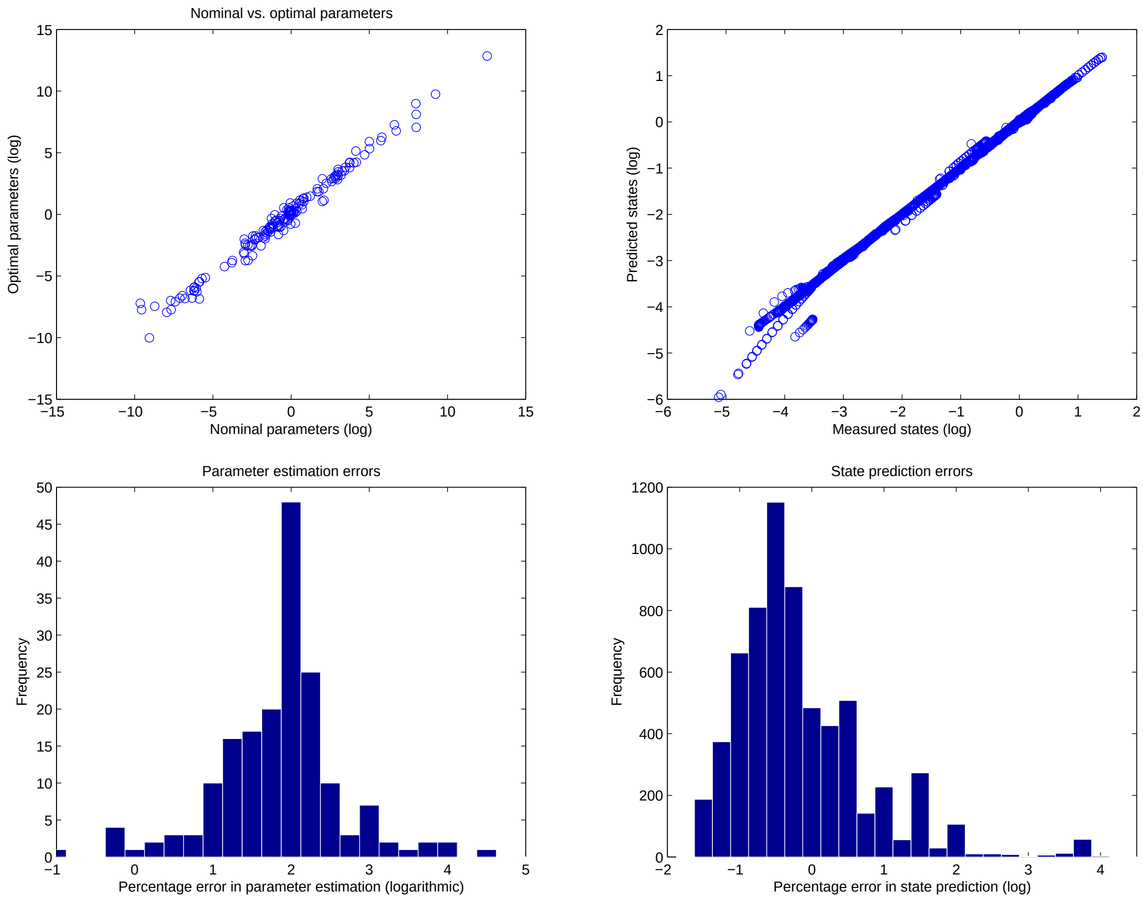

Figure S9. Benchmark 3, typical parameter estimation results. Left side: difference between the original parameter vector and the optimal solution. Top left: absolute values. Bottom left: histogram of the differences in %. Right side: data fits (states). Top right: pseudo-experimental vs. optimal data (optimal solution). Bottom right: histogram of the state errors in %. Note that some states corresponding to very low concentrations ($< 10^{-6}$) are not included in the visualization.

6.4 B4: CHO

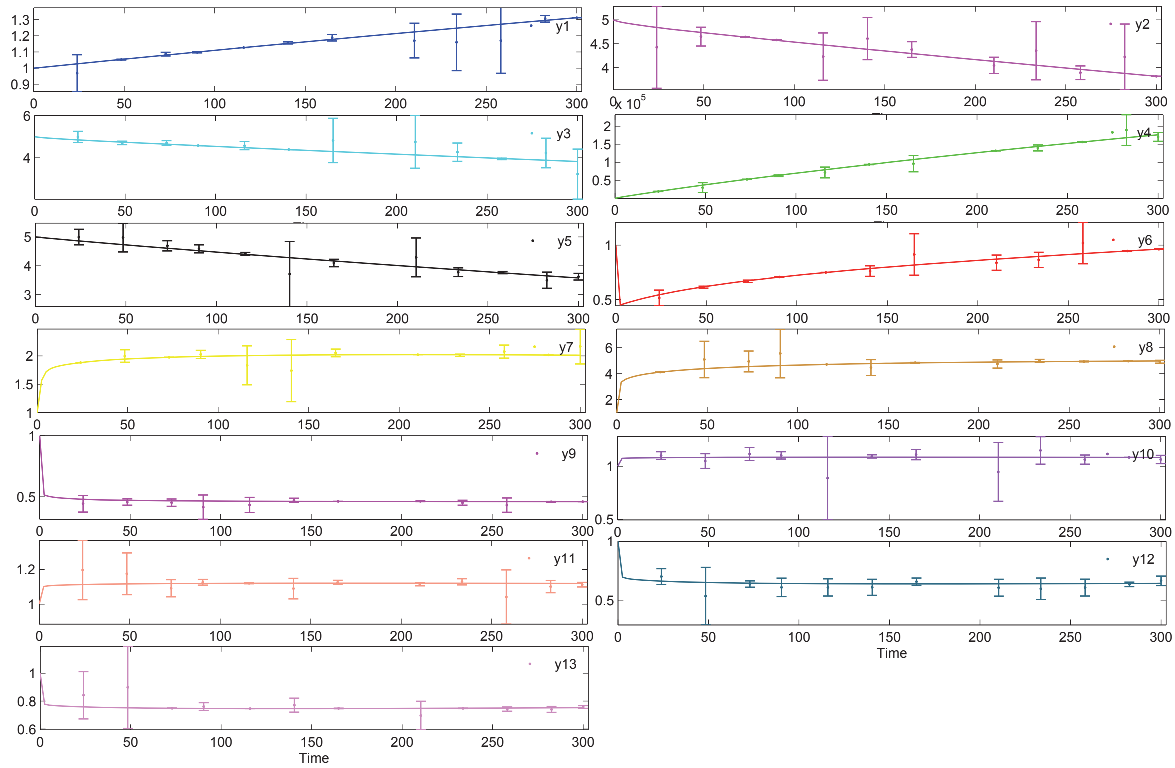

Figure S10. Benchmark 4. Data fits: time courses. Pseudo-experimental data (bars) vs. optimal solution (solid lines) for the 13 observed states. X axis: time [hours]; Y axis: metabolite concentrations [mM].

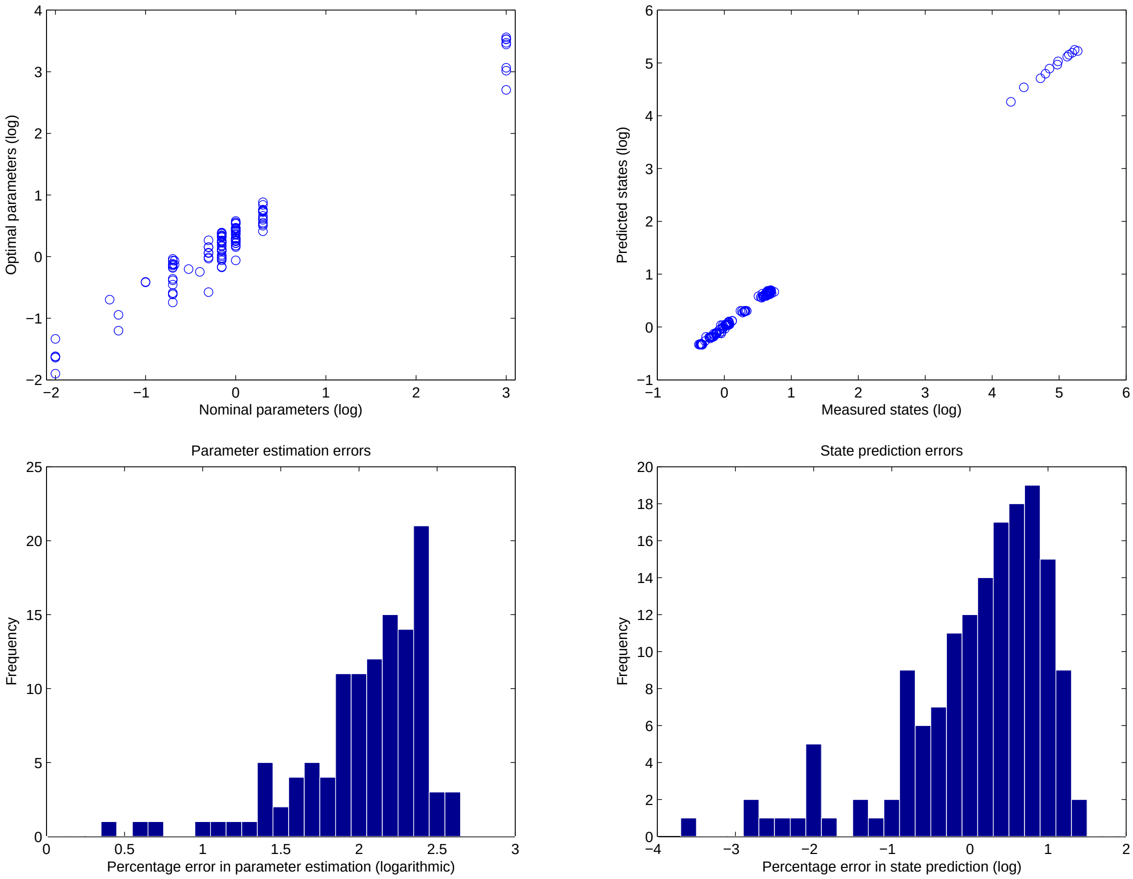

Figure S11. Benchmark 4, typical parameter estimation results. Left side: difference between the nominal parameter vector and the optimal solution. Top left: absolute values. Bottom left: histogram of the differences in %. Right side: data fits (states). Top right: pseudo-experimental vs. simulated data (optimal solution). Bottom right: histogram of the state errors in %.

6.5 B5: signal transduction logic model

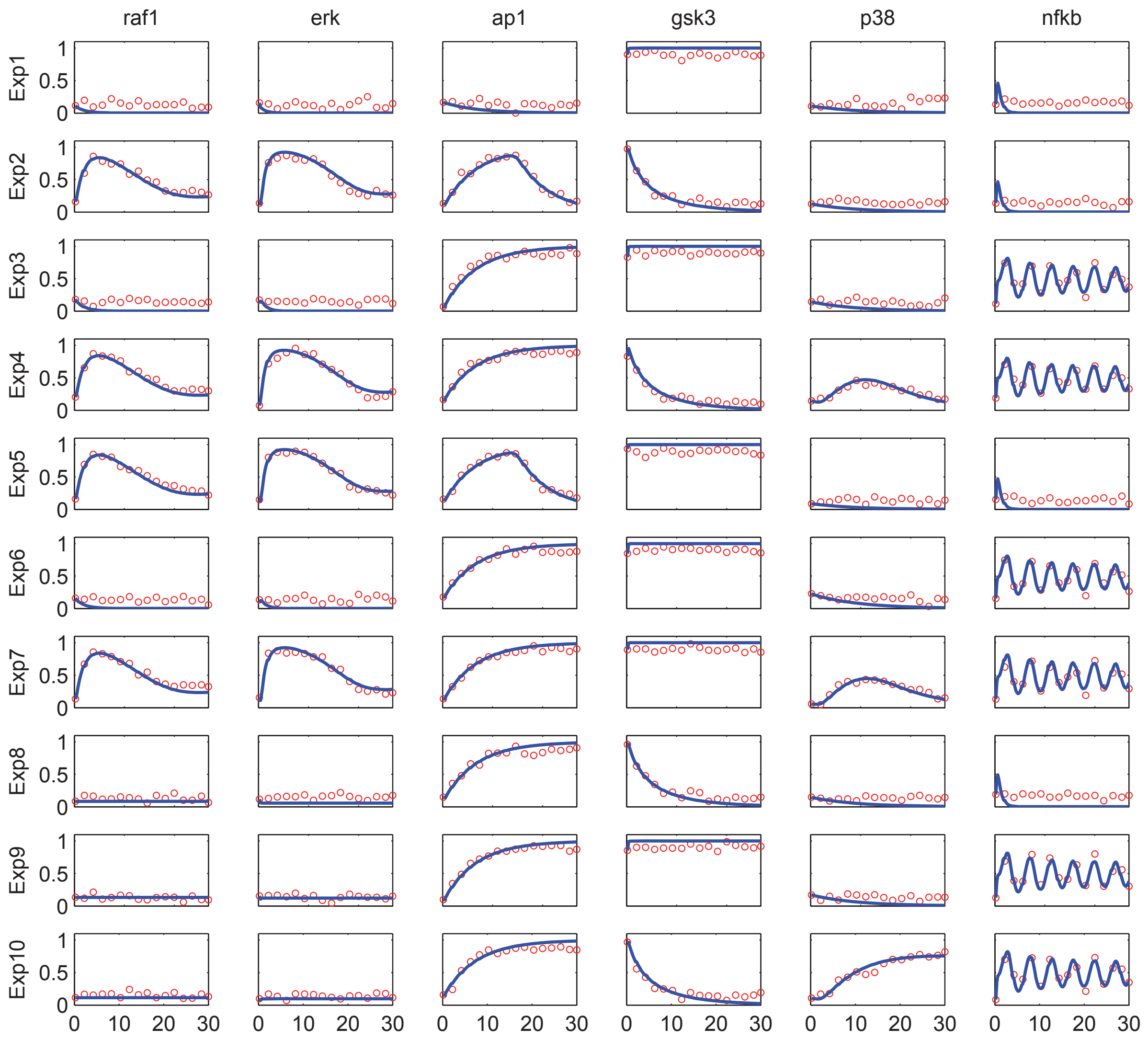

Figure S12. Benchmark 5. Data fits: time courses. Pseudo-experimental data (red stars) vs. optimal solution (solid blue lines) for the 6 observed states. X axis: time [minutes]. Y axis: activation level [0÷1]

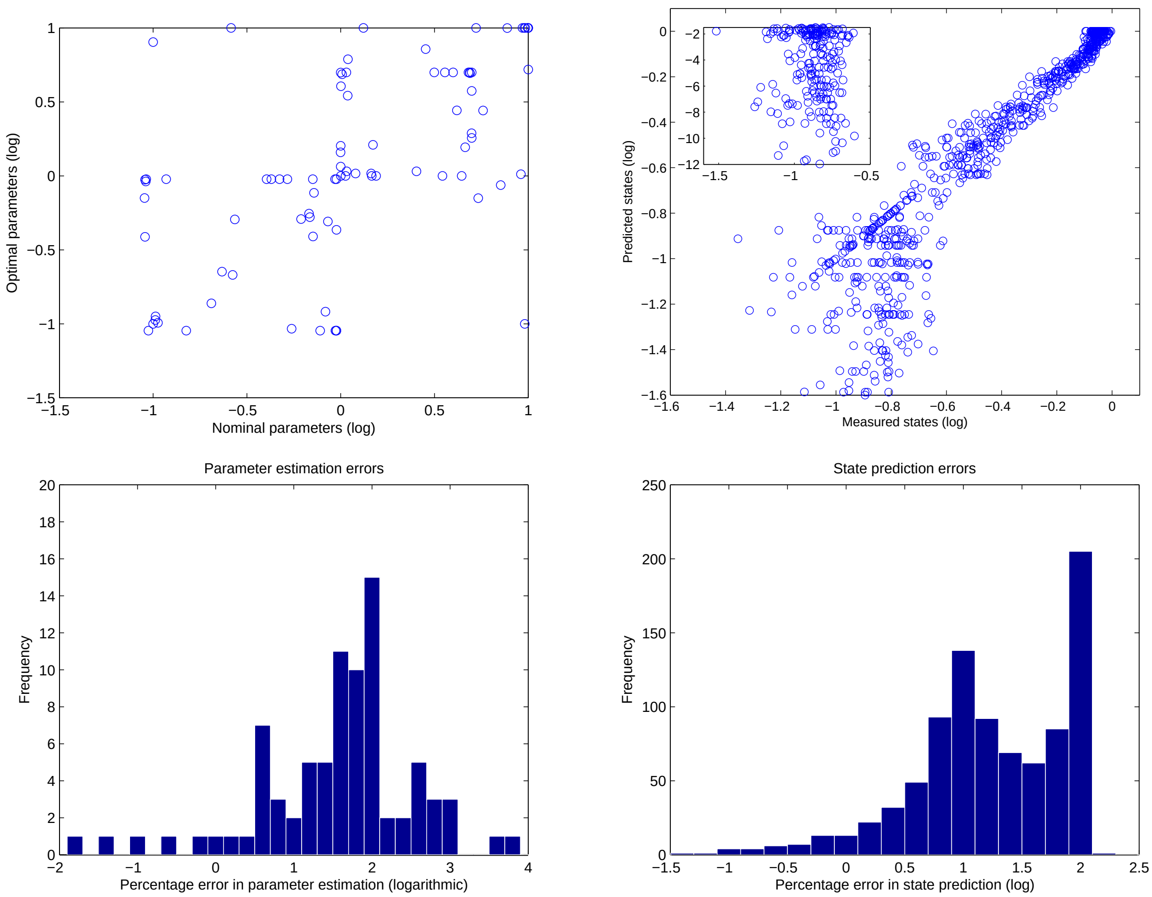

Figure S13. Benchmark 5, typical parameter estimation results. Left side: difference between the nominal parameter vector and the optimal solution. Top left: absolute values. Bottom left: histogram of the differences in %. Right side: data fits (states). Top right: pseudo-experimental vs. simulated data (optimal solution). Bottom right: histogram of the state errors in %.

6.6 B6: *Drosophila*

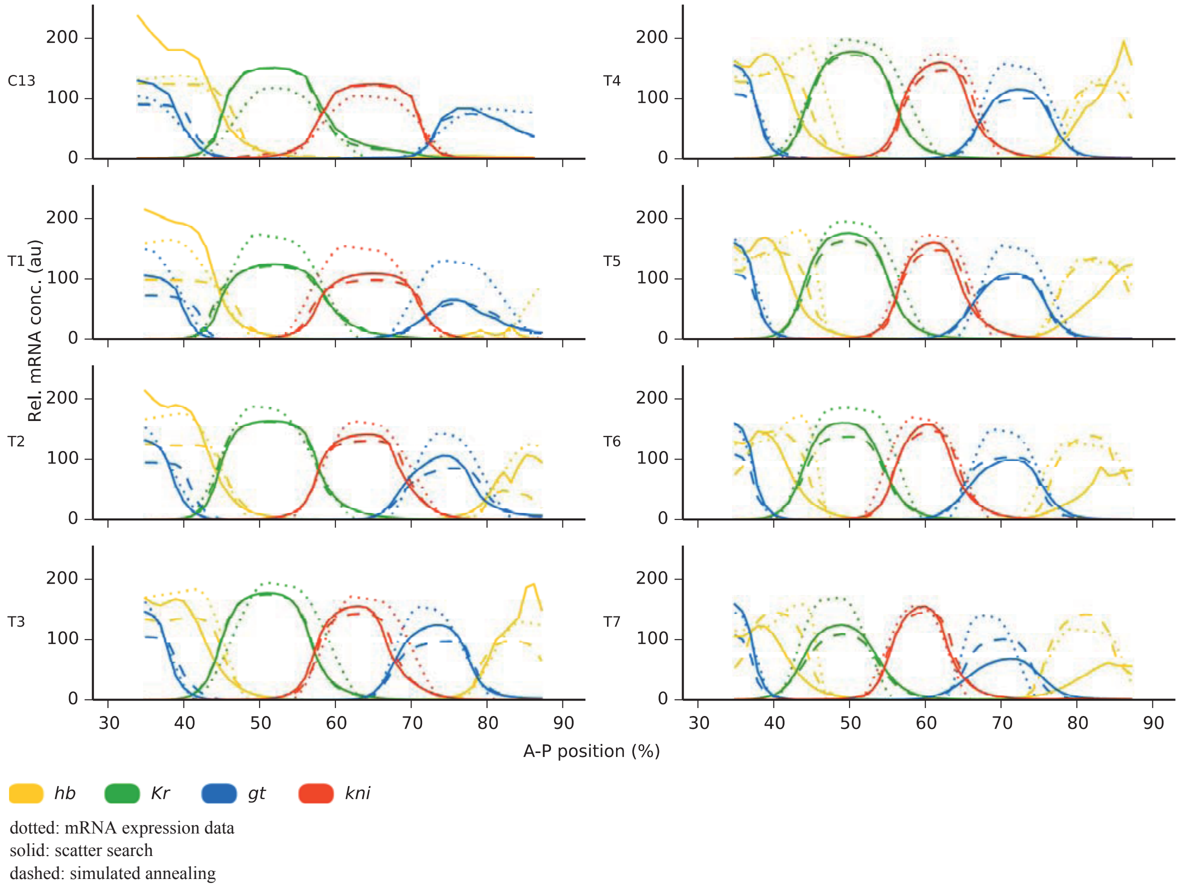

Figure S14. Benchmark 6. Data fits: time courses. Experimental data (dots) vs. optimal solution (solid lines –eSS solution– and dashed lines –simulated annealing solution–) for the 212 observed states corresponding to the 4 genes of the 53 nuclei, at different timepoints. X axis: A-P position [%]. Y axis: relative mRNA concentration.

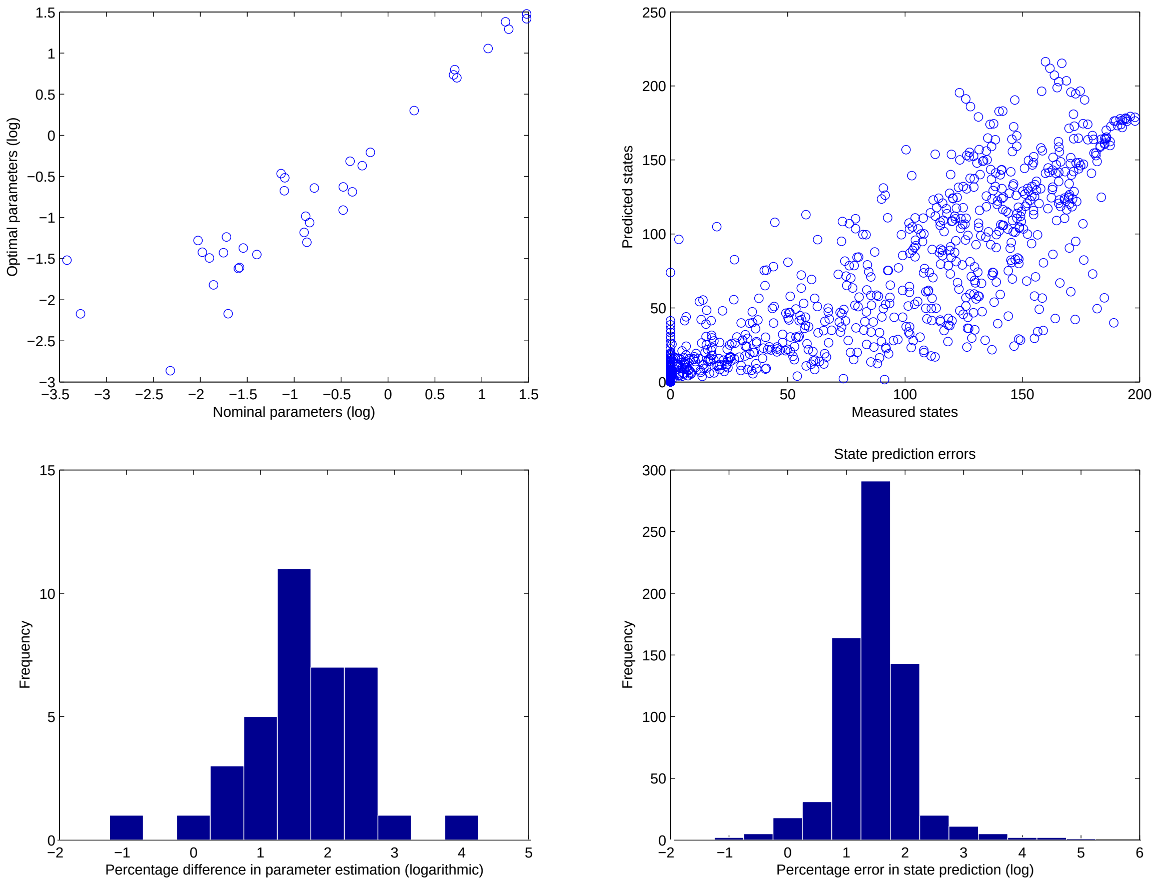

Figure S15. Benchmark 6, typical parameter estimation results. Left side: difference between the nominal parameter vector and the optimal solution. Top left: absolute values. Bottom left: histogram of the differences in %. Right side: data fits (states). Top right: pseudo-experimental vs. simulated data (optimal solution). Bottom right: histogram of the state errors in %.

References

1. Hucka M, Finney A, Sauro HM, Bolouri H, Doyle JC, et al. (2003) The systems biology markup language (sbml): a medium for representation and exchange of biochemical network models. Bioinformatics 19: 524–531.

2. Balsa-Canto E, Banga JR (2011) Amigo, a toolbox for advanced model identification in systems biology using global optimization. Bioinformatics 27: 2311–2313.

3. Hoops S, Sahle S, Gauges R, Lee C, Pahle J, et al. (2006) Copasi – a complex pathway simulator. Bioinformatics 22: 3067–3074.

4. Davies O, Mendes P, Smallbone K, Malys N (2012) Characterisation of multiple substrate-specific (d) itp/(d) xtpase and modelling of deaminated purine nucleotide metabolism. BMB Reports 45: 259–264.

5. Chassagnole C, Noisommit-Rizzi N, Schmid JW, Mauch K, Reuss M (2002) Dynamic modeling of the central carbon metabolism of escherichia coli. Biotechnol Bioeng 79: 53–73.

6. Egea J, Henriques D, Cokelaer T, Villaverde A, MacNamara A, et al. (2014) Meigo: an open-source software suite based on metaheuristics for global optimization in systems biology and bioinformatics. BMC Bioinformatics 15: 136.
